# Sox11 promotes head and neck cancer progression via the regulation of SDCCAG8

**DOI:** 10.1186/s13046-019-1146-7

**Published:** 2019-03-29

**Authors:** Junwei Huang, Eoon Hye Ji, Xinyuan Zhao, Li Cui, Kaori Misuno, Mian Guo, Zhigang Huang, Xiaohong Chen, Shen Hu

**Affiliations:** 1 School of Dentistry, University of California, Los Angeles, CA 90095 USA; 2 Jonsson Comprehensive Cancer Center, University of California, Los Angeles, CA 90095 USA; 3 Department of Otorhinolaryngology, Key Laboratory of Otolaryngology Head and Neck Surgery, Beijing Tongren Hospital, Capital Medical University, Beijing, 100730 China; 4 Department of Endodontics, Stomatological Hospital, Southern Medical University, Guangzhou, 510280 China; 5 Department of Neurosurgery, The Second Affiliated Hospital of Harbin Medical University, Harbin, 150086 China

**Keywords:** Head and neck squamous cell carcinoma, SOX11, SDCCAG8, Proteomics, Invasion

## Abstract

**Background:**

SOX11 is a transcription factor that plays an important role in mantle cell lymphoma development. However, its functional role in head and neck squamous cell carcinoma (HNSCC) remains unknown.

**Methods:**

Protein expression was measured with Western blotting, immunohistochemistry or quantitative proteomics, and gene expression was measured with quantitative RT-PCR. Functional role of SOX11 in HNSCC was evaluated with MTS/apoptosis, migration, invasion assays and a xenograft model. A SOX11-targeting gene, SDCCAG8, was confirmed with chromatin immunoprecipitation (ChIP), luciferase reporter and rescue assays.

**Results:**

SOX11 was up-regulated in recurrent versus primary HNSCC and in highly invasive versus low invasive HNSCC cell lines. Silencing SOX11 in HNSCC cell lines significantly inhibited the cell proliferation, migration, invasion and resistance to Cisplatin, and vice versa. Quantitative proteomic analysis of SOX11-silencing HNSCC cells revealed a number of differentially expressed proteins, including a down-regulated tumor antigen SDCCAG8. Silencing of SDCCAG8 in HNSCC cells also significantly inhibited the cell proliferation, migration and invasion, and vice versa. ChIP assays demonstrated that endogenous SOX11 strongly bound to *Sdccag8* gene promoter in highly invasive HNSCC cells. When over-expressed in low invasive HNSCC cells, wild type SOX11 but not mutant SOX11 induced the promoter activity of *Sdccag8* and significantly induced the expression of SDCCAG8. However, exogenous mutant SOX11 abolished the expression of SDCCAG8 in highly invasive HNSCC cells. In addition, the inhibitory effects of SOX11 knockdown were partially rescued by over-expression of SDCCAG8 in HNSCC cells.

**Conclusion:**

Collectively, our findings indicate SOX11 promotes HNSCC progression via the regulation of SDCCAG8.

**Electronic supplementary material:**

The online version of this article (10.1186/s13046-019-1146-7) contains supplementary material, which is available to authorized users.

## Introduction

Head and neck squamous cell carcinoma (**HNSCC**) is the sixth most common type of cancer in the world, accounting for approximately 650,000 new cancer cases and 350,000 deaths annually [1]. Due to advances in surgery, radiotherapy, chemotherapy and immunotherapy for HNSCC treatment, effective functional and survival outcomes are seen in patients with early-stage HSNCC. However, patients with advanced-stage disease continue to suffer from poor survival [2, 3]. This is because the patients with HNSCC are often diagnosed with lymph node metastasis and consequently have a high recurrence rate after treatment. The high mortality rate of HNSCC patients highlights the importance of studying the molecular and cellular mechanisms underlining HNSCC progression and identifying novel targets for improved treatment, screening, and surveillance.

Members of the *Sox* (SRY-related HMG-box) gene family were first identified through homology of the HMG domain to the testis-determining factor, SRY [4]. The protein products of *Sox* gene family regulate transcription during many diverse developmental processes such as early embryogenesis, sex determination, neural development, cardiac development, and hemopoiesis. *Sox11* is a member of the Group C within the *Sox* family, along with *Sox4* and *Sox12*. Sox members that belong to the same group share a high degree of similarity both within and outside the HMG box domain, whereas members of different groups are less similar. SOX11 is widely expressed at variable levels in developing mouse embryos with the highest expression in neuronal and mesenchymal tissues [5]. The protein has demonstrated a variety of specific functions including regulation of epithelial-mesenchymal interactions, inductive tissue remodeling, neuronal determination and differentiation, kidney and lung development [6], sensory neuron survival and axonal outgrowth [7].

A number of studies have revealed that SOX11 promotes tumor progression in mantle cell lymphoma (**MCL**) [8–17] as well as in melanoma and breast carcinoma [18, 19]. Over- expression of SOX11 was also found in small cell lung cancer [20], leukemia and Burkett’s lymphoma [21]. However, contradicting findings of SOX11 have been reported in ovarian cancer, mantle cell lymphoma and glioma [22–27]. These existing studies imply that the role of SOX11 in tumor development and progression may be tissue specific and cell context dependent. Nevertheless, the functional role of SOX11 in oral/head and neck cancer remains unknown. The main purpose of our study is to investigate if SOX11 promotes the progression of oral/head and neck cancer.

## Material and methods

### Cell culture

Four HNSCC cell lines, UM1, UM2, UMSCC5, UMSCC6, were cultured in the Dulbecco’s modified Eagle’s medium (DMEM) supplemented with 10% fetal bovine serum, 100 U/mL penicillin G and 100 μg/mL streptomycin (Invitrogen, Carlsbad, CA). Cell cultures were maintained in a humidified atmosphere of 5% CO_2_, 95% air at 37 °C, and the culture medium was changed every 2–3 days.

### Real-time quantitative PCR (qPCR)

Total RNA was isolated from cultured cells using the RNeasy Mini Kit (Qiagen, Valencia, CA), and first-strand complementary DNA synthesis was performed using the SuperScript III Reverse Transcriptase (Invitrogen). The cDNA levels were then amplified with the Light Cycler® 480 SYBR Green I MasterMix (Roche, Indianapolis, IN) using the CFX96 Real-Time PCR detection system (Bio-Rad, Hercules, CA). Gene primers for qPCR reactions are listed in Additional file 1: Table S1. Gene expression was measured in triplicates and normalized against beta-actin. The relative expression of each target gene was calculated via the eq. 2^-ΔΔC^_T_ where ΔC_T_ = C_T_ (target) – C_T_ (actin).

### Western blotting

Equal amount of total proteins from each sample were separated with SDS-PAGE gel and transferred onto nitrocellulose membrane with the Trans-blot SD semi-dry transfer cell (Bio-Rad, Hercules, CA). The membrane was blocked with 5% non-fat dry milk (Santa Cruz Biotech, Dallas, TX) for one hour, and then incubated with anti-SOX11 (Santa Cruz Biotech, #Sc-20,096), anti-SDCCAG8 (GeneTex, Irvine, CA, # GTX115484) or anti-GAPDH (GeneTex, #GTX100118) primary antibody, followed by incubation with HRP-conjugated secondary antibody (GE Healthcare, Piscataway, NJ). Signal detection was performed with the ECL-Plus Kit (GE Healthcare) and protein bands were quantified with the NIH Image J.

### Gene silencing and cell line xenograft

Cells were cultured in 6-well plates for siRNA or shRNA knockdown experiments. RNAimax was used for cell transfection with siRNA according to the manufacturer’s instruction (Invitrogen). Validated double-stranded siRNAs of target genes, siSOX11 (Sigma, St. Louis, MO), si-SDCCAG8 (Santa Cruz Biotech, #SC-78905) or non-target control siRNAs were mixed with the RNAimax reagent and then added to the cell culture. After 24-h treatment, the siRNAs were removed and the cells were further incubated in fresh complete medium for subsequent experiments. Transfection of Sox11 shRNAs (Thermo Scientific, RHS4533, pLKO.1-Sox11) was performed with the Lipofectamine 2000 (Invitrogen, #11668019). After 48 h post-transfection, transfected cells were incubated in the presence of puromycin at 2 μg/mL for 2 weeks to generate stably transfected cells. Single colonies with low SOX11 expression were then selected for further studies. The efficacy of knockdown was evaluated by Western blotting.

To assess the xenograft tumor growth, 2 × 10^6^ UM1shControl and UM1shSox11 cells were injected s.c. into the left and right flanks, respectively, of 12 8-week-old Balb/C male nude mice. At 4 weeks post-injection, the mice were euthanized and tumors were collected. The tumor tissues were lysed with 2-D rehydration buffer containing 2 M thiourea, 7 M urea, 2% CHAPS and 50 mM DTT, and the total protein concentration was measured with the 2D Quant kit (GE Healthcare).

### Plasmids and gene overexpression

Transfection of HNSCC cell lines with plasmids was performed with the Lipofectamine 2000. The Sox11 plasmids (pCMV-Sox11F) and mutant versions lacking the transactivation domain (pCMV-Sox11FΔTAD) were generous gifts from Professor Angie Rizzino [28] and Professor Kathryn Albers [29]. Sox-11F was constructed using the primers Sox-11F (5′-CGTGCTGGTACCGCCACCATGGACTACAAGGACG ACGATGATATGGTGCAGCAGGCCGAGAGC-3′) and Sox-11FTAD was constructed using the primer pair sox11TAD (5′-CTCTACTACAGCTTCAAGTGAGCGGCCGCAA ACATCACCAAGCAGCAG-3′) as described in previous study [30]. Plasmid for human Sdccag8 (pEGFP-hSdccag8) was generously provided by Prof. Song-Hai Shi and Mr. Zhizhong Li [31]. For rescue experiment, Sox11 shRNA and sdccag8 plasmid were co-transfected with the Lipofectamine 2000 according to a co-transfection protocol recommended by the manufacturer. Briefly, appropriately amount of shRNA and plasmid mixture (within serum free medium) was combined with diluted Lipofectamine 2000 (in serum free medium) and incubated at room temperature for 20 min. The complex was then added to cell cultures and cells were harvested 48 h after transfection for subsequent experiments.

### Cell growth, survival, migration, invasion assays

#### Cell growth/survival

For cell viability assay, cancer cells were cultured in a 96-well plate overnight before the addition of the MTS solution (tetrazolium compound and phenazine methosulfate, Promega). Next, the cells were incubated at 37 °C for 1 h in a humidified 5% CO_2_ incubator, and the absorbance at 490 nm was recorded with a microplate reader (BioTek, Winooski, VT). The assays were performed in quadruplicate. To assay clonogenicity, 1000 cells were initially seeded per well in a 6-well plate and then fixed and stained with 0.5% crystal violet solution after 7 days of culture. Colonies with a diameter > 50 μm were counted. For chemoresistance study, cells were cultured in a 96-well plate overnight at a concentration of 2000 cells/mL per well and treated with the indicated concentrations of cisplatin (0, 2.5, 5.0, 10.0, 20.0, and 40.0 μM) for 24 h. Then, 20 μL of MTS solution was added to each well, followed by a 1-h incubation at 37 °C. The reaction was then quantitatively measured at 490 nm.

#### Cell migration

Cells were grown to 80% confluence in 24-well plates and then transfected with siRNA, shRNA or respective controls. Cells were starved overnight in DMEM + 0.1% FBS medium. The cellular layer was wounded using a sterilized tip. Cell migration was monitored after 24 h and 48 h. The cells were stained with calcein acetoxymethyl ester (4 μg/ml; BD Biosciences) in phosphate-buffered saline for 30 min and observed by fluorescence microscopy (Olympus). Migration ability was assessed by measuring the changes in sizes of wounded areas.

#### Cell invasion

The invasion assays were performed with the Transwell Matrigel invasion chambers (pore size 8 μm; BD Biosciences). Following 24-h serum starvation, the cancer cells were resuspended in DMEM containing 0.1% FBS and added to the upper chamber of transwell inserts. DMEM supplemented with 10% FBS was then added to the lower chamber to act as a chemoattractant. After incubation for 12 h or 24 h, the cells on the upper surface of the membrane were removed by a cotton swab. The invaded cells on the lower membrane were stained with calcein acetoxymethyl ester (4 μg/ml; BD Biosciences) in phosphate-buffered saline for 30 min and then observed by fluorescence microscopy (Olympus) or stained with the HEMA 3 staining kit (Thermo Fisher). We imaged six fields per filter and counted the average numbers of cells per field as a measure for cell invasion.

### Quantitative proteomic analysis

Quantitative proteomic analysis was performed using tandem mass tagging (TMT) (TMT-6plex, Thermo Fisher Scientific, Waltham, MA) and two-dimensional LC with MS/MS according to our previously described method [32]. Briefly, cancer cells were lysed in 8 M urea containing protease inhibitor cocktail (Calbiochem, San Diego, CA) on ice with a POLYTRON homogenizer (Kenematika, Bohemia, NY). Equal amounts of proteins (100 μg) from either siSOX11- or siCtrl-transfected cells were reduced with dithiothreitol, alkylated with iodoacetamide and digested with trypsin overnight. The resulting peptide samples were then labeled with TMT-128 (UM1 cells, siCtrl), TMT-129 (UM1 cells, siSox11), TMT-130 (UMSCC5 cells, siCtrl) or TMT-131 (UMSCC5 cells, siSox11), respectively, according to the manufacturer’s protocol. Afterwards, we combined the labeled samples and fractionated the combined sample with a strong-cation exchange spin column (VIVAPURE Smini H, Sartorius Stedim, Bohemia, NY). The initial filtrate and eight elutions under different concentrations of sodium acetate (2.5 mM, 5 mM, 10 mM, 20 mM, 50 mM,100 mM, 250 mM and 1 M) were collected, vacuum-dried and re-suspended in 0.1% formic acid for LC-MS/MS analysis.

Fractionated TMT-labeled peptide samples were loaded onto an Agilent nanotrap column (Santa Clara, CA), washed for 10 min at 6 ul/min and then separated with a Microm 100 × 0.1 mm C18AQ column (200A°, 3 μm) on an Eksigent 2DLC nanoflow system operating at 400 nL/min. Chromatography was performed using mobile phase A (0.1% formic acid) and mobile phase B (99.9% ACN, 0.1% formic acid) over a 90-min gradient: (0–30% B (60 min), 35–80% B (10 min), 80% B (5 min), and then the column was re-equilibrated. The MS/MS spectra were acquired on an Orbitrap LTQ XL mass spectrometer (Thermo Fisher Scientific, San Jose, CA) using a data-dependent analysis mode, which the top five most abundant ions in each MS scan (m/z 350–2000) were selected for MS/MS analysis. The MS/MS spectra were searched against the UniRef100 human database using the SEQUEST search engine via the Proteome Discoverer (Thermo Fisher Scientific). The parameters for SEQUEST database searching were as follows: missed cleavage was one; the dynamic modifications were oxidation (M), deamidation (N) and phosphorylation (S, T, Y), and the static modifications were TMT-6plex (any N-terminus and K) and Carbamidomethyl (C). The false discovery rate was below 1% for protein/peptide identification. The relative quantitation was calculated as the average fold changes of identified proteins in siSox11-transfected cells as compared to the average fold changes of the proteins in siCtrl-transfected cells. The median ratio was used for normalization [33].

### Chromatin immunoprecipitation (ChIP)

The ChIP assays were performed on UM1 and UMSCC5 cells with the ChIP assay kit (Millipore, Billerica, MA, #17–295) according to the manufacturer’s instruction. After PBS washing, the cancer cells were cross-linked with 1% formaldehyde on the petri dish for 10 min at room temperature and then stopped by adding 125 mM glycine. Cells were then washed twice with cold PBS, harvested in RIPA buffer (150 mM NaCl, 1% Igepal CA-630, 0.5% deoxycholate, 0.1% SDS, 50 mM Tris-HCl at pH 8), and sonicated to generate DNA fragments between 200 and 1000 base pairs. For immunoprecipitation, 1 mg of protein extract was pre-cleared with 30 μL of Protein A Agarose/Salmon Sperm DNA (50% Slurry) for 30 min. Once the agarose centrifuged and removed, the supernatant fraction was incubated with 5 μg of anti-SOX11 (Santa Cruz Biotech, #Sc-20,096) overnight at 4 °C and then incubated with 30 μL Protein A Agarose/Salmon Sperm DNA (50% Slurry) for one hour at 4 °C. Following the washes with low salt, high salt, LiCl immune complex wash buffers as well as the TE buffer, the immunocomplexes were harvested, eluted with 1% SDS/0.1 M NaHCO_3_ for 10 min at 65 °C, and then treated with NaCl (200 mM) at 65 °C for 4 h to reverse histone-DNA crosslinks. Finally, the DNA fragments were purified with a DNA Clean/Concentrator kit (Zymo Research, Irvine, CA, #D4003) and used as templates for qPCR reactions using the primers of *Sdccag8* gene promoter (Supplementary Table 1).

### Luciferase reporter assay

Luciferase reporter assays were performed to investigate if SOX11induces the promoter activity of *Sdccag8* gene in HNSCC cells. We utilized a plasmid for wild type Sox11 (Sox11F) and a mutant version, Sox11ΔTAD, as well as *Sdccag8* gene promoter reporter plasmid for co-transfection. To test whether SOX11 induces *Sdccag8* gene promoter activity, UM2 and UMSCC6 cancer cells were seeded in 24-well plates. When reaching ~ 80% confluency, the cells were transfected with either empty promoter reporter vector (pLightSwitch_Prom, #S790005, 200 ng, SwitchGear Genomics, Carlsbad, CA), or *Sdccag8* gene promoter reporter vector (*Sdccag8*-Prom, #S706980, 200 ng, SwitchGear Genomics), or *Sdccag8*-Prom (200 ng) with Sox11F plasmid (100 ng), or *Sdccag8*-Prom (200 ng) with Sox11FΔTAD plasmid (100 ng) using the Lipofectamine 2000 transfection reagent according to the manufacturer’s protocol. Briefly, after the cancer cells were washed with PBS, a mixture of lipofectamine, plasmids and serum-free DMEM was added to the cell culture and incubated at room temperature for 20 min. The treated cells were then incubated in the CO_2_ incubator and the medium was changed after 4 h of treatment. Afterwards, the cells were incubated in fresh complete medium and finally lysed with 100ul of lysis buffer, which is mixed with reconstituted assay substrate of the LightSwitch Luciferase Assay Kit (#LS010, Switchgear Genomics) to determine the promoter activity. All experiments were performed in triplicates. Similar Luciferase reporter assay experiments were also performed on UM1 and UMSCC5 cells.

### Tissue samples and immunohistochemistry

Frozen tissue specimens of HNSCC patients (*n* = 20) and adjacent normal tissues (*n* = 8) were obtained from the Capital Medical University affiliated Tongren Hospital and the Second Affiliated Hospital of Harbin Medical University, Harbin, China, according to established core procedures and the Institutional Ethical Board approvals. Tissue samples were stained with hematoxylin/eosin to determine the histological type and grade of tumors. For immunohistochemistry analysis, formalin-fixed paraffin-embedded (FFPE) tissue sections of HNSCC patients were deparaffinized by sequential washing with xylene, 100% ethanol, 95% ethanol, 80% ethanol and PBS. The endogenous peroxidase activity was quenched in methanol with 0.3% H_2_O_2_ for 5 min. The slides were blocked in PBS with 5% BSA for 30 min and then incubated overnight at 4 °C with rabbit antibody against human SOX11 at a dilution of 1:100. After rinsing in PBS, the sections were incubated with horseradish peroxidase (HRP)-conjugated sheep anti-rabbit IgG for 2 h at room temperature.

### Statistical analysis

Statistical analysis was performed with the student t-test and one-way ANOVA using the MedCalc Program (MedCalc Inc., Ostend, Belgium). The data were expressed as the mean ± standard deviation. *P* values < 0.05 were considered to be statistically significant for all data analyses.

## Results

### Upregulation of SOX11 in primary and recurrent HNSCC tissues

**Fig. 1:**
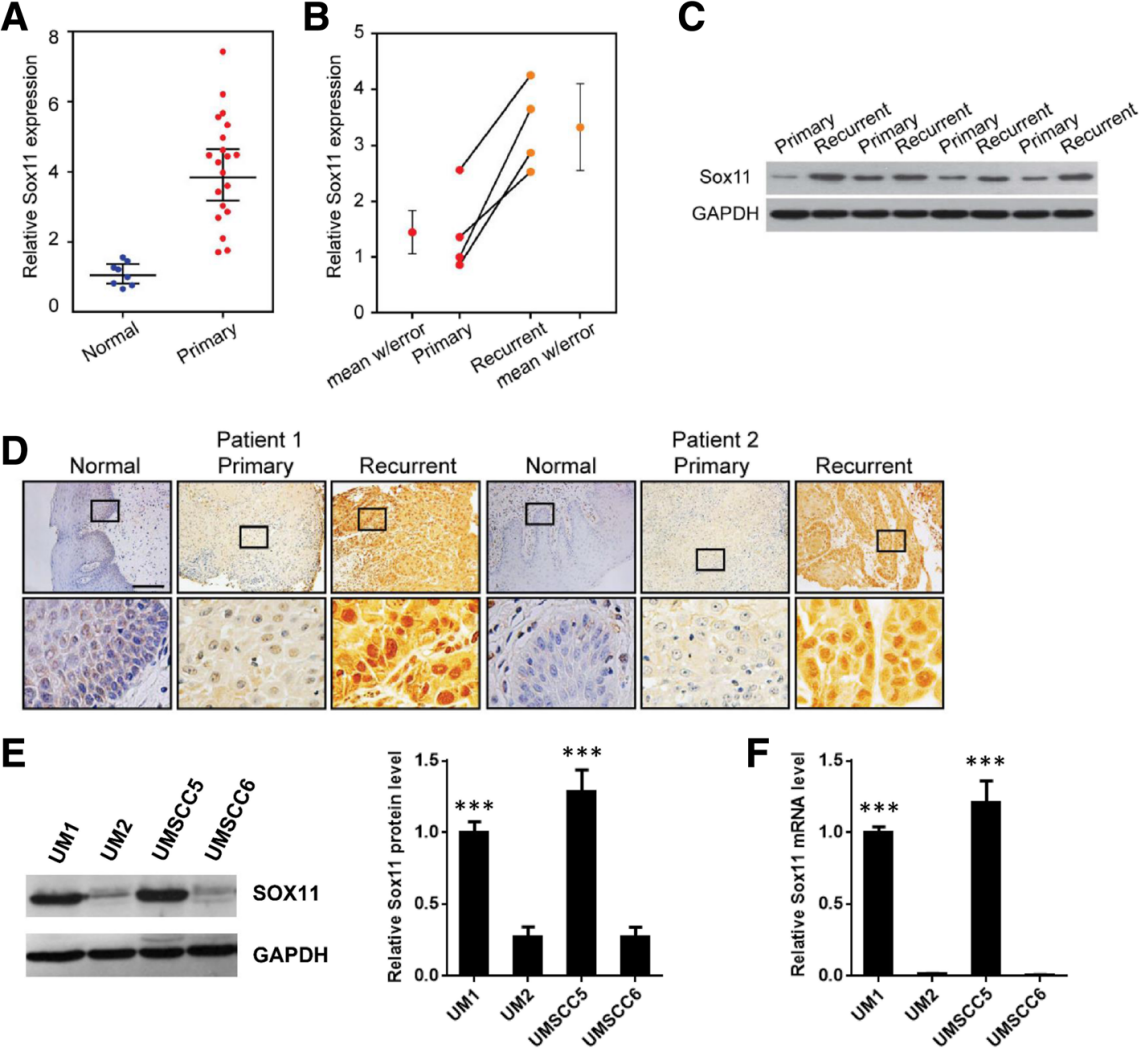
Upregulation of Sox11 in primary and recurrent HNSCC tissues and highly invasive HNSCC cells. (**a**) Relative Sox11 gene expression in 20 primary HNSCC (oral cancer) tissues analyzed by qRT-PCR. ***, *P* < 0.001 compared with eight normal tissues. (**b**) Relative Sox11 gene expression in four paired primary and recurrent HNSCC (oral cancer, same patients) tissues analyzed by qRT-PCR. ***, *P* < 0.001 compared with primary tissues. (**c**) SOX11 expression in four paired primary and recurrent HNSCC (oral cancer) tissues analyzed by western blotting. (**d**) IHC analysis of SOX11 expression in paired normal, primary and recurrent HNSCC (oral cancer) tissues. (**e**) Western blot analysis of SOX11 expression in four HNSCC cell lines (UM1, UM2, UMSCC5 and UMSCC6). (**f**) qPCR analysis of Sox11 gene expression in UM1, UM2, UMSCC5 and UMSCC6 cell lines. ***, *P* < 0.001 (UM1/UMSCC5 versus UM2/UMSCC6)

As shown in Fig. 1, qPCR analysis indicated that relative *Sox11* gene expression was significantly higher in primary oral tongue cancer tissues than normal tissues. More importantly, both *Sox11* gene and protein expression was significantly overexpressed in recurrent oral cancer tissues when compared to paired primary oral cancer tissues (Fig. 1B, C and D). In addition, both *Sox11* gene and protein expression levels was significantly upregulated in highly invasive head and neck cancer cell lines UM1 and UMSCC5 when compared to low invasive UM2 and UMSCC6 cell lines (Fig. 1E, F). Four HNSCC cell lines, UM1, UM2, UMSCC5 and UMSCC6, were used in this study for in vitro experiments. UM1 and UMSCC5 cells are highly invasive and migratory whereas UM2 and UMSCC6 cells are low invasive and migratory (data not shown).

### SOX11 promotes HNSCC cell proliferation and chemoresistance

**Fig. 2:**
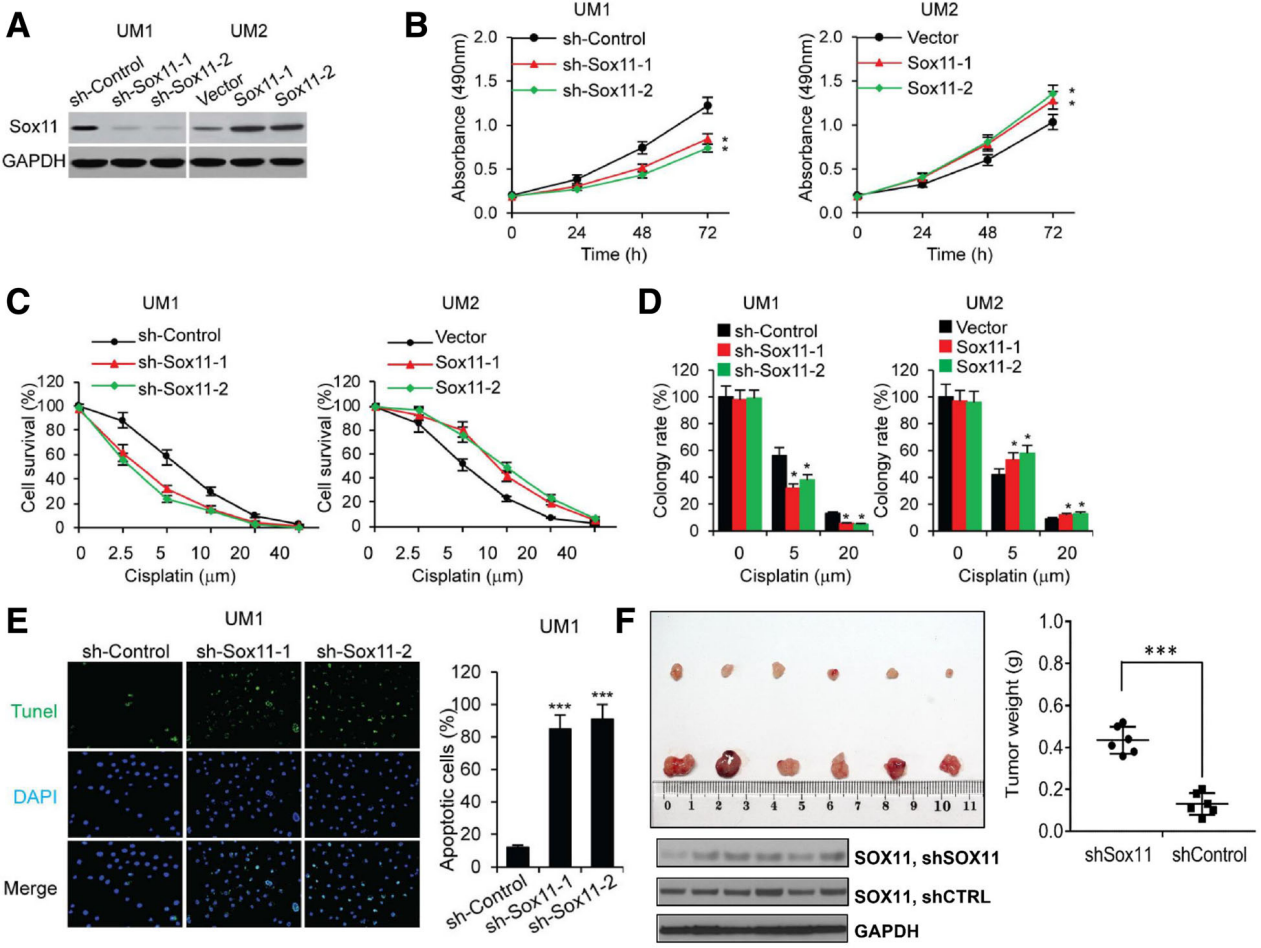
SOX11 promotes oral/head and neck cancer cell proliferation and chemoresistance. (**a**) Western blotting confirmation of SOX11 expression in Sox11-silencing UM1 cells and Sox11-overexpression UM2 cells. (**b**) MTS assays of stable Sox11-knockdown UM1 cells (sh-Sox11–1 and sh-Sox11–2) and Sox11-overexpression UM2 cells (Sox11–1 and Sox11–2). **, *P* < 0.01 compared with control cells. (**c**) Cell survival was examined in Sox11 knockdown (UM1) and overexpression (UM2) cancer cells after treatment with the indicated doses of Cisplatin (0, 2.5, 5, 10, 20 or 40 μM). (**d**) Colony formation rate in Sox11 knockdown (UM1) and overexpression (UM2) cancer cells. *, *P* < 0.05 compared with control cells. (**e**) Tunel staining of Sox11 knockdown cells. ***, *P* < 0.01 compared with control cells. (**f**) Knockdown of Sox11 suppresses xenograft tumor growth in vivo. Stable Sox11 knockdown UM1 cells were subcutaneously injected into nude mice. Four weeks later, Sox11 knockdown UM1 cells had smaller tumors than controls (***, *P* < 0.001). Western blot analysis indicated decreased expression of SOX11 in xenograft tumors derived from UM1 cells with stable Sox11 knockdown

As shown in Fig. 2, knockdown of SOX11 in UM1 cells significantly impaired the cell proliferation and its chemoresistance to Cisplatin. Meanwhile, overexpression of SOX11 in UM2 cells significantly enhanced the cell proliferation and chemoresistance to Cisplatin. Similar results were observed in UMSCC5 or UMSCC6 cells (data not shown). We also found that knockdown of SOX11 in UM1 cells led to significantly increased percentage of apoptotic cells (Fig. 2E). Next, we assessed the potential effect of SOX11 in tumorigenicity using shSox11 xenograft tumors formed in Balb/c nude mice. The weight of the shSox11 xenograft tumors was significantly decreased when compared to the shCTRL xenograft tumors (*n* = 6) (Fig. 2F). As expected, the expression of SOX11 was significantly lower in the shSox11 xenograft tumors than the control tumors.

### SOX11 promotes HNSCC cell migration and invasion

**Fig. 3:**
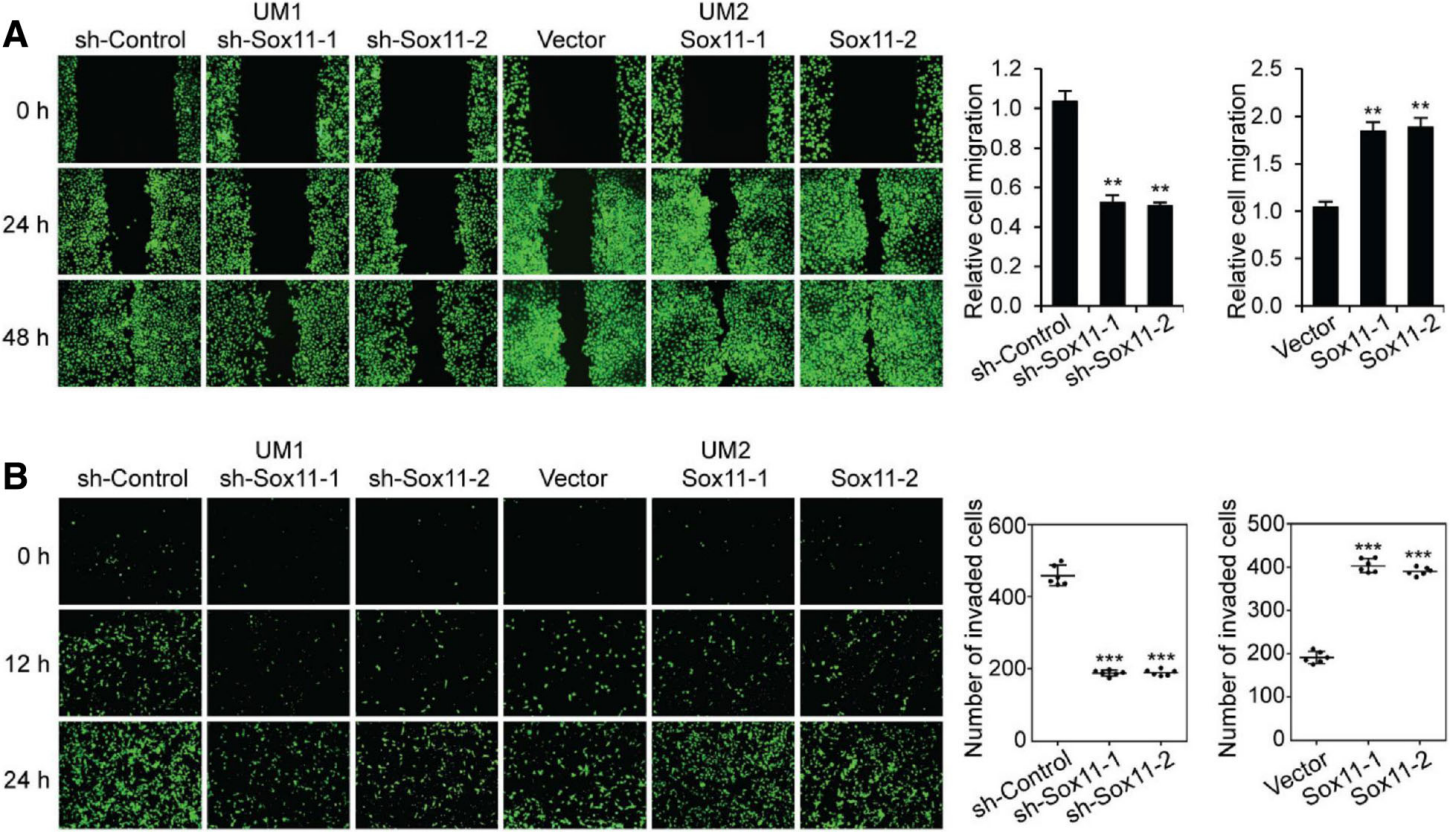
SOX11 promotes head and neck (oral) cancer cell migration and invasion. (**a**) Wound healing cell migration assays of Sox11 knockdown UM1 cells and Sox11 overexpression UM2 cells. **, *P* < 0.01 compared with control cells. (**b**) Transwell cell invasion assays in Sox11 knockdown and overexpression oral cancer cells. ***, *P* < 0.001 compared with control cells

Wound healing assays indicated that knockdown of SOX11 in UM1 cells significantly impaired the cell migration whereas overexpression of SOX11 in UM2 cells significantly enhanced the cell migration. Meanwhile, transwell invasion assays suggested that knockdown of SOX11 in UM1 cells significantly inhibited the cell invasion while overexpression of SOX11 in UM2 cells significantly enhanced the cell invasion (Fig. 3).

### Quantitative proteome analysis

**Fig. 4:**
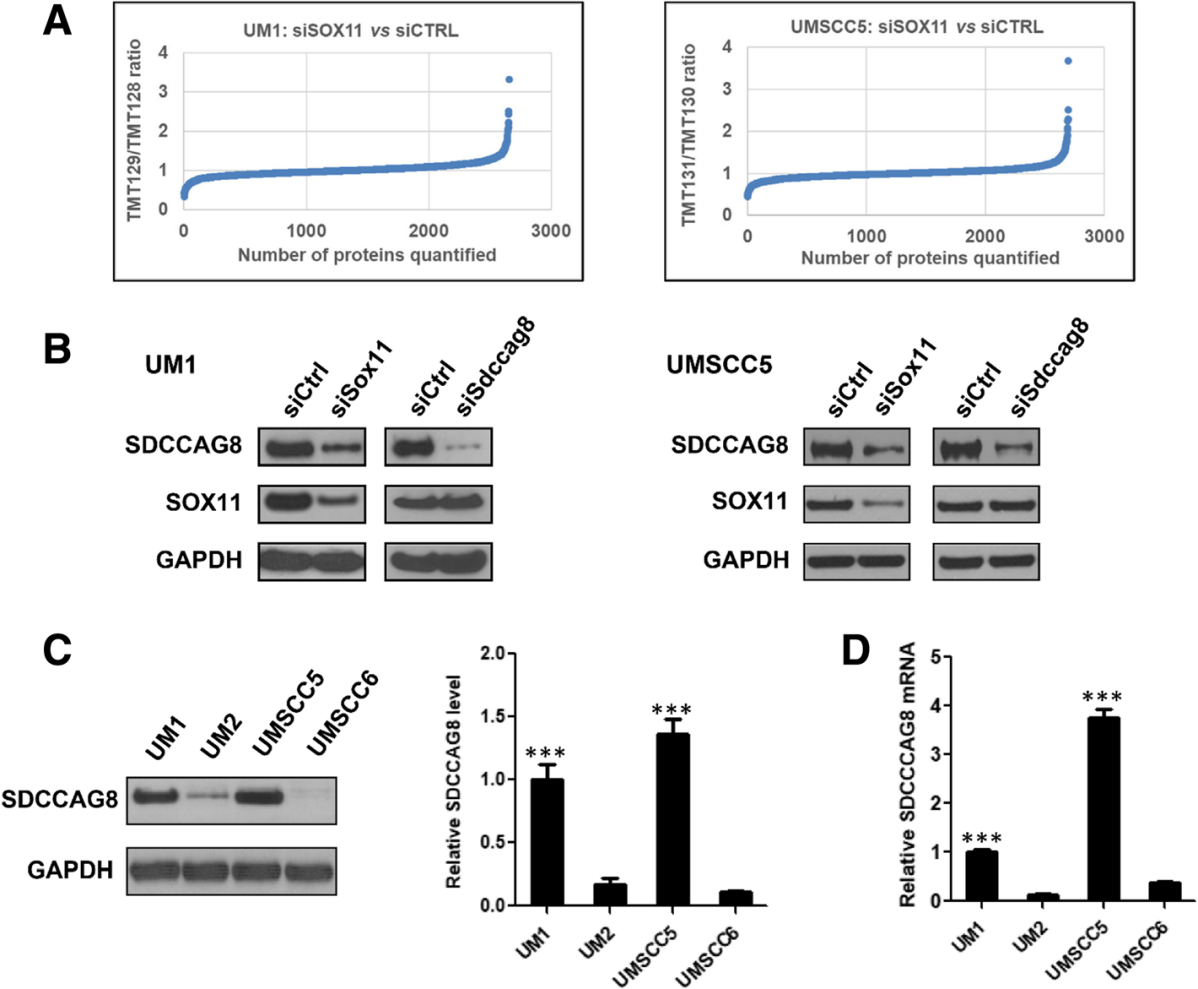
Knockdown of SOX11 in head and neck cancer cells inhibits the expression of SDCCAG8 tumor antigen. (**a**) Quantitative proteomic analysis of UM1 or UMSCC5 cells transfected with siCTRL (UM1: TMT128; UMSCC5: TMT130) or siSox11 (UM1: TMT129; UMSCC5: TMT131). The TMT129:TMT128 ratios (y-axis) represent the relative levels of cellular proteins between UM1-siCTRL and UM1-siSox11 whereas the TMT131:TMT130 ratios represent the relative levels of cellular proteins between UMSCC5-siCTRL and UMSCC5-siSox11. (**b**) Western blot analysis of SOX11 and SDCCAG8 in UM1 or UMSCC5 cells transfected with siSox11 or siSdccag8. (**c**) Western blot analysis of SDCCAG8 in UM1, UM2, UMSCC5 and UMSCC6 cells (*n* = 3, ***, *P* < 0.001). (**d**) qPCR analysis of Sdccag8 gene expression in UM1, UM2, UMSCC5 and UMSCC6 cells (n = 3, ***, *P* < 0.001)

Quantitative proteomics analysis was performed to compare the global protein expression between siSox11- and siCtrl-transfected UM1 cells as well as between siSox11-and siCtrl-transfected UMSCC5 cells (Fig. 4A). In total, more than 2700 proteins were quantified by using the TMT (tandem mass tag) stable isotope labeling and LC-MS/MS (Additional file 1: Table S2). One of the down-regulated proteins in both UM1 and UMSCC5 cells following siSox11 knockdown was identified as SDCCAG8, a putative tumor antigen. Western blot analysis further confirmed siSox11 knockdown significantly inhibited SDCCAG8 expression in both UM1 and UMSCC5 cells. However, knockdown of SDCCAG8 expression did not significantly alter the expression of SOX11 in the cancer cells (Fig. 4B).

### SOX11 regulates the expression of SDCCAG8 in HNSCC cells

Since quantitative proteome analysis indicated that SDCCAG8 might be a potential downstream target gene of SOX11 in HNSCC cells, we then compared *Sdccag8* gene and protein expression among UM1, UM2, UMSCC5 and UMSCC6 cells. As shown in Fig. 4C and D, both *Sdccag8* mRNA and protein expression levels were significantly up-regulated in highly invasive UM1 and UMSCC5 cells when compared to low invasive UM2 and UMSCC6 cells, suggesting that the expression levels of Sox11 and Sdccag8 are highly correlated in HNSCC cells.

**Fig. 5:**
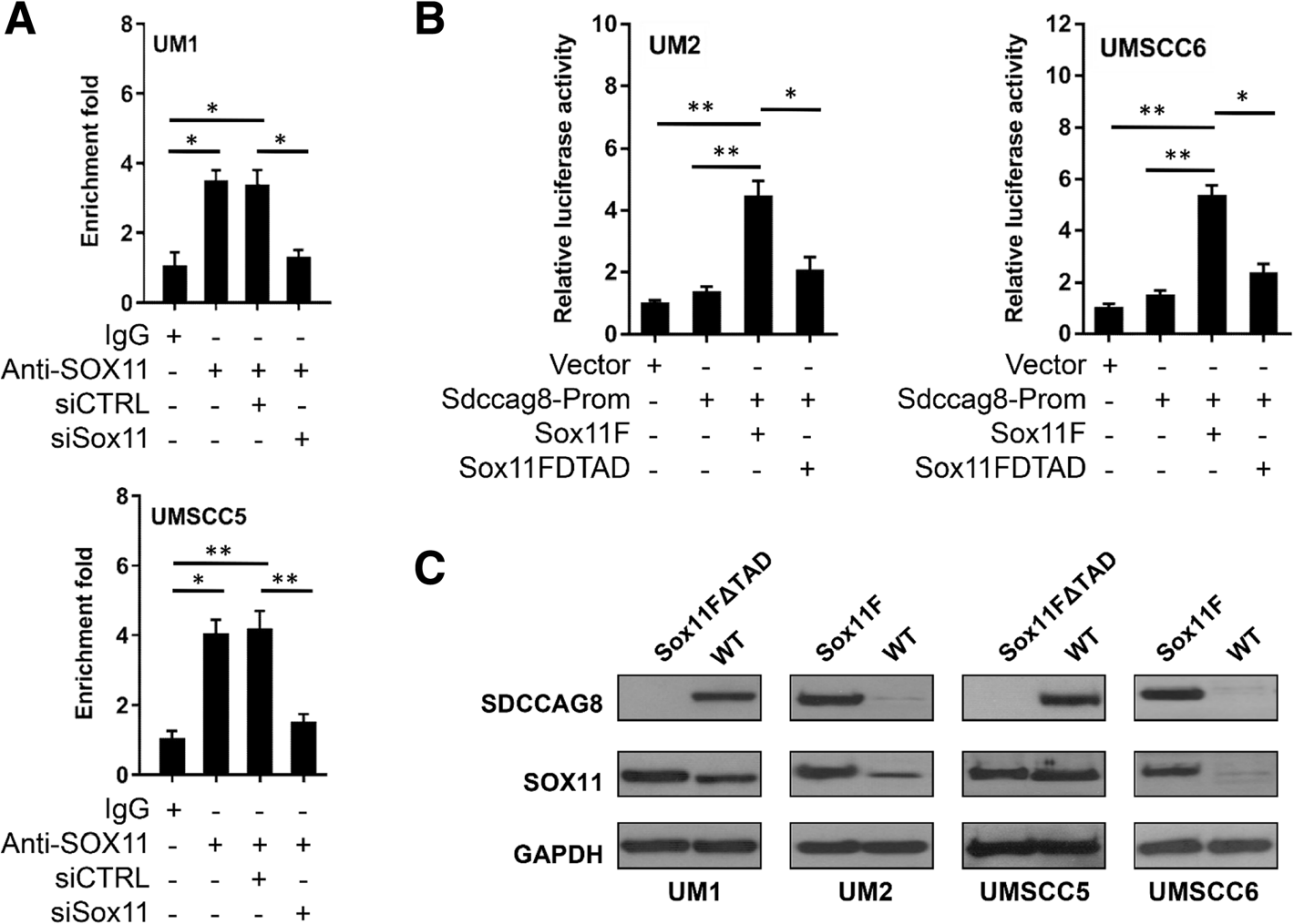
SOX11 binds to Sdccag8 gene promoter and induces Sdccag8 gene promoter activity. (**a**) ChIP-qPCR analysis of anti-SOX11- or IgG-immunoprecipitated DNA fragments from UM1 or UMSCC5 cells. (**b**) Luciferase reporter assays of UM2 or UMSCC6 cells transfected with empty promoter reporter vector, Sdccag8 gene promoter reporter (Sdccag8-Prom), Sdccag8-Prom and Sox11F, or Sdccag8-Prom and Sox11FΔTAD, respectively. Data are measured in triplicates. *, *P* < 0.05; **, *P* < 0.01; ***, *P* < 0.001. (**c**) Western blot analysis of SOX11 and SDCCAG8 in UM1/UMSCC5 cells transfected with Sox11FΔTAD (mutant) and UM2/UMSCC6 cells transfected with Sox11F (WT)

To determine if SOX11 binds to *Sdccag8* gene promoter in HNSCC cells, we performed ChIP assays on UM1 and UMSCC5 cells using anti-SOX11 antibody. The DNA enrichment within the immunoprecipitated samples was measured by real-time qPCR and analysis was performed by comparing the data from the anti-SOX11 immunoprecipitated samples against the background signal of the negative control (IgG antibody) to calculate the enrichment fold. As shown in Fig. 5A, qPCR analyses indicated a significantly higher enrichment of DNA fragments of *Sdccag8* gene promoter in the anti-SOX11-immunoprecipitated UM1 samples than the IgG-immunoprecipitated UM1 samples. The ChIP assays of UMSCC5 cells also showed a significantly higher enrichment of DNA fragments of *Sdccag8* gene promoter. Meanwhile, siRNA knockdown of Sox11 significantly inhibited the enrichment of DNA fragments of *Sdccag8* gene promoter in both UM1 and UMSCC5 cells (Fig. 5A).

To further verify if SOX11 regulates the expression of SDCCAG8 in HNSCC cells, luciferase reporter assays were performed on UM2 and UMSCC6 cells using *Sdccag8* gene promoter reporter plasmid, Sox11 plasmid (**Sox11F**) and a mutant lacking the transactivation domain (**Sox11FΔTAD**). Successful transformation of Sox11 plasmids was confirmed with restriction enzymes and agarose gel electrophoresis of DNA fragment. Figure 5B shows the luciferase assay results of UM2 and UMSCC6 cells transfected with empty promoter reporter vector (200 ng), *Sdccag8* gene promoter reporter (Sdccag8-Prom, 200 ng), Sdccag8-Prom (200 ng) and Sox11F (100 ng), or Sdccag8-Prom (200 ng) and Sox11FΔTAD (100 ng). As expected, when Sdccag8 gene promoter and Sox11F were co-transfected in UM2 or UMSCC6 cells, the luciferase activities were significantly higher than those when the cells were transfected with *Sdccag8* gene promoter alone or empty promoter vector. Meanwhile, when *Sdccag8* gene promoter and Sox11FΔTAD were co-transfected in UM2 and UMSCC6 cells, the luciferase activities were significantly reduced compared to co-transfection of *Sdccag8* gene promoter and SOX11F (wild type). In other words, the luciferase activity of the *Sdccag8* reporter construct, with co-transfection of Sox11FΔTAD which is lack of a transactivation domain, was suppressed in both UM2 and UMSCC6 cells. These results indicated that SOX11, when over-expressed, induced the promoter activity of *Sdccag8* gene in UM2 and UMSCC6 cancer cells. Similar luciferase assay results were also observed in highly invasive UM1 and UMSCC5 cells (data not shown). As presented in Fig. 5C, overexpression of wild-type SOX11 induced the expression of SDCCAG8 in low invasive HNSCC cells (UM2 and UMSCC6) whereas over-expression of mutant SOX11 (Sox11FΔTAD) abolished the expression of SDCCAG8 in highly invasive HNSCC cells (UM1 and UMSCC5).

### SDCCAG8 partially rescues the inhibitory effects of Sox11 knockdown in HNSCC cells

**Fig. 6:**
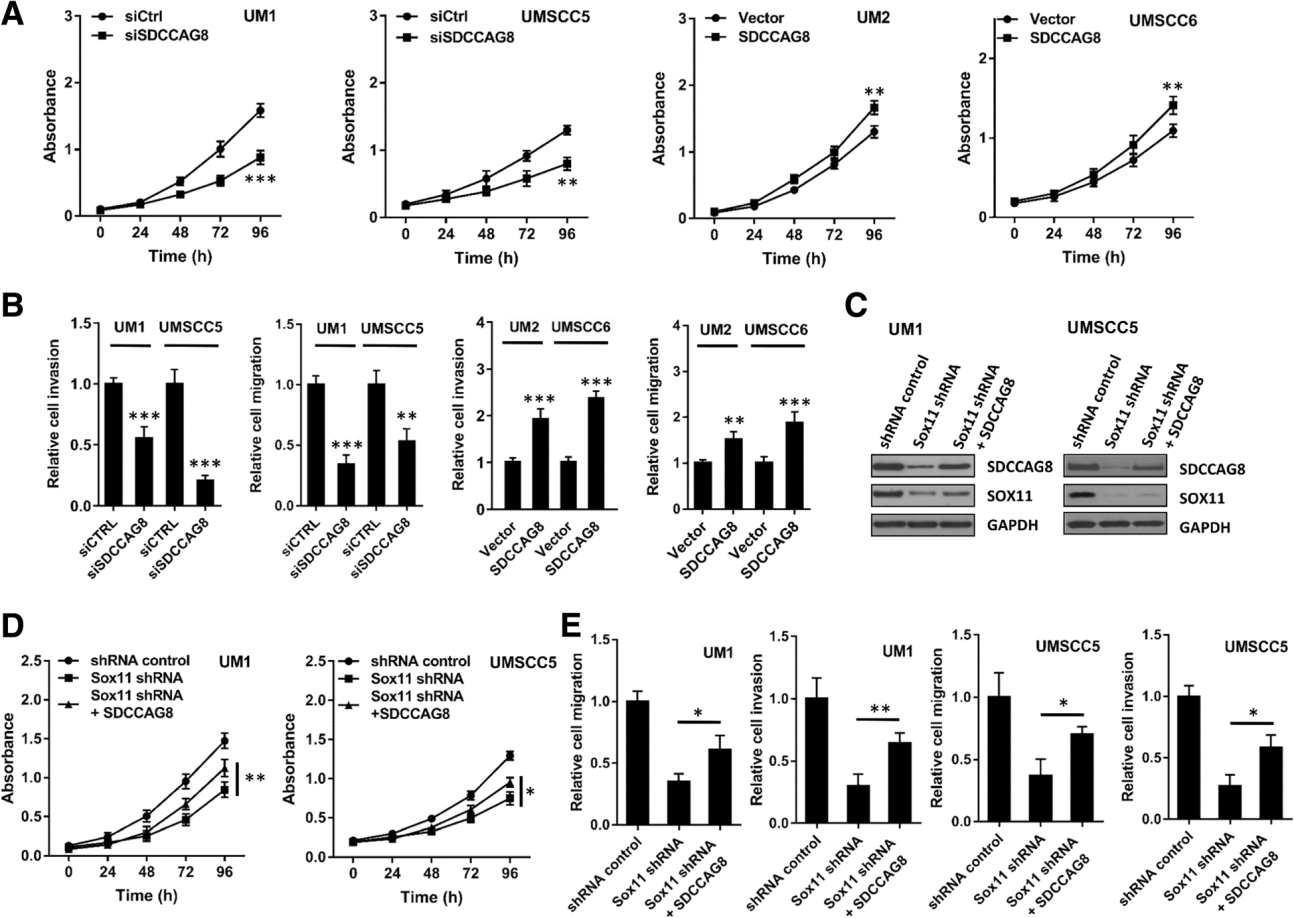
SDCCAG8 promotes HNSCC cell migration/invasion and partially rescues the inhibitory effects of Sox11 knockdown. (**a**) MTS assays of SDCCAG8 knockdown UM1/USCC5 cells or SDCCAG8 overexpression UM2/UMSCC6 cells. (**b**) Invasion and migration assays of SDCCAG8 knockdown UM1/USCC5 cells or SDCCAG8 overexpression UM2/UMSCC6 cells. (**c**) Western blot analysis of SOX11 and SDCCAG8 in UM1 and UMSCC5 cells with SOX11 knockdown or with both SOX11 knockdown and SDCCAG8 overexpression. (**d**) MTS assays of UM1 and UMSCC5 cells with either SOX11 knockdown or with both SOX11 knockdown and SDCCAG8 overexpression. (**e**) Invasion and migration assays of UM1 or UMSCC5 cells with either SOX11 knockdown or with both SOX11 knockdown and SDCCAG8 overexpression. Data are measured in triplicates. *, *P* < 0.05; **, *P* < 0.01; ***, *P* < 0.001

As shown in Fig. 6A and B, silencing of SDCCAG8 expression in high invasive UM1 or UMSCC5 cells significantly inhibited the cell proliferation, migration and invasion. On the other hand, overexpression of SDCCAG8 in low invasive UM2 and UMSCC6 cells significantly enhanced the proliferation, migration and invasion of both cell lines (Fig. 6A and B). For the rescue studies, Western blot analysis confirmed simultaneous knockdown of SOX11 and overexpression of SDCCAG8 in UM1 and UMSCC5 cells. As shown in Fig. 6D & E, the inhibitory effects of Sox11 knockdown on cell proliferation, migration and invasion were partially rescued by SDCCAG8 overexpression in both UM1 and UMSCC5 cells.

## Discussion

*Sox* represents a family of transcription factor containing a highly conserved DNA-binding motif called the HMG-box [34]. According to the similarity within the HMG-box domain, *Sox* genes are divided into 8 subgroups of A~H [34]. The human *Sox11* gene, which belongs to group C, is mapped to chromosome 2 at position p25 and encodes a protein of 441 amino acids [35]. SOX11 has been proven as a strong transcription factor containing a transactivation domain in its carboxyl-terminal [36]. In most mature tissues, the expression of SOX11 is relatively low at normal conditions. However, it plays a curial role in embryonic neuronal development, as well as in the developing limbs, face, and kidneys [37]. Previous studies have clearly demonstrated that SOX11 is indispensable for the development of sensory neurons, sympathetic neurons, and spinal cord in addition to many non-neural tissues [7]. Neurogenesis requires neural progenitor cell (NPC) proliferation, neuronal migration, and differentiation. During embryonic development, neurons are generated in specific areas of the developing neuroepithelium and migrate to their appropriate positions. In mice, SOX11 is detected in NPCs throughout the developing neuroepithelium as early as E8.5, prior to the onset of neurogenesis [36, 37]. During later stages of brain development, SOX11 is continuously expressed in NPCs and some differentiated neurons, most notably in the forebrain [36]. These findings suggest that SOX11 promotes the migration of neurons in the developing neuroepithelium during neurogenesis. Recently, SOX11 was found to promote the migration and differentiation of mesenchymal stem cells (MSCs), and MSCs stably expressing SOX11 migrate to the fracture site, initiate callus ossification and improve bone fracture healing [30]. SOX11 was also found to be involved in epithelial-mesenchymal transition (EMT) [38]. The selective inhibition of the normally up-regulated SOX11 in EMT caused changes in the expression pattern of the EMT markers and slowed down the transition.

Enlightened by the fact that SOX11 promotes the migration of neurons and MSCs, we have investigated if SOX11 promotes proliferation, migration and invasion of HNSCC cells. Our studies have shown that SOX11 is significantly over-expressed in primary HNSCC tissues (versus adjacent normal tissues) and even more up-regulated in recurrent HNSCC tissues than paired primary HNSCC tissues. In fact, previous studies have found that SOX11 promotes tumor progression in MCL [8–15, 39], melanoma and breast carcinoma [18, 19]. SOX11 regulates breast cancer cell proliferation/survival and its over-expression significantly correlates with low survival of breast cancer patients. In addition, SOX11 is over-expressed in small cell lung cancer [20] although contradicting results have also been reported in ovarian cancer, mantle cell lymphoma and glioma [22, 23, 25–27]. Another Sox Group C member, SOX4, shares a similar function to SOX11 in human cancers. The gene was found to promote the progression and contribute to the metastatic spread of multiple solid cancer types [20, 40, 41].

Four HNSCC cell lines, UM1, UM2, UMSCC5 and UMSCC6, are used in this study for in vitro experiments. UM1 and UMSCC5 cells are highly invasive and migratory whereas UM2 and UMSCC6 cells are low invasive and migratory (data not shown). In fact, UM1 and UM2 cells were both established from the same tumor of a tongue squamous cell carcinoma patient, harboring same *TP53* gene mutations [42]. A small fraction of UM1 cells possess stem-like cancer cell properties whereas UM2 cells do not [32]. By using these paired HNSCC cell line models, we have found *Sox11* and *Sdccag8* are significantly over-expressed at both mRNA and protein levels in highly invasive HNSCC cells when compared to low invasive HNSCC cells and their expression levels are highly correlated among HNSCC cell lines. In addition, knockdown of SOX11 or SDCCAG8 expression in HNSCC cell lines significantly impairs the cell proliferation, migration, invasion and chemoresistance whereas overexpression of SOX11 or SDCCAG8 significantly enhances the proliferation, migration, invasion and chemoresistance of HNSCC cell lines. These results suggest that SOX11 and SDCCAG8 promote the proliferation, migration and invasion of HNSCC cells.

*Sdccag8* is a protein coding gene that has been identified as a tumor antigen with various tumor associations [43–46]. It is also known as CCCAP (Centrosomal Colon Cancer Autoantigen Protein), NY-CO-8 (human colon cancer antigen), NPHP10 (Nephronophthisis-related Ciliopathies 10), SLSN7 (Senior-Loken syndrome 7), and BBS16 (Bardet-Biedl Syndrome 16). Mutation of *Sdccag8* causes diseases such as nephronophthisis, Bardet-Biedl syndrome, and retinal-renal ciliopathy, and it is also often observed in patients with mental retardation, cognitive impairment, and seizures [31, 47, 48]. There is a common function between SOX11 and SDCCAG8, which is the regulation of neuronal migration [7, 31]. Recent studies have demonstrated that SDCCAG8 was up-regulated in human lung cells with over-expressed MASPIN playing a role in the invasion of cancer cells [44]. Over-expression of SDCCAG8 was also observed in gastric cancer cells of patients with poor survival rates [48], and in diffuse-type gastric cancer cells, which are non-cohesive and poorly differentiated. These cells often metastasize into the peritoneum or lymph nodes [49]. In addition, SDCCAG8 was suggested as a biomarker to classify cervical cancer patients, who can benefit from radiotherapy only treatment, from those who would need both radiotherapy and chemotherapy [45].

Although there has been clear evidence of SDCCAG8 in regulating cellular functions (e.g., cell cycle, mitotic G2-G2/M phases, recruitment of centrosome proteins, etc.) [31, 43, 50], the underlying mechanisms of SDCCAG8 in cancers, particularly in HNSCC, are largely unknown. In our studies, both *Sdccag8* gene and protein expression were significantly up-regulated in highly invasive HNSCC cells versus low invasive HNSCC cells. Similar to the phenotypic changes in highly invasive HNSCC cells caused by SOX11 knockdown, knockdown of SDCCAG8 in highly invasive HNSCC cells significantly impaired the cell proliferation, migration and invasion. On the other hand, overexpression of SDCCAG8 in low invasive HNSCC cells significantly enhanced the cell proliferation, migration and invasion.

Our studies also demonstrated that *Sdccag8* is a downstream target gene of SOX11 in HNSCC cells. First, quantitative proteome analysis followed by qPCR and Western blotting validation indicated that both *Sdccag8* gene and protein expression was down-regulated when SOX11 expression was knocked down in highly invasive UM1 and UMSCC5 cells. Secondly, the ChIP assays demonstrated that SOX11 bound to *Sdccag8* gene promoter in HNSCC cells. Thirdly, the luciferase reporter assays demonstrated that SOX11 induced *Sdccag8* gene promoter activity in low invasive HNSCC cells when both *Sdccag8* gene promoter construct and wild-type SOX11 (but not mutant SOX11) are co-transfected. Fourthly, overexpression of wild-type SOX11 in low invasive HNSCC cells (UM2 and UMSCC6) induced the expression of SDCCAG8 whereas over-expression of mutant SOX11 in highly invasive HNSCC cells (UM1 and UMSCC5) abolished the expression of SDCCAG8. Lastly, the inhibitory effects of SOX11 knockdown on HNSCC cell invasion, migration and proliferation were partially rescued by over-expression of SDCCAG8. Taken together, our findings suggest that SOX11 regulates SDCCAG8 expression in HNSCC cells.

## Conclusion

In summary, we have demonstrated that SOX11 promotes head and neck cancer progression via the regulation of SDCCAG8. Both molecules may serve as potential prognostic biomarkers or targets for therapeutic intervention in HNSCC. Our study adds new insights regarding the role of SOX11 in human cancer. It again suggests that the function of SOX11 in tumor progression may be tissue specific and cell context dependent. Although SOX11 promotes cancer progression in HNSCC, the regulatory role of SOX11 in early oral/head and neck carcinogenesis remains inconclusive. Further studies are warranted to investigate if SOX11 is essential for early development of oral/head and neck cancer.

## Additional file

Additional file 1: **Table S1.** Primers used for qPCR analysis in this study. **Table S2.** Quantitative proteomic analysis of UM1 cells (TMT129: TMT128) or UMSCC5 cells (TMT131:TMT130) transfected with siSOX11 or siCTRL (UM1-siSOX11: UM1-siCTRL = TMT129: TMT128; UMSCC5-siSOX11: UMSCC5-siCTRL = TMT131: TMT130). (PDF 886 kb)

## Abbreviations

ChIP: Chromatin immunoprecipitation
HNSCC: Head and neck squamous cell carcinoma
LC-MS/MS: Liquid chromatography with tandem mass spectrometry
SDCCAG8: Serologically defined colon cancer antigen 8
TMT: Tandem mass tag
