# Supplementary material for: Sox11 promotes head and neck cancer progression via the regulation of SDCCAG8
Source: J Exp Clin Cancer Res. 2019 Mar 29;38:138. doi: 10.1186/s13046-019-1146-7 (PMC6440126; doi:10.1186/s13046-019-1146-7)
Supplement: Supplementary file 1 — Table S1. Primers used for qPCR analysis in this study. Table S2. Quantitative proteomic analysis of UM1 cells (TMT129: TMT128) or UMSCC5 cells (TMT131:TMT130) transfected with siSOX11 or siCTRL (UM1-siSOX11: UM1-siCTRL = TMT129: TMT128; UMSCC5-siSOX11: UMSCC5-siCTRL = TMT131: TMT130). (PDF 886 kb) [file 13046_2019_1146_MOESM1_ESM.pdf]

Supplementary Table 1. Primers used for qPCR analysis in this study.

Primers	Forward	Reverse
Sdccag8	CCATCGAAAGACTGGTTAAAGAA	CTTTTTCAAGTCGCTCCGCC
Sox11	CCAGGACAGAACCACCTGAT	CCCCACAAACCACTCAGACT
Beta-actin	AGCGAGCATCCCCCAAAGTT	GGGCACGAAGGCTCATCATT
Sdccag8-ChIP	TCCAAGTGAAGGTCCGGTTG	CTAGCTTGGACGGTTTGGCT

Supplementary Table 2. Quantitative proteomic analysis of UM1 cells (TMT129: TMT128) or UMSCC5 cells (TMT131:TMT130) transfected with siSOX11 or siCTRL (UM1-siSOX11 : UM1-siCTRL = TMT129 : TMT128; UMSCC5-siSOX11 : UMSCC5-siCTRL = TMT131 : TMT130).

Protein name	MW [kDa]	calc. pI	129/128	131/130
Uncharacterized protein C18orf58	44.61	4.87	0.9139	0.4476
Similar to hCG1641913	54.98	6.74	1.3206	0.4550
C-type lectin domain family 4 member F	65.48	6.70	1.7535	0.5199
GS homeobox 2	32.04	8.97		0.5208
Tripartite motif-containing protein 75	53.64	7.66	0.4156	0.5301
5 kDa protein	5.07	9.89		0.5333
Isoform Short of Kelch repeat and BTB domain-containing protein 10	66.82	5.43	0.7279	0.5462
35 kDa SR repressor protein	30.59	11.69		0.5540
cDNA FLJ77465, highly similar to Homo sapiens piggyBac transposable element derived 3, mRNA	119.38	6.58	0.6810	0.5791
Isoform 4 of Calcipressin-1	13.28	6.06		0.5822
Putative ATP-binding domain-containing protein 3-like protein	30.15	8.68		0.5880
19 kDa protein	19.47	5.39	0.8699	0.5912
Isoform 3 of MAGUK p55 subfamily member 4	29.78	5.82		0.5990
Isoform B of Metabotropic glutamate receptor 8	101.54	8.15	1.0220	0.6026
Isoform 4 of Zinc finger and SCAN domain-containing protein 29	53.04	9.13	0.6935	0.6049
Usher syndrome 1C binding protein 1, isoform CRA_a	41.07	6.13	0.4239	0.6075
Isoform Short of Laminin subunit gamma-2	121.53	6.38	0.6681	0.6094
cDNA FLJ39374 fis, clone PEBLM2008576, highly similar to Homo sapiens TOLLIP protein	23.21	5.15		0.6144
cDNA FLJ50886, highly similar to Aconitate hydratase, mitochondrial	54.43	7.62	0.6057	0.6314
SPOCD1 protein (Fragment)	29.39	5.14	1.1647	0.6378
Enoyl-CoA hydratase, mitochondrial	31.37	8.07	0.7349	0.6484
43 kDa protein	42.67	5.35	1.0730	0.6529
Uncharacterized protein C5orf49	16.98	7.59	0.8244	0.6575
Transcription factor CP2-like protein 1	54.59	7.01		0.6590
diphthamide biosynthesis protein 2 isoform b	27.51	4.70		0.6606
Isoform Gamma of Max-like protein X	33.28	8.06	1.1435	0.6622
Isoform SERCA3C of Sarcoplasmic/endoplasmic reticulum calcium ATPase 3	112.42	5.80		0.6783
L-serine dehydratase	34.68	7.96	0.8166	0.6784
Isoform 1 of A disintegrin and metalloproteinase with thrombospondin motifs 9	216.35	7.77	0.6883	0.6853
Isoform 2 of Coiled-coil-helix-coiled-coil-helix domain-containing protein 8	11.30	6.67	0.9500	0.6924
Isoform 1 of Serine/threonine-protein phosphatase 4 regulatory subunit 4	99.39	7.80	1.1257	0.6932
11 kDa protein	10.74	5.69	0.8498	0.6937
Heat shock 70 kDa protein 4L	94.43	5.88	1.0278	0.6969
Zinc finger protein 616	90.21	9.57	1.2151	0.6971
Ectonucleotide pyrophosphatase/phosphodiesterase family member 3	100.06	6.57	1.3924	0.6991
Isoform 1 of Protein FAM18B2	31.02	9.13	1.4444	0.7002
cDNA FLJ58756, highly similar to Nuclear pore complex protein Nup93	79.83	6.18	0.7536	0.7014
Vacuolar H+ ATPase E1 isoform c	22.69	7.20	0.8676	0.7032
ubiquitin specific protease 14 isoform b	52.35	5.78		0.7033
constitutive androstane receptor isoform 15	30.24	9.80	0.7566	0.7037
Isoform 2 of Protein FAM102A	40.28	8.82	1.1771	0.7082
WD repeat-containing protein 46	68.00	9.67	0.6938	0.7083
Isoform 4 of Tripartite motif-containing protein 7	23.68	6.93	0.8503	0.7117
Putative uncharacterized protein EXOC6B	79.32	6.27	1.1515	0.7141
Isoform 1 of SH3 domain and tetratricopeptide repeats-containing protein 2	144.68	6.37	0.8736	0.7174
Vascular endothelial growth factor receptor 3	145.51	6.29	1.0766	0.7181
Isoform 2 of Uncharacterized protein C4orf8	135.88	6.51	1.0290	0.7196
Frizzled-6 protein	79.24	7.96	1.2939	0.7199
8 kDa protein	7.69	7.93	1.1004	0.7219
Isoform 2 of Protein midA homolog, mitochondrial	38.00	8.60		0.7233
Isoform 1 of Cingulin-like protein 1	148.95	5.67	0.9570	0.7257
Tight junction protein ZO-3	102.74	6.54	0.6601	0.7267

Isoform 1 of Electron transfer flavoprotein subunit beta	27.83	8.10	1.1337	0.7270
TUBB6 protein	50.06	4.89		0.7290
Similar to homeodomain protein Sax2	56.46	10.07	1.2897	0.7322
cDNA FLJ50214, highly similar to Follistatin-related protein 1	31.21	5.59	1.1941	0.7324
Isoform 2 of Epithelial-stromal interaction protein 1	35.43	9.85	0.8581	0.7326
cDNA FLJ53497, highly similar to Meckel syndrome type 1 protein	47.66	7.14	1.0243	0.7340
Vacuolar protein sorting-associated protein 52 homolog	82.17	5.99	1.0304	0.7345
Serine palmitoyltransferase 2	62.88	7.78	1.1728	0.7349
Isoform 2 of Potassium channel subfamily T member 1	141.91	7.36	0.5620	0.7375
PR domain containing 13	73.93	8.73	0.6842	0.7377
E4F1 protein (Fragment)	6.71	9.44	0.8052	0.7416
hypothetical protein LOC140699 isoform 1	119.23	8.32		0.7425
Isoform 2 of Sodium channel protein type 3 subunit alpha	221.32	5.76	1.1249	0.7438
Protein jagged-1	133.71	6.06	1.2050	0.7445
Inorganic pyrophosphatase	32.64	5.86	0.8520	0.7474
cDNA FLJ40080 fis, clone TESTI2001795, highly similar to Homo sapiens regulator of G-protein signalling 22 (RGS22), mRNA	126.12	8.21	0.8597	0.7475
Isoform 3 of Microtubule-associated protein 2	198.96	4.91	0.8026	0.7482
Isoform 3 of Coiled-coil domain-containing protein 144A	133.79	5.57	1.0634	0.7537
Isoform 9 of Hereditary hemochromatosis protein	18.64	5.69	1.1641	0.7542
G protein-coupled receptor kinase interactor 1 isoform 1	85.39	7.01	1.0081	0.7556
Putative uncharacterized protein ZNF318	231.85	6.40	0.9506	0.7556
12 kDa protein	11.79	5.52	1.0412	0.7556
Vacuolar protein sorting-associated protein 72 homolog	40.57	6.48	0.9504	0.7566
Prefoldin subunit 6	11.55	5.25	0.6955	0.7567
Isoform 3 of Tyrosine-protein phosphatase non-receptor type 13	274.76	6.40	1.2204	0.7569
Isoform 3 of Nostrin	48.92	8.66	0.8962	0.7570
cDNA FLJ50356, highly similar to Nuclear factor from high endothelial venules	25.91	9.26	1.2139	0.7574
92 kDa protein	91.68	7.12	0.9235	0.7579
Isoform 1 of ATP-binding cassette sub-family A member 5	186.39	6.95	0.8922	0.7583
Isoform 2 of Guanine nucleotide exchange factor GEFT	53.14	5.69		0.7584
Isoform 1 of M-phase phosphoprotein 8	97.12	6.06	1.7552	0.7586
Putative uncharacterized protein ADAMTS2	18.66	8.79	1.2156	0.7607
Isoform 1 of Myosin-14	228.56	5.60	0.9696	0.7613
ZNF100 protein	55.57	9.10	1.1361	0.7625
Bromodomain and WD repeat-containing protein 2	136.60	6.92	1.0273	0.7626
NK-tumor recognition protein	165.58	9.99	1.0351	0.7643
Notch-regulated ankyrin repeat-containing protein	12.48	7.39	0.9637	0.7670
Isoform 3 of Zinc finger protein ZFAT	94.38	7.88		0.7671
Isoform 4 of V-type proton ATPase subunit G 3	14.35	9.11	1.1219	0.7694
Gastric-associated differentially-expressed protein YA61P	14.86	7.50	0.9582	0.7697
Putative uncharacterized protein CCDC93	73.00	7.99	0.7151	0.7702
BRF1 homolog, subunit of RNA polymerase III transcription initiation factor IIIB, isoform CRA_c	22.29	8.02	0.6964	0.7725
7 kDa protein	6.52	8.24	0.7137	0.7739
23 kDa protein	22.96	9.82	0.9705	0.7752
Isoform 2 of Protein deltex-4	55.25	9.72	1.3930	0.7758
Serologically defined colon cancer antigen 8	78.10	5.94	0.5890	0.7759
74 kDa protein	74.20	6.35	1.1724	0.7775
Isoform 1 of DNA polymerase kappa	98.75	8.13	1.1712	0.7795
40S ribosomal protein S5	22.86	9.72	0.8835	0.7801
TMF1 protein	122.74	4.96	1.2213	0.7802
Lipopolysaccharide-responsive and beige-like anchor protein	318.96	5.62	1.3643	0.7810
113 kDa protein	113.38	9.58	1.1782	0.7822
Putative uncharacterized protein ENSP00000370103 (Fragment)	16.14	9.61	0.9119	0.7822
Isoform 2 of Zinc finger protein 131	67.26	5.10	1.0968	0.7843
cDNA FLJ53452, highly similar to Histidyl-tRNA synthetase	50.09	5.25	1.3959	0.7855

9 kDa protein	8.72	9.67	0.7533	0.7856
Similar to Splicing factor, arginine/serine-rich, 46kD	60.63	11.17	0.7953	0.7863
Vesicle transport-related protein isoform b	65.02	5.83	0.9109	0.7872
Putative uncharacterized protein LOC284009	16.95	9.69	0.8274	0.7877
Mediator of RNA polymerase II transcription subunit 14	160.50	8.73	0.6460	0.7879
Treacher Collins-Franceschetti syndrome 1 isoform b	144.23	9.04	0.8728	0.7895
Similar to hCG1770601	24.77	8.31	0.8662	0.7896
Isoform 2 of DnaJ homolog subfamily C member 16	53.95	7.03	1.0632	0.7909
Isoform 3 of Dystrophin	425.32	5.92	0.9399	0.7930
CENP-B protein family protein	15.66	8.92	1.4934	0.7936
Hepatopoietin PCn127	28.00	4.13	0.9157	0.7938
HCG1784554, isoform CRA_a	39.12	5.45	1.0792	0.7939
Putative uncharacterized protein ENSP00000365642	75.34	7.94	1.0022	0.7950
Estrogen receptor-related protein	37.29	8.10	0.9887	0.7959
Isoform 4 of Protein spire homolog 2	65.33	8.47	3.3161	0.7963
59 kDa protein	58.50	9.77	1.1549	0.7968
cDNA FLJ60294	105.06	9.16	1.0574	0.7971
Vomeronasal type-1 receptor 5	40.75	9.20	0.8879	0.7973
CALM3 protein	16.50	4.46	1.0991	0.7976
Conserved hypothetical protein	18.06	8.66	0.9447	0.7983
cDNA FLJ50230, highly similar to Ribosome recycling factor, mitochondrial	23.50	9.99	1.3440	0.7988
Similar to BCL2-associated transcription factor 1 isoform 1	79.93	10.30	1.0430	0.7989
1-acyl-sn-glycerol-3-phosphate acyltransferase alpha	31.70	9.38	0.6001	0.8003
Tripeptidyl peptidase II	139.68	6.52	0.7150	0.8012
Isoform 7 of Ceramide kinase-like protein	24.82	9.29	0.8502	0.8024
Isoform 1 of Carboxylesterase 8	63.49	9.31	2.0965	0.8025
Isoform RIN1-delta of Ras and Rab interactor 1	77.45	7.20	1.2201	0.8030
Similar to hCG1642312	58.39	9.70	1.0073	0.8030
Isoform 1 of Squamous cell carcinoma antigen recognized by T-cells 3	109.87	5.57	1.1454	0.8031
Isoform 1 of Uncharacterized protein KIAA0467	278.05	6.06	1.1892	0.8032
p120E4F variant (Fragment)	26.20	8.53	1.0818	0.8033
Transmembrane emp24 domain-containing protein 5	25.99	4.84	0.8721	0.8033
Testis-expressed sequence 15 protein	315.16	6.20	0.8023	0.8034
Selenophosphate synthetase 1	18.48	4.79	1.1222	0.8040
Isoform 1 of Bifunctional heparan sulfate N-deacetylase/N-sulfotransferase 3	100.84	8.06	0.9411	0.8045
Cbp/p300 interacting transactivator with Glu/Asp rich carboxy-terminal domain 1	11.31	4.46	1.0224	0.8047
Putative uncharacterized protein CPNE9	61.70	5.27	1.2027	0.8059
Isoform 5 of Protein transport protein Sec31A	56.70	6.60	0.9835	0.8065
6 kDa protein	6.42	10.36	0.9121	0.8067
cDNA FLJ56450, highly similar to Fructose-bisphosphate aldolase C	48.38	8.07	0.9435	0.8074
Isoform 1 of Myosin-10	228.86	5.54	1.1188	0.8077
Similar to Transcription factor IIIB 90 kDa subunit (TFIIIB90) (hTFIIIB90) (B- related factor 1) (BRF-1) (hBRF) (TATA box-binding protein-associated factor, RNA polymerase III, subunit 2) (TAF3B2). Isoform 2	23.15	9.11	1.0150	0.8087
Serine/threonine-protein kinase OSR1	57.99	6.43	1.1288	0.8090
Putative uncharacterized protein ENSP00000368839	12.92	11.49	1.2969	0.8098
PP11517	22.20	11.77	1.0808	0.8099
Isoform 2 of Melanoma inhibitory activity protein 3	206.76	4.82	1.1260	0.8118
Armadillo repeat-containing X-linked protein 5	62.31	8.73	1.2490	0.8119
cDNA FLJ60076, highly similar to ELAV-like protein 1	38.97	9.45	0.9142	0.8128
Mediator of RNA polymerase II transcription subunit 31	15.79	8.54	0.6228	0.8135
Zinc finger protein 423	144.51	6.89	1.0468	0.8137
cDNA FLJ53447, highly similar to Syntaxin-binding protein 2	62.66	6.51	0.7660	0.8142
cDNA FLJ55352, highly similar to Cutaneous T-cell lymphoma-associated antigen 5	76.83	5.54	1.1681	0.8146
Putative uncharacterized protein DKFZp781I12161	162.93	6.01	1.1635	0.8148
40S ribosomal protein S21	9.11	8.50	0.8970	0.8155
Centromere protein F	367.54	5.07	1.4032	0.8156

Isoform 1 of Calcium-transporting ATPase type 2C member 2	103.12	5.66	1.2013	0.8162
Putative uncharacterized protein MYBL2 (Fragment)	78.56	6.99	0.9153	0.8164
Isoform 1 of Uncharacterized protein C9orf142	21.63	5.48	1.1527	0.8166
cDNA FLJ57204, highly similar to Homo sapiens ancient ubiquitous protein 1 (AUP1), transcript variant 2, mRNA	50.98	9.41	1.0124	0.8167
78 kDa protein	77.53	8.73		0.8178
Putative uncharacterized protein MAST4	283.92	8.62		0.8180
Glutathione synthetase	52.35	5.92	0.9934	0.8182
40S ribosomal protein S17	15.54	9.85	0.9744	0.8183
Putative uncharacterized protein CDH8	77.56	4.98	0.8657	0.8184
Putative uncharacterized protein UGCGL2	31.90	9.16	1.0850	0.8190
Zinc finger protein 605	74.33	9.11	1.0540	0.8193
Isoform 2 of Uncharacterized protein C11orf61	37.98	5.71	1.0987	0.8196
Leucine-rich repeat-containing protein 33	76.32	6.16	1.0373	0.8197
Proteasome subunit beta type-2	22.82	7.02	0.9235	0.8207
Acid sphingomyelinase-like phosphodiesterase 3a	51.23	6.33	0.9379	0.8209
cDNA FLJ75170, highly similar to Homo sapiens synaptotagmin-like 1, mRNA	61.83	5.62	0.9119	0.8209
Similar to Ribosome biogenesis protein BMS1 homolog	28.52	9.01	1.0710	0.8214
Putative uncharacterized protein	28.05	9.07	1.0026	0.8215
Isoform 2 of Splicing factor U2AF 65 kDa subunit	53.09	9.09	0.9474	0.8216
cDNA FLJ54930, highly similar to Homo sapiens Dbf4-related factor 1 (DRF1), transcript variant 2, mRNA	16.70	10.27	1.4358	0.8222
RING finger protein 133	42.27	7.49	1.1606	0.8222
Isoform 1 of Phostensin	67.90	5.40	0.9789	0.8225
16 kDa protein	16.29	9.10	0.9066	0.8225
Vomeronasal type-1 receptor 2	44.45	9.39		0.8231
FLJ00357 protein (Fragment)	69.38	5.99	1.0957	0.8232
PR domain zinc finger protein 12	40.38	8.31	1.0124	0.8236
cDNA FLJ56519, highly similar to Interferon-induced protein 44	46.93	8.90	1.0526	0.8243
19 kDa protein	18.74	10.11	0.9572	0.8249
Isoform 2 of Pyruvate dehydrogenase E1 component subunit beta, mitochondrial	37.18	5.90	1.0499	0.8255
EBPL protein	8.96	5.29	1.2617	0.8261
86 kDa protein	86.28	6.99	0.9722	0.8266
Isoform 1 of BTB/POZ domain-containing protein 12	199.89	6.06	1.2286	0.8268
Speckle-type POZ protein-like	44.62	7.28	0.8864	0.8273
Peptidyl-prolyl cis-trans isomerase, mitochondrial	22.03	9.38	0.7090	0.8285
Orexin	13.35	10.77	0.9568	0.8287
Hypoxanthine-guanine phosphoribosyltransferase	24.56	6.68	1.0387	0.8300
Isoform 1 of Phosphatidylinositide phosphatase SAC2	128.33	7.02	1.2284	0.8303
C-type mannose receptor 2	166.55	5.82	0.5318	0.8307
Serine/threonine-protein kinase SBK1	46.22	9.10	0.6864	0.8310
Isoform 2 of Structural maintenance of chromosomes protein 4	140.19	6.80	0.8189	0.8311
Isoform 3 of WD repeat and FYVE domain-containing protein 3	384.58	6.96	0.9066	0.8327
MHC class I antigen	36.28	5.47	1.3569	0.8327
Selenoprotein V	36.73	9.51	0.9583	0.8331
cDNA FLJ57806, highly similar to Sushi repeat-containing protein SRPX	45.09	8.73	0.9665	0.8335
Cadherin-20	88.94	4.70	1.7743	0.8337
cDNA FLJ53031, highly similar to Protein DEK	39.48	8.51	1.3140	0.8341
Isoform 2 of Transducin-like enhancer protein 3	82.17	7.20	0.8911	0.8352
cDNA FLJ90097 fis, clone HEMBA1005929, weakly similar to H.sapiens serine/threonine protein kinase EMK	47.02	8.38	0.9782	0.8356
dolichyl-diphosphooligosaccharide-protein glycosyltransferase precursor	50.77	6.55	0.9904	0.8357
SEC14-like 2 (S. cerevisiae), isoform CRA_c	37.43	7.84	0.9626	0.8358
Isoform 5 of C2 domain-containing protein 3	260.19	7.09	0.8948	0.8377
Zinc finger protein 469	409.95	7.72	0.7598	0.8381
Isoform 2 of Transmembrane protein 63B	56.27	8.31	1.2109	0.8383
Isoform 6 of WD repeat-containing protein 90	53.63	9.04	1.4219	0.8385

Chromosome X open reading frame 59	18.18	8.59	1.0589	0.8386
Isoform 1 of Uncharacterized protein C1orf87	62.00	8.65	1.1325	0.8391
19 kDa protein	19.20	10.01	0.8253	0.8395
cDNA FLJ56806, highly similar to Sentrin-specific protease 5	81.26	9.22	0.8064	0.8402
Isoform 3 of Membrane-spanning 4-domains subfamily A member 14	64.41	5.29	1.1139	0.8402
Platelet-activating factor acetylhydrolase IB subunit beta	25.55	5.92	1.3152	0.8406
Isoform 1 of Paraplegin	88.18	8.69	1.0067	0.8408
cDNA FLJ50299, highly similar to Serine/threonine-protein kinase 35	17.85	9.98	1.1671	0.8408
Conserved hypothetical protein	7.65	9.48	1.0371	0.8417
Occludin/ELL domain-containing protein 1	29.39	9.99	1.0458	0.8420
Isoform 1 of Protein phosphatase 1 regulatory subunit 3A	125.76	5.02	0.9953	0.8429
Isoform 2 of Pleckstrin homology domain-containing family G member 3	80.96	5.49	0.9516	0.8442
E3 ubiquitin-protein ligase MIB1	110.07	6.92	1.0440	0.8445
Similar to DNA-binding protein	18.83	9.00	1.1592	0.8451
Isoform 2 of Putative Polycomb group protein ASXL3	209.55	5.67	0.8968	0.8464
102 kDa protein	102.22	8.34	1.1954	0.8482
UPF0389 protein FAM162B	17.67	10.77	0.8143	0.8482
Isoform 2 of Tripartite motif-containing protein 67	78.02	7.58	1.1253	0.8488
10 kDa protein	10.25	8.68	0.8229	0.8489
Lysosome-associated membrane glycoprotein 3	44.32	8.41	0.9768	0.8494
cDNA FLJ54297, highly similar to Cellular tumor antigen p53	41.21	7.09	1.0443	0.8496
12 kDa protein	12.45	6.48	0.9340	0.8496
F-box only protein 47	51.96	8.44	0.8777	0.8497
Uncharacterized protein C14orf53	25.29	7.18	1.0005	0.8510
22 kDa protein	22.47	7.44	1.0398	0.8515
Isoform 1 of Dynein heavy chain 3, axonemal	470.47	6.43	0.9478	0.8519
Isoform 2 of Anoctamin-8	124.07	5.86	0.8658	0.8522
Conserved hypothetical protein	25.37	8.76	1.1185	0.8522
cDNA FLJ55159	25.66	10.07	1.0478	0.8531
Putative uncharacterized protein unknown	43.79	6.20	0.8829	0.8535
Isoform 2 of Helicase-like transcription factor	99.86	8.69	1.0710	0.8536
Conserved hypothetical protein	10.01	6.51	0.9116	0.8539
androgen receptor isoform 2	44.61	8.51	1.1013	0.8545
lemur tyrosine kinase 3	157.00	4.91	1.1723	0.8545
Isoform 2 of UPF0493 protein KIAA1632	281.11	6.38	1.2492	0.8549
Cytochrome P450 4F22	61.92	8.73	1.1703	0.8552
Isoform Alpha-1 of Nuclear receptor ROR-alpha	58.94	6.74	1.3795	0.8555
27 kDa protein	27.48	8.25	1.0472	0.8568
Isoform 2 of Piwi-like protein 2	105.69	9.04	1.0350	0.8568
Isoform 2 of TBC1 domain family member 9B	138.55	5.29	1.0431	0.8571
Isoform 2 of Proteasome activator complex subunit 3	30.87	6.11	0.9684	0.8573
20 kDa protein	20.35	5.33	1.1198	0.8586
Putative uncharacterized protein DKFZp686M09245	61.56	5.85	0.9723	0.8592
Ha-Ras1 proto-oncoprotein variant (Fragment)	4.02	4.60	0.8900	0.8592
Adenylate cyclase type 8	140.03	6.99	1.1727	0.8592
TC4 protein	12.22	9.32	0.9696	0.8596
SWI/SNF-related matrix-associated actin-dependent regulator of chromatin a4 isoform F	181.15	7.99	1.0991	0.8599
cDNA FLJ50042	87.10	7.84	1.0835	0.8604
17 kDa protein	17.01	9.86	0.9494	0.8607
Phosphatidylinositol-4-phosphate 5-kinase type-1 gamma	73.21	5.29	0.8837	0.8612
ADP-ribosylation factor 3	20.59	7.43	1.0572	0.8618
cDNA FLJ59132, highly similar to Caspase-2	33.15	7.21		0.8620
Proteasome subunit beta type-3	22.93	6.55	0.9455	0.8625
cDNA FLJ20187 fis, clone COLF0433	27.81	5.62	0.8462	0.8626
SUMO-activating enzyme subunit 1	38.43	5.30	0.8607	0.8626
UPF0368 protein Cxorf26	26.04	4.79	1.0505	0.8631

Isoform 1 of Serine/threonine-protein kinase tousled-like 2	87.61	8.41	0.9861	0.8631
Katanin p80 WD40-containing subunit B1	72.29	7.56	1.1099	0.8639
Septin 4, isoform CRA_h	21.71	6.29	0.9829	0.8641
Protein of unknown function DUF1725 domain containing protein	10.06	10.01	0.8878	0.8643
cDNA FLJ52877, highly similar to 26S proteasome non-ATPase regulatory subunit 5	51.28	5.44	1.2561	0.8649
Isoform IA of Proto-oncogene tyrosine-protein kinase ABL1	122.80	8.66	0.6207	0.8651
Similar to Fanconi anemia complementation group D2 protein	44.80	4.87	0.8930	0.8651
Protein unc-13 homolog B	180.56	5.99	1.0028	0.8654
High mobility group protein B1	24.88	5.74	0.8973	0.8654
cDNA FLJ38717 fis, clone KIDNE2009647	21.58	12.45	1.0406	0.8663
Alpha-mannosidase 2C1	115.76	6.57	0.9663	0.8670
FK506-binding protein 2	15.64	9.13	0.8390	0.8671
Isoform 1 of Uncharacterized protein C14orf145	115.71	6.23	0.8296	0.8672
Isoform 1 of Serine/threonine-protein kinase Nek10	133.15	6.80	1.0100	0.8675
Putative uncharacterized protein LOC440356	13.82	9.11	0.9723	0.8675
GTP-binding protein GUF1 homolog	74.28	8.59	0.9658	0.8677
Putative uncharacterized protein DKFZp686B20267	68.29	6.80	0.8822	0.8683
Armadillo repeat-containing X-linked protein 2	65.64	8.53	1.1414	0.8688
similar to SMC6 structural maintenance of chromosomes 6-like 1	12.62	10.54	0.8393	0.8689
Diazepam binding inhibitor, splice form 1G	16.48	9.38	0.9373	0.8692
Isoform 2 of Dynein heavy chain 9, axonemal	503.05	5.92	0.9846	0.8694
Prostate and testis expressed 4	11.40	8.62	1.0643	0.8695
Mitochondrial import receptor subunit TOM70	67.41	7.12	1.0437	0.8695
Isoform 2 of Inner centromere protein	104.93	9.45	0.9198	0.8699
Synaptonemal complex protein 1	114.12	5.96	1.0787	0.8700
Chloride intracellular channel protein 1	26.91	5.17	0.9977	0.8702
NES protein	175.82	4.41	0.5887	0.8703
Transforming growth factor beta-3	47.30	8.03	0.8206	0.8705
Isoform 5 of Homeobox protein cut-like 1	153.36	5.68	1.1791	0.8709
cDNA FLJ53444, highly similar to Tyrosine-protein phosphatase non-receptor type 23	100.65	6.58	1.3611	0.8709
Ezrin	69.37	6.27	1.0276	0.8713
LOC650392 protein (Fragment)	4.44	12.37	0.9358	0.8713
Integrin beta-1-binding protein 2	38.36	5.16	0.9383	0.8716
DNA topoisomerase 1	90.67	9.31	0.8759	0.8717
12 kDa protein	11.98	8.44	1.2279	0.8720
70 kDa protein	70.00	4.79	1.1389	0.8720
tRNA-intron nuclease 2 isoform 3	47.09	8.69	0.9076	0.8724
Transcriptional adaptor 2-like isoform b	36.03	5.96	1.0758	0.8730
Solute carrier organic anion transporter family member 1B3	77.35	8.76	1.0364	0.8734
cDNA, FLJ79404, highly similar to Sorting nexin-19	29.48	5.08	1.2089	0.8735
Glutathione S-transferase	19.73	9.16	0.7380	0.8745
cDNA FLJ52898, highly similar to Nucleobindin-1	51.59	5.31	1.2488	0.8745
5 kDa protein	4.72	7.99	0.8554	0.8745
cDNA FLJ57512	26.49	11.78	1.2766	0.8751
Microsomal glutathione S-transferase 3	16.51	9.38	1.1706	0.8756
cDNA FLJ60588, highly similar to Protein arginine N-methyltransferase 1	32.85	5.72	0.8460	0.8759
Isoform 3 of Inactive L-threonine 3-dehydrogenase, mitochondrial	17.08	9.14	1.0822	0.8759
Isoform 1 of ATP-binding cassette sub-family B member 5	89.77	8.22	0.8315	0.8760
Isoform 2 of Protein FAM71F2	33.45	7.21	0.9959	0.8761
Isoform 2 of Protein unc-45 homolog A	101.61	6.06	1.1596	0.8763
cDNA FLJ53912, highly similar to Zinc finger protein 512	64.55	9.76	0.9354	0.8764
Isoform 2 of Leucine-rich repeat-containing protein 18	29.06	9.76	0.9998	0.8770
Similar to Zinc finger MYM-type protein 5	155.05	6.68	0.7955	0.8771
Similar to Cytochrome P450 2A6	38.33	8.76	0.8541	0.8776
Isoform 2 of Nitric oxide synthase, brain	148.99	7.81	2.0120	0.8777

Isoform 3 of Low molecular weight phosphotyrosine protein phosphatase	14.33	5.58	0.9425	0.8779
Isoform 2 of FAST kinase domain-containing protein 2	74.49	7.99	0.9824	0.8781
cDNA FLJ55833, highly similar to Cytochrome P450 4F8	38.11	7.44	1.0916	0.8781
t-SNARE domain containing 1	56.04	9.01	1.0321	0.8782
Thymosin beta-10	5.02	5.36	0.9803	0.8782
18 kDa protein	17.52	9.19	1.0661	0.8782
similar to melanoma antigen family B, 6	44.22	8.16	1.2154	0.8784
cDNA FLJ55995, weakly similar to Homo sapiens transmembrane and tetratricopeptide repeat containing 1 (TMTC1), mRNA	76.68	8.90	1.3146	0.8786
Olfactory receptor 10J3	36.52	8.29	1.1512	0.8786
Actin-binding Rho-activating protein	43.09	8.54	0.9533	0.8788
Uncharacterized protein C17orf90	15.84	8.22	1.0238	0.8792
FYN-binding protein	85.37	6.48	1.0674	0.8793
Isoform 4 of 1-phosphatidylinositol-4,5-bisphosphate phosphodiesterase eta-1	115.87	7.43	0.8771	0.8794
Putative uncharacterized protein CDH26	98.91	5.99	1.3525	0.8795
Carbonyl reductase [NADPH] 1	30.36	8.32	0.9266	0.8803
Isoform 1 of Cleft lip and palate transmembrane protein 1	76.05	6.30	1.0914	0.8804
Isoform 1 of Phosphofurin acidic cluster sorting protein 2	97.64	6.60	1.0271	0.8804
G-protein coupled receptor EDG-7	40.31	9.48	1.0469	0.8809
BAH and coiled-coil domain-containing protein 1	276.76	8.81	1.2674	0.8810
Isoform B of Zinc finger homeobox protein 3	306.47	6.89	1.1125	0.8815
Isoform 1 of Interleukin-17 receptor B	55.85	8.09	0.9635	0.8816
Isoform 1 of Seprase	87.68	6.65	0.9529	0.8819
BCL2-like 12 isoform 3	36.64	9.03	0.9701	0.8819
Nascent polypeptide-associated complex subunit alpha	23.37	4.56	1.0176	0.8830
Isoform 1 of ELKS/RAB6-interacting/CAST family member 1	128.01	5.97	1.0711	0.8832
Proteasome (Prosome, macropain) 26S subunit, non-ATPase, 10	20.20	5.58	1.0912	0.8832
Isoform 1 of Meiosis inhibitor protein 1	141.07	6.70	1.0187	0.8839
Uncharacterized protein C1orf74	29.54	5.72	0.7860	0.8840
Hypothetical short protein	7.30	9.32	1.0291	0.8842
60S acidic ribosomal protein P1	11.51	4.32	0.9836	0.8845
Proteolipid protein 2	16.68	7.24	0.9737	0.8848
Isoform 1 of Cytosolic carboxypeptidase 3	115.94	8.79	1.0698	0.8853
Lebercilin-like protein	76.46	9.48	0.8421	0.8854
Zinc finger protein 558	45.73	8.95	1.1451	0.8854
Putative uncharacterized protein ENSP00000382756	10.42	9.19	1.1787	0.8854
Zinc finger RNA binding protein	117.62	9.26	1.1649	0.8858
Isoform Sap-mu-0 of Proactivator polypeptide	58.07	5.17	0.9304	0.8860
cDNA FLJ56350, highly similar to HESB-like domain-containing protein 2, mitochondrial	19.28	10.08	0.9752	0.8860
DnaJ homolog subfamily A member 2	45.72	6.48	0.8987	0.8862
Isoform 4 of Potassium voltage-gated channel subfamily H member 2	91.86	8.07	1.0552	0.8863
Flavin reductase	22.11	7.65	1.1097	0.8871
Aspartyl/asparaginyl beta-hydroxylase	85.81	5.01	0.8311	0.8872
Isoform 1 of WD repeat-containing protein 87	332.96	7.28	1.1030	0.8884
GRIP1 associated protein 1	90.79	5.11	0.9398	0.8886
Histone H1.0	20.85	10.84	1.4104	0.8890
similar to hCG20535	56.73	6.29	0.9237	0.8890
cDNA FLJ39246 fis, clone OCBBF2008466	57.19	9.32	0.9220	0.8891
similar to hCG2038359	193.55	9.04	0.9509	0.8892
Isoform 1 of ATP-binding cassette sub-family A member 13	575.87	6.42	1.0293	0.8895
Phosphatidylethanolamine-binding protein 1	21.04	7.53	1.0030	0.8895
RNA-directed DNA polymerase (reverse transcriptase), related domain containing protein	8.93	8.76	1.0896	0.8895
Nucleoprotein TPR	267.13	5.02	0.8958	0.8896
cDNA FLJ60082, weakly similar to Uro-adherence factor A (Fragment)	149.98	5.19	0.9423	0.8903
Protein S100-A16	11.79	6.79	1.0886	0.8904

cDNA FLJ54719, highly similar to Zinc finger protein 7	79.06	8.27	0.8745	0.8906
WD repeat domain 10 isoform 4	129.28	7.27	1.0535	0.8909
13 kDa protein	12.55	4.94	0.9564	0.8909
Isoform 14P-18B of Histone-lysine N-methyltransferase HRX	427.47	9.14	0.6761	0.8912
similar to hCG1790802	24.43	9.85	1.0664	0.8914
Transmembrane protein 132E	107.03	5.90	1.1927	0.8914
Cystatin-B	11.13	7.56	1.3125	0.8916
Leucine-rich repeat transmembrane neuronal protein 1	58.60	7.34	1.0889	0.8918
V_segment translation product	30.11	6.57	1.1485	0.8921
Isoform 2 of Putative thiosulfate sulfurtransferase KAT	10.69	9.54	0.9099	0.8924
Protein S100-A6	10.17	5.48	1.0758	0.8924
15 kDa protein	15.00	8.84	1.0985	0.8925
Isoform 1 of 3-hydroxyacyl-CoA dehydrogenase type-2	26.91	7.78	0.8441	0.8926
Isoform 1 of Dr1-associated corepressor	22.34	5.17	0.9949	0.8927
Isoform 2 of Zinc finger protein 174	26.87	7.06	1.2826	0.8928
11 kDa protein	11.29	6.29	0.8823	0.8929
hypothetical protein	34.09	7.66	0.9248	0.8929
Isoform 2 of Cystic fibrosis transmembrane conductance regulator	161.49	8.66	0.8370	0.8930
cDNA FLJ55071	38.32	9.64	1.1774	0.8931
ADAM DEC1	52.74	7.34	0.8120	0.8940
similar to RIKEN cDNA E130309D14	41.78	11.24	1.1520	0.8940
Rod photoreceptor CNG-channel beta subunit	139.09	4.82	1.3101	0.8940
40S ribosomal protein SA	32.83	4.87	0.9138	0.8942
253 kDa protein	252.54	6.60	0.8618	0.8942
cDNA FLJ55992, highly similar to GTPase, IMAP family member 5	38.60	7.20	1.2020	0.8943
Tubulin beta-2A chain	49.87	4.89	1.0666	0.8943
Coiled-coil and C2 domain containing 2B	22.44	5.83	0.9480	0.8943
Haptoglobin	45.18	6.58	1.1754	0.8949
Suppressor of cytokine signaling 5	61.21	8.57	0.9110	0.8950
Isoform 1 of JNK1-associated membrane protein	36.73	7.93	0.9327	0.8950
cDNA FLJ55263	35.64	6.02	1.0752	0.8953
cDNA FLJ51545, highly similar to Pregnancy-specific beta-1-glycoprotein 9	37.56	7.49	0.9474	0.8954
Nudix-type motif 6 isoform a variant (Fragment)	12.02	6.64	1.0542	0.8955
P2X purinoceptor	49.13	8.24	0.6990	0.8957
hypothetical protein	14.32	4.72	1.1687	0.8957
Putative uncharacterized protein NUDC (Fragment)	32.71	5.99	1.1013	0.8959
Isoform 2 of Glucocorticoid receptor DNA-binding factor 1	170.41	6.64	1.0377	0.8959
Gamma-glutamyl hydrolase	35.94	7.11	1.0760	0.8962
17 kDa protein	16.68	7.33	1.0092	0.8963
37 kDa protein	37.11	8.87	1.1189	0.8965
81 kDa protein	81.27	6.76	1.0149	0.8967
Vasodilator-stimulated phosphoprotein	39.81	8.94	1.1839	0.8971
Protocadherin Fat 1	506.26	5.00	1.0473	0.8971
GTP-binding protein SAR1a	22.35	6.68	1.1551	0.8972
STAG1 protein	140.00	5.45	0.9065	0.8973
Olfactory receptor 1M1	34.82	8.91	0.9195	0.8973
U2 small nuclear RNA auxillary factor 1 isoform c	19.70	9.06	0.9575	0.8977
cDNA FLJ51257, highly similar to NifU-like N-terminal domain-containing protein, mitochondrial	16.54	9.54	1.0641	0.8977
Conserved hypothetical protein	15.73	9.99	1.1015	0.8977
Olfactory receptor 2W3	34.77	8.60	0.9559	0.8977
Tumor protein p53-inducible protein 13	42.21	10.98	1.0504	0.8981
Isoform 3 of Seipin	32.63	9.60	0.9620	0.8981
similar to hCG1820519	13.68	11.27	0.9038	0.8985
Isoform 1 of Protein FAM46A	49.63	5.17	1.5707	0.8987
GRB10 interacting GYF protein 2 isoform c	149.40	5.54	1.5937	0.8989
Centrosome-associated protein 350	350.72	6.33	2.0419	0.8989

similar to hCG1996858	78.75	10.65	0.9698	0.8994
Isoform 2 of ATP-binding cassette sub-family A member 12	256.81	7.49	1.1265	0.8995
Isoform 1 of A disintegrin and metalloproteinase with thrombospondin motifs 7	183.98	6.20	1.0449	0.8996
UPF0556 protein C19orf10	18.78	6.68	1.0151	0.8997
Hypothetical short protein	4.05	5.62	0.9812	0.8997
Isoform 4 of FRAS1-related extracellular matrix protein 1	24.99	5.45	1.0984	0.9001
Isoform 2 of Protein tyrosine kinase 2 beta	111.11	5.91	1.0545	0.9004
Isoform 1 of 5'-nucleotidase domain-containing protein 3	63.38	8.31	1.7181	0.9004
Isoform 2 of Kinesin-like protein KIF23	98.04	8.47	0.8382	0.9007
Arginine/serine-rich splicing factor 6 variant (Fragment)	31.85	10.95	0.8121	0.9009
cDNA FLJ55805, highly similar to Keratin, type II cytoskeletal 4	51.73	6.81	1.0895	0.9013
Isoform AGX2 of UDP-N-acetylhexosamine pyrophosphorylase	58.73	6.33		0.9015
Isoform 7 of Probable histone-lysine N-methyltransferase NSD2	30.18	9.32	0.9737	0.9016
Vanilloid receptor variant TRPV1b	88.01	7.87	1.0454	0.9017
Isoform 4 of Protein NLRC3	111.56	8.24	0.9531	0.9017
Carbohydrate sulfotransferase 12	48.38	9.32	0.7961	0.9017
Isoform 5 of Ceramide kinase-like protein	42.18	8.72	1.1703	0.9019
Isoform 2 of Translocon-associated protein subunit alpha	29.36	4.61	0.8529	0.9020
Isoform 3 of Kinesin-like protein KIF13A	195.95	5.69	0.8656	0.9025
Isoform 1 of Obg-like ATPase 1	44.72	7.81	1.1473	0.9026
Replication protein A3, 14kDa, isoform CRA_a	9.16	4.54	1.1661	0.9027
Isoform 4 of Protein phosphatase 1 regulatory subunit 12A	109.04	5.41	0.7580	0.9029
11 kDa protein	10.64	10.71	0.9492	0.9029
Isoform 3 of Nucleoside diphosphate kinase B	30.12	8.92	0.9533	0.9035
Isoform 3 of Tenascin-X	455.85	5.21	0.9262	0.9035
cDNA FLJ52832, highly similar to Splicing factor, arginine/serine-rich 3	14.19	10.08	0.8984	0.9035
Isoform 6 of Thioredoxin reductase 1, cytoplasmic	67.18	6.77	0.8725	0.9036
Isoform 2 of Centrosome-associated protein CEP250	274.21	5.03	1.0153	0.9036
Isoform 3 of Mucin and cadherin-like protein	53.20	5.08	0.9502	0.9037
Isoform 2 of IQ motif and SEC7 domain-containing protein 1	94.85	7.66	1.1924	0.9037
Isoform DPI of Desmoplakin	331.57	6.81	0.8678	0.9039
cDNA FLJ51276, highly similar to Secretogranin-2	54.24	4.84	0.9140	0.9041
Caspase-14	27.66	5.58	1.0799	0.9043
Isoform 2 of Oxysterol-binding protein-related protein 6	102.23	7.06	0.9546	0.9044
Isoform 2 of PDZ and LIM domain protein 7	46.48	8.34	1.0553	0.9046
cDNA FLJ58992, moderately similar to Annexin A8	13.55	8.16	1.0118	0.9048
Isoform 2 of Regulation of nuclear pre-mRNA domain-containing protein 2	152.49	7.33	0.9214	0.9050
Periodic tryptophan protein 2 homolog	102.39	6.15	0.9963	0.9050
Isoform 3 of Tax1-binding protein 1	69.69	5.41	0.9920	0.9053
Nuclear pore complex protein Nup205	227.78	6.21	0.9030	0.9053
Isoform 1 of SH3 domain-binding protein 1	75.67	6.77	1.1792	0.9054
Conserved hypothetical protein	21.19	8.21	1.0102	0.9056
Isoform 1 of Formin-like protein 1	121.75	5.72	1.0542	0.9056
Myc protein	21.04	11.96	0.9816	0.9057
similar to peptidyl-Pro cis trans isomerase	14.22	7.14	0.9348	0.9060
Isoform 2 of Porphobilinogen deaminase	37.68	7.39	0.9286	0.9061
7-dehydrocholesterol reductase	54.45	8.70	0.8803	0.9066
Isoform 1 of Transcription factor BTF3	22.15	9.38	1.1760	0.9066
Abl2 isoform 1BLCTS	117.87	7.85	0.8906	0.9067
Isoform 1 of N-acetylserotonin O-methyltransferase-like protein	68.81	6.07	1.1237	0.9069
cDNA FLJ54246, highly similar to Homo sapiens BRCA2 and CDKN1A interacting protein (BCCIP), transcript variant C, mRNA	29.78	4.75	0.8881	0.9069
Histone H2A (Fragment)	11.79	10.93	0.9678	0.9069
cDNA FLJ51872, highly similar to Small nuclear ribonucleoprotein Sm D3	13.28	8.91	1.0670	0.9072
DERP12	38.17	6.58	0.9619	0.9077
Isoform 2 of Rap guanine nucleotide exchange factor-like 1	52.31	6.73	1.5031	0.9078
cGMP-inhibited 3',5'-cyclic phosphodiesterase B	124.25	5.91	0.9550	0.9080

cDNA FLJ60266, highly similar to Corticotropin-releasing factor receptor 1	28.10	9.67	0.8958	0.9081
Lamin-B2	67.65	5.35	0.9627	0.9082
Serine/cysteine proteinase inhibitor clade G member 1 splice variant 2 (Fragment)	37.26	8.00	0.9021	0.9084
Isoform 2 of Histone-lysine N-methyltransferase MLL2	531.52	5.78	1.0875	0.9085
Putative uncharacterized protein DKFZp667A016	44.66	5.80	0.9633	0.9086
28 kDa protein	28.25	4.78	1.0726	0.9086
Mitogen-activated protein kinase kinase kinase 6	11.78	6.15	0.9558	0.9088
Putative uncharacterized protein DKFZp313E1411	74.25	9.11	1.0347	0.9090
Isoform 2 of Uncharacterized protein C10orf90	67.06	9.20	0.9083	0.9095
Putative uncharacterized protein TBCB	23.57	7.21	0.9805	0.9100
Isoform Beta-1A of Integrin beta-1	88.36	5.39	1.0538	0.9104
Isoform 2 of Chromodomain-helicase-DNA-binding protein 9	323.85	6.92	0.8581	0.9106
Estradiol 17-beta-dehydrogenase 2	42.76	8.50	0.9341	0.9108
Leucine-rich repeat neuronal protein 1	80.67	6.15	1.1168	0.9111
EF-hand calcium binding domain 3 isoform a	55.95	9.41	1.0526	0.9113
Similar to Hcg1985370	14.88	9.64	0.8946	0.9119
Isoform 1 of 1-phosphatidylinositol-4,5-bisphosphate phosphodiesterase epsilon-1	259.78	6.51	0.9832	0.9120
Isoform 3 of Stromal membrane-associated protein 1	47.83	8.43	0.9495	0.9121
Interleukin-18	22.31	4.67	1.1096	0.9122
p21-activated kinase 3 isoform c	62.87	5.59	0.8813	0.9128
Rho GTPase activating protein 21	217.33	7.80	1.0791	0.9131
WUGSC:H_2G3A.1 protein	330.64	5.95	1.2430	0.9133
hypothetical protein LOC25758	199.36	8.31	1.0336	0.9133
Isoform 3 of Scavenger receptor cysteine-rich type 1 protein M160	155.38	5.69	1.3961	0.9133
Isoform 2 of Kelch-like protein 28	34.09	5.83	1.0320	0.9134
Aspartyl-tRNA synthetase, cytoplasmic	57.10	6.55	1.3456	0.9136
RNA binding motif protein 25	100.12	6.32	1.0574	0.9139
Peptidyl-prolyl cis-trans isomerase B	23.73	9.41	0.9534	0.9139
cDNA FLJ50830, highly similar to Serum albumin	59.54	7.17	2.0434	0.9141
Keratin, type I cytoskeletal 17	48.08	5.02	0.8815	0.9144
Glycerol kinase 2	60.55	5.77	0.8331	0.9147
Putative uncharacterized protein ENSP00000234142	216.42	6.95	1.0230	0.9148
Isoform 2 of Homeodomain-interacting protein kinase 3	131.17	7.58	1.1399	0.9150
cDNA FLJ54770, highly similar to DNA-directed RNA polymerase I-associated factor 53 kDa subunit	39.71	8.53	1.4118	0.9150
Isoform 1 of Coatomer subunit alpha	138.26	7.66	1.1206	0.9154
Ufm1-conjugating enzyme 1	19.45	7.40	1.0636	0.9155
Chromosome 10 open reading frame 27	12.13	6.30	0.9566	0.9157
Isoform 7 of Integrin alpha-6	106.51	7.66	1.1807	0.9160
Cyclin-dependent kinase-like 5	115.47	9.54	1.0534	0.9164
Putative uncharacterized protein PIK3R6 (Fragment)	77.49	7.84	0.8334	0.9164
Isoform 3 of CDP-diacylglycerol--glycerol-3-phosphate 3-phosphatidyltransferase, mitochondrial	37.19	9.89	1.0575	0.9165
58 kDa protein	57.47	8.85	0.9350	0.9166
Isoform 3 of Protein unc-45 homolog B	103.54	7.81	0.9295	0.9166
Isoform 1 of Fer-1-like protein 5	241.81	7.96	1.2772	0.9167
NBEAL1 protein	23.26	5.66	0.8410	0.9170
Isoform 1 of Inversin	117.75	9.35	1.1152	0.9171
Rho guanine nucleotide exchange factor 17	221.53	6.29	0.9217	0.9174
cDNA FLJ45964 fis, clone PLACE7014396	21.54	10.26	1.7357	0.9176
Isoform 2 of UDP-GlcNAc:betaGal beta-1,3-N-acetylglucosaminyltransferase 6	29.47	9.91	0.9409	0.9176
Isoform 1 of Thyroid receptor-interacting protein 13	48.52	6.09	0.7355	0.9177
Rho GDP-dissociation inhibitor 1	23.19	5.11	1.0036	0.9178
CDNA FLJ23801 fis, clone HEP22544	19.48	11.71	1.1119	0.9179
Isoform 1 of BCL-6 corepressor	192.07	6.48	1.0690	0.9180
TSPAN16 protein	17.19	7.74	0.8100	0.9183
84 kDa protein	83.94	6.34	0.9033	0.9183

Peptidyl-prolyl cis-trans isomerase A	18.00	7.81	0.9029	0.9184
31 kDa protein	30.85	8.76	0.9466	0.9186
Alpha-soluble NSF attachment protein	33.21	5.36	1.0998	0.9186
Diacylglycerol kinase iota	116.92	7.77	1.1650	0.9187
34 kDa protein	33.53	5.11	1.3214	0.9187
Putative uncharacterized protein FBXO2	29.52	4.37	0.9291	0.9190
Isoform 4 of Collagen alpha-1(XII) chain	324.37	5.50	0.8737	0.9194
Isoform 3 of Protein virilizer homolog	200.93	4.82	1.1464	0.9196
Cullin-5	96.66	8.41	0.9966	0.9197
Menin	16.14	7.42	1.2654	0.9199
cDNA FLJ54957, highly similar to Transketolase	68.70	7.66	0.8763	0.9201
Isoform 2 of Dehydrogenase/reductase SDR family member 7	32.25	8.87	1.0096	0.9202
Isoform 2 of Probable palmitoyltransferase ZDHHC5	71.91	8.90	0.4619	0.9202
Lupus La protein	46.81	7.12	0.8599	0.9203
cDNA FLJ55237, highly similar to Homo sapiens activating signal cointegrator 1 complex subunit 1 (ASCC1), mRNA	43.55	5.63	1.1154	0.9204
Isoform 1 of Eukaryotic translation initiation factor 5A-1	16.82	5.24	0.9879	0.9205
Similar to Candidate tumor suppressor protein	518.96	5.33	0.8252	0.9205
Putative uncharacterized protein ENSP00000386489	14.25	8.19	0.9566	0.9205
5 kDa protein	4.52	8.66	1.0795	0.9212
21 kDa protein	21.06	8.15	1.1311	0.9213
22 kDa protein	21.80	9.91	0.8915	0.9213
Probable 2-oxoglutarate dehydrogenase E1 component DHKTD1, mitochondrial	102.98	6.93	0.8171	0.9217
Isoform 1 of Cystathionine beta-synthase	60.55	6.65	1.1677	0.9220
Protein S100-A10	11.20	7.37	1.1914	0.9222
40S ribosomal protein S10	18.89	10.15	0.9819	0.9227
Endothelin-2	19.95	10.26	1.1470	0.9229
DnaJ homolog subfamily B member 1	38.02	8.63	1.1491	0.9231
Putative G-protein coupled receptor	43.06	5.20	1.1137	0.9232
Protein tyrosine phosphatase, receptor type, T	160.75	6.84	1.0293	0.9233
11 kDa protein	11.16	6.80	0.9717	0.9235
Isoform 2 of UPF0605 protein FAM166B	23.82	6.68	0.9458	0.9235
ATP synthase, H+ transporting, mitochondrial F0 complex, subunit B1	22.26	9.26	1.0531	0.9236
Isoform 2 of Methyl-CpG-binding domain protein 3	29.00	5.21	1.0585	0.9236
Hexokinase 3 variant (Fragment)	102.37	5.81	0.9398	0.9237
Isoform 5 of Extracellular matrix protein FRAS1	341.71	5.58	1.2915	0.9238
Isoform 1 of Docking protein 3	53.25	7.77	1.0346	0.9242
Neuroblast differentiation-associated protein AHNAK	628.70	6.15	1.0960	0.9243
Isoform 4 of ETS-related transcription factor Elf-2	54.23	8.38	0.8516	0.9244
Melanoma-associated antigen E1	103.19	5.33	0.0000	0.9246
Isoform 2 of Collagen alpha-1(XVII) chain	142.26	8.87	0.9506	0.9248
Fanconi anemia-associated protein of 24 kDa	23.88	9.19	1.0161	0.9249
Protein	17.40	8.48	1.0641	0.9250
Eukaryotic translation elongation factor 1 epsilon-1	19.80	8.54	1.2039	0.9252
Glyceraldehyde-3-phosphate dehydrogenase	36.03	8.46	0.9703	0.9253
CDNA FLJ26884 fis, clone PRS09279	16.54	8.56	1.1686	0.9254
Isoform 1 of Triosephosphate isomerase	30.77	5.92	0.9585	0.9254
Putative uncharacterized protein DKFZp781K1356	13.55	5.57	1.0760	0.9256
Plexin-A3	207.57	7.31	0.8853	0.9256
Lysyl oxidase homolog 4	84.43	7.46	1.1701	0.9257
Isoform 2 of S-phase kinase-associated protein 1	18.05	4.48	1.1475	0.9258
cDNA FLJ53581, highly similar to Ras and Rab interactor 1	65.31	8.76	1.0526	0.9259
Polypeptide N-acetylgalactosaminyltransferase 5	106.20	9.47	1.2132	0.9260
Mucin 15 isoform c	33.77	5.21	1.0881	0.9260
Tumor differentiation factor	12.70	7.96	1.0032	0.9261
Isoform 6 of Noelin-3	16.78	8.53	1.1956	0.9261
Integrin alpha-IIb variant (Fragment)	59.19	6.05	0.8027	0.9263

61 kDa protein	61.38	5.21	0.9113	0.9264
cDNA FLJ53975, highly similar to Acetyl-CoA acetyltransferase, cytosolic	44.61	8.46	0.8783	0.9264
Transgelin	22.60	8.84	1.0290	0.9265
SWI/SNF complex subunit SMARCC1	122.79	5.76	0.9875	0.9265
Putative uncharacterized protein ENAH	8.86	8.43	0.8695	0.9266
85 kDa protein	84.55	5.66	0.9442	0.9267
Laminin subunit alpha-1	336.94	6.35	1.1319	0.9268
Bloom syndrome protein	158.90	7.49	0.5043	0.9274
40S ribosomal protein S25	13.73	10.11	0.9610	0.9275
Integrin alpha-V isoform 2	111.08	5.62	0.8946	0.9276
C-type lectin domain family 7, member A, isoform CRA_j	13.13	6.67	0.8942	0.9276
MYRIP protein	83.90	5.31	1.0505	0.9277
18 kDa protein	17.72	8.19	0.9262	0.9277
Putative uncharacterized protein DKFZp564G0422	12.40	9.57	0.7598	0.9280
Isoform 2 of Receptor tyrosine-protein kinase erbB-3	20.12	5.48	0.9952	0.9282
Clusterin-like protein 1	54.18	5.19	1.0601	0.9283
Similar to peptidyl-Pro cis trans isomerase	18.10	6.89	0.9400	0.9284
Lamina-associated polypeptide 2, isoform alpha	75.45	7.66	0.9341	0.9284
13 kDa protein	13.29	4.55	1.0462	0.9285
Sorting nexin 6 isoform a	33.55	8.57	0.8949	0.9285
Ubiquitin carboxyl-terminal hydrolase 3	58.86	8.18	1.3491	0.9288
TRPM2 protein	176.45	7.80	0.9683	0.9289
Kinetochore protein NDC80 homolog	73.87	5.60	1.2139	0.9289
cDNA FLJ56853, highly similar to Epsin-2	28.30	9.48	1.9104	0.9291
Putative uncharacterized protein ENSP00000371928	25.02	8.32	1.1010	0.9292
2 kDa protein	1.95	8.84	1.2332	0.9294
Keratin, type II cytoskeletal 2 oral	65.83	8.12	1.0455	0.9294
Isoform 3 of Gamma-interferon-inducible protein Ifi-16	75.89	9.36	1.0228	0.9295
sterol carrier protein 2 isoform 2 precursor	15.07	8.92	0.8379	0.9297
Putative uncharacterized protein SLC2A1 (Fragment)	54.04	8.87	1.7091	0.9302
Isoform 2 of Protein FAM40A	85.40	6.74	0.8356	0.9306
Nucleosome assembly protein 1-like 1, isoform CRA_c	37.32	4.67	1.0439	0.9306
Kinesin-like protein KIF7	150.49	6.79	1.0683	0.9307
cDNA FLJ58032, highly similar to GTP:AMP phosphotransferase mitochondrial	21.00	9.28	1.0241	0.9307
19 kDa protein	19.04	9.07	1.1136	0.9307
54 kDa protein	54.13	7.27	0.9504	0.9308
MHC class I antigen	9.21	11.34	1.0269	0.9308
Immediate early response gene 2 protein	24.18	6.92	0.9926	0.9309
Isoform 2 of Protein FAM83A	32.14	9.16	0.8640	0.9310
Isoform 4 of Disks large homolog 5	201.93	7.71	0.9097	0.9310
Sulfotransferase 1A1	44.12	8.54	0.9432	0.9311
Isoform 3 of Peripheral plasma membrane protein CASK	102.05	6.35	0.9529	0.9313
Kinesin-like protein KIF3C	89.40	8.37		0.9315
Zinc finger protein 84	85.40	8.78	0.9837	0.9315
Ubiquitin domain-containing protein 1	25.92	7.83	1.3983	0.9317
Phosphoglycerate kinase 1	44.59	8.10	0.9956	0.9317
cDNA FLJ16146 fis, clone BRAMY2040159, highly similar to Homo sapiens MRIP-1 mRNA	81.36	7.15	1.1124	0.9319
Isoform 4 of Uncharacterized protein C2orf67	73.19	8.65	1.0661	0.9321
Protein	15.29	4.04	1.1069	0.9324
Small nuclear ribonucleoprotein Sm D2	13.52	9.91	1.0438	0.9324
HSPA12B protein (Fragment)	75.86	9.09	0.9065	0.9325
cDNA FLJ33235 fis, clone ASTRO2002202	22.75	9.55	1.0304	0.9325
Glutaredoxin-3	37.41	5.39	0.9207	0.9329
acidic (leucine-rich) nuclear phosphoprotein 32 family, member E isoform 3	25.11	3.74	0.9682	0.9329
Protein fem-1 homolog B	70.22	6.61	0.9593	0.9331
Ladinin-1	57.10	9.67	0.9050	0.9332

LARP4 protein	64.50	6.00	0.9853	0.9333
Isoform 2 of Roundabout homolog 2	130.59	6.58	0.8862	0.9336
6 kDa protein	5.89	5.15	0.8808	0.9337
Isoform 2 of F-actin-capping protein subunit beta	30.61	6.00	0.9129	0.9341
HP protein	31.36	8.29	0.7901	0.9344
Isoform 1 of SEL1-like repeat-containing protein KIAA0746	128.49	7.28	0.9510	0.9344
Putative uncharacterized protein SEPT2	36.92	6.18	0.9729	0.9345
HCG2042771	88.32	5.86	1.0426	0.9345
Putative uncharacterized protein FAM3B	28.26	8.73	1.0626	0.9346
Heterogeneous nuclear ribonucleoprotein L	64.09	8.22	0.9044	0.9349
Putative uncharacterized protein gs52	47.93	8.18	1.0192	0.9350
Putative uncharacterized protein DKFZp434I1016 (Fragment)	15.38	8.97	0.8695	0.9352
Sterol regulatory element-binding protein 2	123.61	8.40	1.1676	0.9353
84 kDa protein	84.21	6.62	0.9666	0.9354
YTH domain containing 2	160.15	8.40	1.1153	0.9355
Isoform 1 of Peroxisomal proliferator-activated receptor A-interacting complex 285 kDa protein	294.51	7.52	1.0938	0.9357
cDNA FLJ59503, highly similar to Semaphorin-5B	116.02	7.46	1.1706	0.9357
Isoform 1 of Rab3 GTPase-activating protein non-catalytic subunit	155.89	5.62	0.7640	0.9360
Nuclear autoantigenic sperm protein	45.77	4.36	0.9471	0.9360
Scavenger mRNA-decapping enzyme DcpS	38.59	6.38	1.2025	0.9360
VGPW2523	35.94	5.35	0.9776	0.9361
similar to hCG1777996	25.11	6.40	1.0112	0.9363
F-box protein 43 isoform a	74.40	8.18	1.2661	0.9363
hyaluronan-mediated motility receptor isoform d	74.45	5.43	1.0364	0.9364
Calcium-binding protein 39	39.84	6.89	1.2958	0.9365
Putative uncharacterized protein ERCC5 (Fragment)	13.16	11.05	1.0536	0.9368
Isoform 2 of 5'-3' exoribonuclease 2	99.90	8.02	0.9434	0.9368
Double-stranded RNA-specific editase B2	80.57	10.17	1.3363	0.9373
Isoform 5 of Coiled-coil domain-containing protein 14	82.91	6.60	1.0662	0.9375
57 kDa protein	57.18	7.59	1.0783	0.9376
Calpain-7	92.59	7.65	2.4357	0.9376
Eukaryotic translation initiation factor 3, subunit 3 gamma, 40kDa, isoform CRA_b	41.55	7.33	0.9631	0.9376
cDNA FLJ60984, highly similar to Deleted in lung and esophageal cancer protein1	48.05	9.38	0.9011	0.9377
Complement factor H-related protein 5	64.38	7.06	0.9112	0.9377
Cartilage matrix protein	53.67	7.69	1.0539	0.9378
Beta-hexosaminidase subunit beta	63.07	6.76	0.9919	0.9379
Calpain-1 catalytic subunit	81.84	5.67	1.2051	0.9381
Lactoylglutathione lyase	20.76	5.31	0.9360	0.9381
Zinc finger, C2H2-type domain containing protein	32.04	8.44	0.9209	0.9385
Isoform 1 of Regulator of microtubule dynamics protein 3	52.09	5.10	0.7329	0.9385
60S ribosomal protein L34	13.28	11.47	0.9941	0.9389
Solute carrier family 25, member 27 (Fragment)	17.38	9.88	1.3244	0.9390
Heparan sulfate glucosamine 3-O-sulfotransferase 3B1	43.30	9.63	0.9253	0.9390
11 kDa protein	11.27	10.24	1.1783	0.9391
Receptor-interacting serine/threonine-protein kinase 1	75.88	6.33	0.9907	0.9398
Plexin-C1	175.63	7.61	0.9354	0.9400
DEAH (Asp-Glu-Ala-His) box polypeptide 34	128.04	7.56	1.0979	0.9402
neuroblastoma breakpoint family, member 6 isoform 1	75.34	4.94	0.8105	0.9402
cDNA FLJ56387, highly similar to Mus musculus armadillo repeat containing 8 (Armc8), mRNA	67.86	6.98	1.0528	0.9403
UPF0258 protein KIAA1024	102.93	7.31	0.9779	0.9403
Seryl-tRNA synthetase, cytoplasmic	58.74	6.43	0.9156	0.9403
Profilin-1	15.04	8.27	0.9856	0.9404
Autogenous vein graft remodeling associated protein 2	36.26	11.63	0.9538	0.9404
Alpha-1,4-N-acetylglucosaminyltransferase	39.47	6.89	1.3662	0.9405

Isoform 1 of Nucleoside diphosphate kinase A	17.14	6.19	0.9291	0.9406
Isoform 5 of Multidrug resistance-associated protein 9	104.59	9.29	1.1658	0.9406
cDNA FLJ55055, moderately similar to Syntenin-1	26.50	6.64	0.9507	0.9406
13 kDa protein	13.23	9.86	0.9452	0.9408
Contactin-associated protein 1	156.17	7.05	0.9961	0.9410
echinoderm microtubule associated protein like 1 isoform a	91.93	7.43	1.0142	0.9413
Proteasome subunit beta type-1	26.47	8.13	1.0820	0.9415
Isoform 2 of Uncharacterized protein C22orf30	220.24	5.96	1.0161	0.9415
Putative uncharacterized protein MSN (Fragment)	67.64	6.40	0.9106	0.9416
Glutamate--cysteine ligase regulatory subunit	30.71	6.02	0.8672	0.9416
Putative uncharacterized protein OR6W1P (Fragment)	33.46	8.28	1.1122	0.9416
Isoform 4 of Rho guanine nucleotide exchange factor 10	148.23	5.78	0.8437	0.9419
12 kDa protein	11.55	8.35	0.9616	0.9420
Protein	19.61	9.26	1.0523	0.9421
Isoform 3 of Latent-transforming growth factor beta-binding protein 4	169.37	5.29	0.8717	0.9423
Probable leucyl-tRNA synthetase, mitochondrial	101.91	8.22	0.8891	0.9424
6 kDa protein	5.74	10.80	0.9899	0.9425
cDNA FLJ51828, highly similar to BAI1-associated protein 3	44.29	6.89	1.1318	0.9427
Isoform 1 of Schlafen family member 5	100.99	8.22	0.9634	0.9428
12 kDa protein	12.44	8.03	0.9502	0.9430
Delta(3,5)-Delta(2,4)-dienoyl-CoA isomerase, mitochondrial	35.79	8.00	0.9780	0.9430
cDNA FLJ59067, moderately similar to Zinc finger protein 595	67.80	8.98	1.6022	0.9434
Isoform 4 of LETM1 domain-containing protein LETM2, mitochondrial	30.89	8.60	0.8450	0.9435
hypothetical protein LOC285600 isoform 1	147.67	6.61	0.9165	0.9435
Putative uncharacterized protein ANXA5 (Fragment)	35.78	5.05	1.0020	0.9438
Putative uncharacterized protein DKFZp434F0116	53.30	9.16	1.1365	0.9439
Isoform 1 of EF-hand domain-containing family member C2	87.34	7.37	0.8356	0.9440
Isoform 2 of Protein CLEC16A	103.48	6.61	0.8885	0.9441
Isoform 1 of Ly6/PLAUR domain-containing protein 4	26.75	8.38	0.9650	0.9442
SH2 domain-containing adapter protein E	53.92	9.16	1.2130	0.9444
Calpain small subunit 1	28.30	5.20	1.1261	0.9450
Isoform 2 of Uncharacterized protein C6orf170	126.47	6.90	1.2838	0.9450
NFAT activation molecule 1	29.67	8.81	1.0488	0.9450
102 kDa protein	102.21	9.32	1.0083	0.9453
similar to high-mobility group box 3, partial	24.78	9.51	1.1375	0.9455
Isoform 1 of Spectrin alpha chain, brain	284.36	5.35	0.8886	0.9456
Importin-9	115.89	4.81	1.1211	0.9457
10 kDa protein	9.80	9.99	0.9559	0.9458
cDNA FLJ35535 fis, clone SPLEN2002419, highly similar to EGF-containing fibulin-like extracellular matrix protein 1	39.42	5.54	0.9932	0.9459
Transcription termination factor, mitochondrial	45.75	9.42	1.0431	0.9461
Isoform 1 of FERM and PDZ domain-containing protein 2	144.18	6.70	0.9732	0.9461
60S ribosomal protein L13	24.25	11.65	1.0866	0.9462
Isoform 2 of Bromodomain adjacent to zinc finger domain protein 1A	175.28	6.81	0.8605	0.9463
Isoform 4 of Coxsackievirus and adenovirus receptor	22.36	8.43	0.9951	0.9464
Inosine-5'-monophosphate dehydrogenase 2	55.77	6.90	0.9782	0.9467
Protein	10.08	10.92	0.9237	0.9467
cDNA FLJ40406 fis, clone TESTI2037534, weakly similar to 1-PHOSPHATIDYLINOSITOL-4,5-BISPHOSPHATE PHOSPHODIESTERASE DELTA 1	44.79	6.48	0.9340	0.9471
Protein	9.04	4.50	0.9534	0.9471
Protein-tyrosine sulfotransferase 2	41.88	9.09	0.8528	0.9473
Peroxiredoxin-1	22.10	8.13	0.8531	0.9477
cDNA FLJ55574, highly similar to Calnexin	71.46	4.70	1.0806	0.9478
Isoform 2 of Inter-alpha-trypsin inhibitor heavy chain H4	101.15	6.65	1.1234	0.9479
Stress-70 protein, mitochondrial	73.63	6.16	0.9124	0.9480
Isoform Short of Probable phospholipid-transporting ATPase IA	129.54	7.25	1.1963	0.9480
Baculoviral IAP repeat-containing protein 8	27.10	7.27	1.0477	0.9480

10 kDa heat shock protein, mitochondrial	10.92	8.92	0.7566	0.9481
Isoform 1 of Tripartite motif-containing protein 54	40.27	5.22	1.1211	0.9481
Peptidyl-prolyl cis-trans isomerase-like 1	18.23	7.99	1.0176	0.9481
Isoform TRPDI of Tetratricopeptide repeat protein 3	229.75	7.56	1.0537	0.9484
Isoform 2 of 26S protease regulatory subunit 6B	43.48	5.26	0.9727	0.9487
cDNA FLJ51570, highly similar to Thioredoxin domain-containing protein 1	22.38	5.71	0.8876	0.9487
Calcium-regulated heat stable protein 1	15.88	8.21	0.9213	0.9489
Ras-related protein Rab-10	22.53	8.38	1.1978	0.9491
Ribonuclease inhibitor	49.94	4.82	0.9738	0.9492
hypothetical protein LOC389161	61.19	9.20	1.1153	0.9493
MUM1-like protein 1	78.99	4.97	1.0188	0.9495
Isoform 1 of Serine protease HTRA2, mitochondrial	48.81	10.07	0.8628	0.9497
40S ribosomal protein S18	17.71	10.99	0.9872	0.9497
Isoform 2 of Long palate, lung and nasal epithelium carcinoma-associated protein 1	34.43	8.91	0.8874	0.9498
sulfatase 2 isoform b precursor	98.12	9.04	0.9758	0.9498
Protein S100-A13	11.46	6.16	0.8882	0.9501
Isoform D of Constitutive coactivator of PPAR-gamma-like protein 1	117.33	8.87	1.0295	0.9508
12 kDa protein	11.54	8.40	1.1423	0.9509
cDNA FLJ42873 fis, clone BRHIP2026061	27.14	11.82	0.8860	0.9510
cDNA FLJ59036, highly similar to von Willebrand factor	308.99	5.48	1.0468	0.9510
cDNA FLJ54453, highly similar to Glutaminyl-tRNA synthetase	82.19	7.78	1.0665	0.9513
cDNA FLJ51009, highly similar to Leukotriene A-4 hydrolase	57.19	6.19	0.9805	0.9515
Putative uncharacterized protein ENSP00000375493	18.85	9.07	1.2169	0.9519
34 kDa protein	33.61	5.12	1.3083	0.9519
Isoform 2 of DENN domain-containing protein 2C	94.45	8.66	1.1259	0.9521
cDNA FLJ40448 fis, clone TESTI2040815	19.75	8.69	1.1419	0.9522
Putative uncharacterized protein NLRP5 (Fragment)	132.38	6.46	1.3264	0.9522
similar to hCG27427	27.79	9.73	1.1298	0.9522
Isoform 1 of Attractin-like protein 1	152.54	7.31	1.1470	0.9522
Isoform 3 of Protein SPT2 homolog	40.36	9.66	1.1382	0.9522
cDNA FLJ52702, highly similar to Homo sapiens CD44 antigen (homing function and Indian blood group system) (CD44), transcript variant 4, mRNA	35.95	5.90	0.9206	0.9523
Isoform 3 of Fragile X mental retardation syndrome-related protein 1	59.88	6.77	0.8347	0.9523
cryptochrome 2 (photolyase-like) isoform 2	60.55	7.96	1.2122	0.9523
Midasin	632.42	5.68	0.9968	0.9525
Neprilysin	85.46	5.73	1.1451	0.9526
Isoform 3 of Inorganic pyrophosphatase 2, mitochondrial	34.64	8.46	0.8917	0.9526
Isoform 2 of Reticulon-3	110.58	4.97	1.2040	0.9527
Isoform 2 of Acidic leucine-rich nuclear phosphoprotein 32 family member B	22.26	4.30	0.8882	0.9527
Isoform 1 of Myomegalin	264.92	5.44	1.0937	0.9529
Vacuolar protein sorting-associated protein 35	91.65	5.49	0.9833	0.9529
ALS2CR11 protein (Fragment)	49.61	9.70	1.0720	0.9529
G-protein coupled receptor-associated sorting protein 2	93.71	5.01	0.9482	0.9531
similar to acyl-CoA synthetase medium-chain family member 5	61.42	7.75	1.2708	0.9533
peroxiredoxin 3 isoform b	25.82	7.46	0.9141	0.9534
cDNA FLJ56882, highly similar to Activating signal cointegrator 1 complex subunit 3	250.35	7.11	1.0706	0.9534
Proteasome activator complex subunit 1	28.71	6.02	1.0879	0.9536
Isoform SM-B of Small nuclear ribonucleoprotein-associated proteins B and B'	23.64	10.90	1.0258	0.9537
40S ribosomal protein S14	16.26	10.05	1.0545	0.9537
cDNA FLJ51602, highly similar to Interferon-induced guanylate-binding protein 1	41.24	6.73	1.1846	0.9538
Purine nucleoside phosphorylase	32.10	6.95	1.0084	0.9538
Putative uncharacterized protein TSGA10	72.41	5.90	1.1892	0.9538
5 kDa protein	4.73	9.66	1.1171	0.9539
Similar to Dual specificity mitogen-activated protein kinase kinase 2	42.46	5.99	1.1413	0.9539
PRO1155	9.26	5.39	0.9123	0.9539
protein kinase C substrate 80K-H isoform 2	59.14	4.42	0.9394	0.9540

small acidic protein isoform b	15.45	4.55	0.8883	0.9541
Protein	14.66	10.05	0.9571	0.9542
Isoform 2 of ATP-binding cassette sub-family A member 7	218.36	6.98	0.9786	0.9542
Isoform 7 of Membrane-associated guanylate kinase, WW and PDZ domain-containing protein 1	157.75	7.50	0.9220	0.9543
Homeobox protein aristaless-like 3	36.91	8.59	0.9460	0.9544
FKBP12-rapamycin complex-associated protein	288.71	7.17	1.0545	0.9544
Isoform 1 of Copine-4	62.35	6.33	1.5646	0.9545
41 kDa protein	41.24	7.25	0.9314	0.9546
14-3-3 protein zeta/delta	27.73	4.79	1.0062	0.9547
GTP-binding nuclear protein Ran	24.41	7.49	1.0270	0.9547
Isoform 1 of 60S ribosomal protein L12	17.81	9.42	1.0329	0.9548
Activated RNA polymerase II transcriptional coactivator p15	14.39	9.60	0.9898	0.9549
Isoform 2 of Tubulin polyglutamylase complex subunit 1	30.57	9.03	1.1007	0.9552
Similar to ribosomal protein L18	24.91	9.70	1.0963	0.9554
Postmeiotic segregation increased 2-like protein 8	42.94	9.86	0.9264	0.9556
190 kDa protein	189.98	7.39	0.9349	0.9557
aldehyde dehydrogenase 7 family, member A1	58.45	7.99	1.0744	0.9562
caspase 5 isoform c	33.28	8.44	0.6113	0.9563
Peroxiredoxin-6	25.02	6.38	0.9536	0.9567
DNA-(apurinic or apyrimidinic site) lyase	35.53	8.12	0.9152	0.9568
Isoform 3 of 3-keto-steroid reductase	34.45	8.16	0.8979	0.9569
Protein phosphatase 1 regulatory subunit 14B	20.95	5.22	1.1073	0.9569
Ubiquitin C splice variant	17.14	8.34	1.1658	0.9569
Non-histone chromosomal protein	17.51	9.79	0.8839	0.9570
CAAX prenyl protease 1 homolog	54.78	7.49	0.8961	0.9570
Isoform Alpha of Caveolin-1	20.46	6.02	0.9346	0.9570
apoptosis inhibitor 5 isoform c	50.71	6.18	1.0986	0.9570
cDNA FLJ53090, moderately similar to RNA-binding motif, single-stranded-interacting protein 2	30.85	9.03	0.9498	0.9572
Parafibromin	60.54	9.61	1.0467	0.9574
Isoform 2 of Dynein heavy chain 1, axonemal	485.63	6.05	0.9840	0.9577
Isoform 2 of Nucleoporin p54	33.16	6.37	0.7520	0.9579
Isoform 2 of Trinucleotide repeat-containing gene 6A protein	181.94	6.58	0.9665	0.9579
DDB1- and CUL4-associated factor 15	66.42	6.58	0.9463	0.9583
ATP-binding cassette sub-family D member 2	83.18	8.92	0.9072	0.9583
Transitional endoplasmic reticulum ATPase	89.27	5.26	0.9919	0.9587
22 kDa protein	21.67	9.66	1.0977	0.9589
Thioredoxin domain-containing protein 4	46.94	5.26	0.8883	0.9590
Isoform 1 of Hydroxyacyl-coenzyme A dehydrogenase, mitochondrial	34.26	8.85	0.9940	0.9591
Tubulin, alpha 1 (Testis specific), isoform CRA_a	48.30	5.01	1.0852	0.9591
Protein DJ-1	19.88	6.79	1.0103	0.9591
Cyclin-dependent kinase 4 inhibitor D	17.69	6.13	1.1015	0.9592
Isoform 2 of Dynein heavy chain 8, axonemal	510.12	6.29	0.8696	0.9594
inositol(myo)-1(or 4)-monophosphatase 1 isoform 3	21.94	5.38	1.0036	0.9599
23 kDa protein	23.19	7.20	0.9194	0.9600
Isoform 2 of Glycosyltransferase 8 domain-containing protein 3	46.77	9.03	0.8701	0.9600
Isoform A of Calcium/calmodulin-dependent protein kinase type II alpha chain	54.00	7.20	1.0547	0.9600
Isoform 1 of Beta-2-syntrophin	57.91	8.82	1.1076	0.9602
Kinesin-like protein KIF1C	122.87	6.90	0.9431	0.9603
Dopey family member 1	266.39	6.40	0.8610	0.9603
Isoform 2 of GTPase-activating Rap/Ran-GAP domain-like protein 3	92.99	7.53	1.0366	0.9603
Proteasome subunit alpha type-5	26.39	4.79	0.9487	0.9605
Isoform 3 of LIM/homeobox protein Lhx9	36.03	9.01	1.1846	0.9606
125 kDa protein	124.54	6.99	0.9493	0.9607
N-acetyltransferase ESCO2	68.26	9.39	1.0114	0.9608
Putative uncharacterized protein NAP1L4	22.43	5.03	0.9854	0.9610

S-adenosylhomocysteine hydrolase-like 2 isoform b	66.55	7.36	0.8771	0.9611
Isoform 1 of Dynamin-binding protein	177.24	5.39	0.9977	0.9611
Isoform 1 of Coiled-coil domain-containing protein C6orf204	91.75	6.33	0.9427	0.9613
UDP-glucuronosyltransferase 1-6	60.71	8.41	1.0431	0.9614
cDNA FLJ45740 fis, clone KIDNE2014496	23.10	11.71	0.9142	0.9615
similar to hCG2041004	16.29	8.44	0.8799	0.9618
Isoform 1 of Chondrolectin	30.41	6.83	0.7799	0.9619
NKG2D ligand 1	27.98	7.42	1.0653	0.9619
PRO2866	5.59	9.42	1.1857	0.9621
Peroxisomal multifunctional enzyme type 2	79.64	8.84	0.9139	0.9625
cDNA FLJ57583, highly similar to Homo sapiens transcription factor MLR1 (MLR1), mRNA	16.29	4.81	1.0127	0.9626
Chromosome 9 open reading frame 84	157.00	5.50	1.2810	0.9627
LAMP1 protein variant (Fragment)	41.80	7.87	0.7732	0.9628
Dynactin 2 (P50), isoform CRA_b	34.47	6.06	0.9338	0.9629
Phosphatidylinositol-4,5-bisphosphate 3-kinase catalytic subunit gamma isoform	126.37	7.50	0.9048	0.9630
CAD protein	242.83	6.46	1.0196	0.9630
Dystonin	856.92	5.24	1.1238	0.9631
Condensin complex subunit 1	157.07	6.57	0.9568	0.9632
Acylamino-acid-releasing enzyme	81.17	5.48	1.0781	0.9632
similar to hCG1643655	43.18	8.46	0.9748	0.9634
ERO1-like protein alpha	54.36	5.68	1.0873	0.9636
Neugrin	45.48	9.99	1.0894	0.9636
cDNA FLJ52249, highly similar to U1 small nuclear ribonucleoprotein A	22.16	9.82	1.0010	0.9636
Isoform 2 of Janus kinase and microtubule-interacting protein 1	97.38	5.80	1.1045	0.9637
Proopiomelanocortin	27.05	6.57	0.9736	0.9639
Isoform 2 of Interleukin-21	16.86	9.45	0.9760	0.9643
Ankyrin repeat and BTB/POZ domain-containing protein 2	93.23	5.90	1.2527	0.9644
Isoform 6 of Protein GRINL1A	48.12	7.17	0.9619	0.9645
Prostaglandin E2 receptor EP2 subtype	39.73	9.10	0.9235	0.9646
Isoform Long of Eukaryotic translation initiation factor 4H	27.37	7.23	0.9685	0.9646
phosphoribosylaminoimidazole carboxylase, phosphoribosylaminoimidazole succinocarboxamide synthetase isoform 1	47.93	7.69	1.0222	0.9646
Isoform 1 of Tripartite motif-containing protein 35	56.50	7.11	0.5736	0.9647
Isoform 4 of Protein WWC2	128.61	5.90	0.9493	0.9648
Isoform 1 of Heterogeneous nuclear ribonucleoprotein K	50.94	5.54	1.0162	0.9648
182 kDa protein	181.70	4.45	0.9464	0.9649
Isoform 1 of Ras-related protein Rab-19	31.78	8.00	1.2431	0.9654
Isoform 1 of Dynein heavy chain 12, axonemal	338.51	6.44	1.4969	0.9654
Isoform Short of 14-3-3 protein beta/alpha	27.83	4.83	0.9621	0.9655
40S ribosomal protein S9	22.58	10.65	1.0983	0.9657
40S ribosomal protein S13	17.21	10.54	1.0431	0.9657
Homeobox protein Nkx-3.2	34.79	7.93	1.3128	0.9658
HDJ2 protein (Fragment)	29.45	5.88	0.9358	0.9658
splicing factor 3B subunit 2	100.16	5.67	0.9460	0.9660
60S ribosomal protein L26	17.25	10.55	0.7217	0.9661
FERM and PDZ domain-containing protein 3	199.08	8.15	1.0080	0.9662
cDNA FLJ30924 fis, clone FEBRA2006521, highly similar to Junctophilin-3	46.55	9.74	0.9094	0.9663
Isoform 1 of Inhibitor of growth protein 1	46.71	9.06	0.9945	0.9663
Eukaryotic translation initiation factor 3 subunit M	42.48	5.63	0.9463	0.9663
60S ribosomal protein L37a	10.27	10.43	0.9091	0.9664
cDNA, FLJ94667, highly similar to Homo sapiens hematopoietic protein 1 (HEM1), mRNA	87.02	6.38	0.9130	0.9664
Isoform Short of Proteasome subunit alpha type-1	29.54	6.61	0.9865	0.9665
Uncharacterized protein KIAA1737	42.67	8.95	1.1608	0.9665
Putative uncharacterized serine/threonine-protein kinase SgK069	90.36	5.58	1.0429	0.9667
similar to peptidyl-Pro cis trans isomerase	25.62	8.54	0.8878	0.9669

Isoform 1 of Far upstream element-binding protein 1	67.52	7.61	0.8978	0.9670
hypothetical LOC100131655	166.39	10.21	1.0418	0.9670
Putative pre-mRNA-splicing factor ATP-dependent RNA helicase DHX15	90.88	7.46	0.9094	0.9672
cDNA FLJ55758, highly similar to Homo sapiens tubulin tyrosine ligase-like family, member 3 (TTLL3), transcript variant 1, mRNA	70.63	8.37	1.0059	0.9672
CLL-associated antigen KW-6 (Fragment)	25.87	10.43	1.1250	0.9672
RUN domain-containing protein 2B	27.21	4.94	1.0259	0.9672
Isoform 5 of Minor histocompatibility antigen H13	36.79	6.68	1.1111	0.9674
Cytochrome P450 2D6	55.70	7.42	0.8117	0.9675
Small nuclear ribonucleoprotein E	10.80	9.44	1.0384	0.9676
Cytochrome b-c1 complex subunit 2, mitochondrial	48.41	8.63	1.0352	0.9677
F-box only protein 39	52.61	8.88	2.0069	0.9678
26S proteasome non-ATPase regulatory subunit 7	37.00	6.77	1.3892	0.9678
hypothetical protein	22.92	9.29	1.0233	0.9678
Cartilage intermediate layer protein 2 precursor	126.78	8.27	0.5345	0.9679
Plastin-2	70.24	5.33	1.1274	0.9679
Coatomer subunit beta	107.07	6.05	1.0580	0.9679
Chromobox protein homolog 3	20.80	5.33	0.9572	0.9679
Inositol 1,4,5-trisphosphate receptor type 3	303.91	6.48	0.9657	0.9679
40S ribosomal protein S3a	29.93	9.73	0.9347	0.9680
Beta-klotho	119.73	9.22	1.1931	0.9680
Small ubiquitin-related modifier 4	10.68	7.18	0.9822	0.9680
serine peptidase inhibitor, Kazal type 5 isoform a precursor	124.00	8.09	0.8455	0.9682
HERV-H_3q26 provirus ancestral Gag polyprotein	23.39	9.03	0.9419	0.9682
88 kDa protein	87.94	5.39	1.0132	0.9682
Phosphoglycerate mutase 1	28.79	7.18	1.0025	0.9683
GRAM domain-containing protein 2	40.22	8.43	1.2250	0.9683
Mitochondrial brown fat uncoupling protein 1	32.98	9.09	1.0050	0.9683
Galectin-3	26.14	8.56	1.3569	0.9683
Malate dehydrogenase, mitochondrial	35.48	8.68	0.8986	0.9684
Constitutive androstane receptor SV18	16.59	9.22	1.0078	0.9684
Isoform 3 of Urokinase plasminogen activator surface receptor	32.01	6.25	1.0966	0.9685
Isoform 2 of KH domain-containing, RNA-binding, signal transduction-associated protein 1	45.83	7.58	1.0674	0.9688
T-complex protein 1 subunit delta	53.10	7.99	1.0023	0.9689
Isoform 1 of Histone-lysine N-methyltransferase, H3 lysine-79 specific	164.75	9.19	1.0103	0.9689
Small GTP-binding protein	21.95	6.21	0.9765	0.9689
Elongation factor 1-alpha 1	50.11	9.01	0.9528	0.9691
Protein tyrosine phosphatase	90.55	7.05	1.1761	0.9692
Isoform 1 of Cyclin-dependent kinase-like 3	67.47	9.31	0.9806	0.9695
Eukaryotic translation initiation factor 3 subunit G	35.59	6.13	0.8434	0.9695
Acetylcholine receptor subunit beta	56.66	5.81	1.1087	0.9696
Clone MM1 product (Fragment)	38.26	7.09	1.1572	0.9696
Isoform 2 of Serine/threonine-protein kinase D3	68.08	7.42	1.0364	0.9697
Eukaryotic translation initiation factor 5	49.19	5.58	1.0186	0.9697
cDNA FLJ59865, highly similar to WW domain-binding protein 2	22.64	8.98	0.9922	0.9699
5 kDa protein	5.20	11.84		0.9699
Uncharacterized protein KIAA0586	169.16	5.54	0.8367	0.9700
Isoform 1 of DENN domain-containing protein 5B	144.93	6.73	0.9468	0.9700
60S acidic ribosomal protein P2	11.66	4.54	0.9548	0.9701
Long-chain-fatty-acid--CoA ligase 3	80.37	8.38	1.2615	0.9701
Isoform 1 of Frataxin, mitochondrial	23.12	8.69	1.1461	0.9702
Isoform 3 of E3 ubiquitin-protein ligase UBR4	571.49	6.04	1.0211	0.9703
hypothetical protein	56.13	6.80	0.8975	0.9704
91 kDa protein	90.67	9.09	0.9584	0.9706
UPF0727 protein C6orf115	9.05	6.29	1.3305	0.9706
cDNA FLJ52301, highly similar to Dystrophin-related protein 2	107.54	6.23	1.4662	0.9707

similar to hCG1818012	28.25	6.37	0.8282	0.9707
TATA-box-binding protein	31.60	9.79	0.9971	0.9708
26 kDa protein	26.27	8.32	0.9027	0.9709
Zinc finger protein 226	91.86	8.70	0.9740	0.9709
HSPA5 protein	72.38	5.16	0.8731	0.9709
60 kDa heat shock protein, mitochondrial	61.02	5.87	0.8995	0.9710
Isoform M1 of Pyruvate kinase isozymes M1/M2	58.02	7.71	1.0210	0.9710
cDNA FLJ61435, moderately similar to Adenosine kinase	34.06	6.73	0.9596	0.9710
similar to hCG1655846	53.96	7.69	0.9087	0.9711
Isoform 5 of A-kinase anchor protein 9	447.48	4.98	1.0425	0.9711
Isoform 13 of CASP8 and FADD-like apoptosis regulator	23.27	7.96	0.9682	0.9712
RFT1 homolog (S. cerevisiae), isoform CRA_b	55.80	8.72	1.1922	0.9713
Isoform 1 of Probable G-protein coupled receptor 116	149.36	6.65	0.9046	0.9715
88 kDa protein	87.50	7.44	1.3536	0.9715
Isoform 3 of Fermitin family homolog 1	26.48	8.00	0.9358	0.9715
EMILIN-2	115.54	6.39	0.9707	0.9717
Receptor-type tyrosine-protein phosphatase beta	224.13	7.68	1.1749	0.9718
Leucine-rich repeat-containing protein 59	34.91	9.57	1.0446	0.9719
Isoform M1 of Microphthalmia-associated transcription factor	46.91	6.25	1.3418	0.9719
cDNA FLJ60371, highly similar to Acetyl-CoA carboxylase 2	267.87	6.33	1.1234	0.9719
ATP-dependent RNA helicase DDX3X	73.20	7.18	1.0481	0.9719
40S ribosomal protein S30	6.64	12.15	0.9566	0.9721
cDNA FLJ32252 fis, clone PROST1000167, weakly similar to SYNAPSIN I	37.24	11.30	0.6360	0.9722
Isoform 2 of THAP domain-containing protein 3	26.91	10.24	1.0983	0.9723
cDNA FLJ53096, highly similar to Collagen alpha-2(V) chain	39.97	4.97	1.0714	0.9724
Isoform 2 of Alanine--glyoxylate aminotransferase 2-like 2	11.92	7.97	1.3779	0.9725
Isoform 3 of Proto-oncogene tyrosine-protein kinase Fyn	54.48	6.23	1.6577	0.9726
Sortilin	92.01	5.74	0.9598	0.9727
similar to ribosomal protein L23a	17.71	10.46	1.0269	0.9727
Isoform 1 of Collagen alpha-3(IV) chain	161.71	9.16	0.9896	0.9727
Flap endonuclease 1	42.57	8.62	0.9995	0.9728
NACHT, LRR and PYD domains-containing protein 6	98.67	8.07	1.2987	0.9728
17 kDa protein	17.09	10.14	1.0396	0.9728
RNA polymerase II transcription factor SIII subunit A2	83.87	9.73		0.9731
Zinc finger protein 667	70.12	9.73	1.0372	0.9735
Prefoldin subunit 4	15.30	4.53	0.9981	0.9735
Isoform 1 of Peripherin	53.62	5.47	0.9194	0.9737
Isoform 2 of Uncharacterized protein KIAA1614	86.34	9.17	0.9625	0.9737
hypothetical protein	15.30	8.44	1.1947	0.9738
cDNA FLJ44694 fis, clone BRACE3014523	42.57	6.33	1.1380	0.9738
Collagen alpha-4(IV) chain	163.90	8.59	0.9661	0.9739
Isoform 2 of Inositol hexakisphosphate and diphosphoinositol-pentakisphosphate kinase 2	138.02	8.06	0.8785	0.9740
nuclear factor of kappa light polypeptide gene enhancer in B-cells inhibitor, beta isoform b	32.78	5.44	1.0694	0.9741
9 kDa protein	9.05	9.55	0.8577	0.9741
Homeobox protein Nkx-2.6	32.10	9.88	0.9711	0.9743
cDNA FLJ59832, moderately similar to Prostaglandin E synthase 3	14.56	4.31	0.9423	0.9743
Protein	5.70	8.73	0.9785	0.9744
Isoform 3 of Cell division cycle protein 16 homolog	65.82	6.07	1.2116	0.9747
Alcohol dehydrogenase [NADP+]	36.55	6.79	0.9622	0.9747
Isoform 1 of Kynurenine/alpha-aminoadipate aminotransferase mitochondrial	47.32	6.96	0.9204	0.9748
6 kDa protein	5.71	9.70	0.7566	0.9748
FACT complex subunit SSRP1	81.02	6.87	0.8309	0.9749
LOC284804 protein (Fragment)	22.37	10.89	0.9213	0.9752
cDNA FLJ53327, highly similar to Gelsolin	77.74	5.69	0.8367	0.9752
Isoform 3 of NACHT, LRR and PYD domains-containing protein 1	157.22	6.83	1.0024	0.9753

10 kDa protein	9.69	10.29	0.9165	0.9754
cDNA FLJ60782, highly similar to Rho-GTPase-activating protein 1	52.72	6.70	1.1607	0.9755
cDNA FLJ50200, highly similar to DENN domain-containing protein 1A	86.16	6.20	1.1060	0.9755
Isoform 1 of Vinculin	116.65	6.09	1.0804	0.9757
Isoform 1 of Ras-related protein Rab-15	24.38	5.71	0.9570	0.9758
Isoform 2 of RNA polymerase II subunit A C-terminal domain phosphatase	93.41	6.42	0.9298	0.9758
Protein	12.80	11.00	0.8899	0.9759
cDNA FLJ16541 fis, clone OCBBF2034823, highly similar to Mus musculus widely-interspaced zinc finger motifs (Wiz), transcript variant 1, mRNA	89.30	9.48	1.2759	0.9760
Isoform 1 of COP9 signalosome complex subunit 2	51.56	5.53	0.9650	0.9761
42 kDa protein	42.31	5.92	1.0535	0.9763
Keratin, type II cuticular Hb5	55.77	6.55	1.1095	0.9763
33 kDa protein	32.72	5.78	1.4900	0.9763
cDNA FLJ45045 fis, clone BRAWH3021580, highly similar to Restin	131.54	5.30	0.9706	0.9763
Annexin A1	38.69	7.02	1.2687	0.9764
Sterile alpha motif domain-containing protein 9	184.16	7.83	0.9074	0.9764
similar to hCG1742309	16.51	9.04	1.0876	0.9764
11 kDa protein	11.33	9.66	0.9712	0.9765
Isoform 3 of Formin-binding protein 1-like	63.53	6.19	0.8108	0.9767
TATA-binding protein-associated factor 172	206.76	6.52	0.9331	0.9767
eukaryotic translation initiation factor 2C, 3 isoform b	71.17	9.01	0.8717	0.9768
11 kDa protein	10.68	8.84	0.8119	0.9768
similar to hCG1646697	48.68	10.59	0.8011	0.9768
Retinoblastoma-associated protein	106.09	7.94	1.2533	0.9769
Isoform Long of Splicing factor, proline- and glutamine-rich	76.10	9.44	0.9650	0.9769
cDNA FLJ53884, highly similar to mRNA decapping enzyme 1A	59.29	6.64	1.0601	0.9769
Isoform Cytoplasmic of Fumarate hydratase, mitochondrial	50.18	7.40	0.9026	0.9770
Isoform Short of Delta-1-pyrroline-5-carboxylate synthetase	87.03	7.12	1.0500	0.9770
Isoform 1 of Probable tubulin polyglutamylase TTLL10	74.99	9.23	1.0610	0.9771
cDNA FLJ16186 fis, clone BRTHA2007060, moderately similar to EUKARYOTIC TRANSLATION INITIATION FACTOR 3 SUBUNIT 10	111.71	9.33	0.9750	0.9771
60S ribosomal protein L35	14.54	11.05	0.8949	0.9772
poly(A) binding protein interacting protein 1 isoform 3	41.98	4.51	0.8996	0.9773
Isoform 2 of Uncharacterized protein KIAA0754	104.59	4.31	0.9236	0.9774
Isoform 1 of Keratin, type II cytoskeletal 73	58.89	7.23	0.9860	0.9775
Basement membrane-specific heparan sulfate proteoglycan core protein	468.50	6.51	1.6593	0.9777
Apolipoprotein L, 4	15.28	7.40	1.0256	0.9777
cDNA, FLJ96653, Homo sapiens VAMP (vesicle-associated membrane protein)-associated protein A, 33kDa (VAPA), mRNA	27.30	8.62	0.9678	0.9780
14-3-3 protein epsilon	29.16	4.74	1.1234	0.9782
transmembrane protein 118 isoform 2	46.01	8.13	0.8847	0.9782
11 kDa protein	11.22	4.97	1.0227	0.9782
Dehydrogenase/reductase SDR family member 7C	34.86	8.47	1.0756	0.9786
Isoform 3 of Protein tyrosine phosphatase type IVA 3	10.49	10.33	0.8538	0.9786
hypothetical protein	23.73	9.55	0.8970	0.9787
Isoform 1 of SH3 domain-binding protein 4	107.43	7.71	0.5873	0.9787
cDNA FLJ55501, highly similar to Rattus norvegicus basic leucine zipper and W2 domains 1 (Bzw1), mRNA	48.48	5.92	1.1707	0.9788
Proteasome subunit alpha type-6	27.38	6.76	0.8586	0.9788
Isoform 1c of Oxysterol-binding protein-related protein 3	97.26	6.92	1.1217	0.9788
cDNA FLJ39113 fis, clone NTONG2005897, highly similar to Calsyntenin-2	67.79	6.52	1.1449	0.9792
Hypothetical short protein	5.95	10.40	0.8538	0.9794
Hsp90 co-chaperone Cdc37	44.44	5.25	1.0730	0.9795
WD repeat and HMG-box DNA binding protein 1 isoform 2	112.63	5.73	1.0067	0.9796
LYR motif-containing protein 2	10.44	10.46		0.9797
Isoform 2 of TraB domain-containing protein	37.07	7.44	0.9463	0.9797
COP9 signalosome complex subunit 7a	30.26	8.22	1.1976	0.9797
cDNA FLJ61160	12.93	11.66	1.0599	0.9800

G-protein coupled receptor-associated sorting protein 1	156.77	4.68	0.9950	0.9800
Isoform 1 of Centromere protein I	86.66	8.76	0.7972	0.9800
Isoform 2 of Sharpin	33.86	6.27	1.0054	0.9800
cDNA FLJ51191, highly similar to Bromodomain-containing protein 1	87.68	9.32	0.9982	0.9800
14-3-3 protein theta	27.75	4.78	0.9913	0.9801
Conserved hypothetical protein	12.45	12.00	1.0229	0.9803
cDNA FLJ50226, moderately similar to Homo sapiens transmembrane protein 64 (TMEM64), mRNA	14.23	10.01	0.9335	0.9803
Isoform 3 of Platelet-derived growth factor C	18.77	5.54	0.8903	0.9803
Glycogen phosphorylase, brain form	96.63	6.86	1.0305	0.9803
40S ribosomal protein S15a	14.83	10.13	1.0151	0.9804
Voltage-dependent calcium channel gamma-8 subunit	43.45	9.47	0.8829	0.9804
Isoform 2 of Storkhead-box protein 2	96.06	8.35	1.1289	0.9804
Annexin A4	35.86	6.13	0.9128	0.9805
40S ribosomal protein S19	16.05	10.32	0.9581	0.9805
Uncharacterized protein C6orf58	37.90	6.15	1.0142	0.9806
Mediator complex subunit MED16 variant MED16_i5 (Fragment)	51.19	6.76	0.9529	0.9806
Sorting nexin-25	97.88	6.38	0.9861	0.9807
Heat shock protein beta-1	22.77	6.40	0.9966	0.9809
Isoform 3 of Slit homolog 2 protein	168.78	7.06	1.1014	0.9811
Testis specific basic protein	63.28	9.48	1.2673	0.9812
TIMP metallopeptidase inhibitor 1	16.05	8.07	0.9743	0.9812
cDNA FLJ32765 fis, clone TESTI2001852, weakly similar to Homo sapiens contactin associated protein (Caspr) mRNA	80.43	6.40	1.0808	0.9814
Isoform 2 of D(2) dopamine receptor	47.32	9.42	0.8262	0.9815
matrix metalloproteinase 2 isoform b	68.79	5.34	1.0152	0.9817
Isoform 2 of Hermansky-Pudlak syndrome 4 protein	58.03	5.57	0.8329	0.9818
Caspase recruitment domain-containing protein 14	113.23	5.87	1.0284	0.9818
Putative uncharacterized protein	5.74	10.27	1.0918	0.9819
cDNA FLJ53108, highly similar to Guanine nucleotide-binding protein alpha-13 subunit	40.95	7.99	1.0397	0.9820
Isoform 1 of Proteasome subunit alpha type-7	27.87	8.46	0.9677	0.9821
Ubiquitin carrier protein (Fragment)	16.23	5.57	0.9383	0.9824
Putative uncharacterized protein BSG	19.43	6.68	1.0398	0.9824
FAM98A protein	34.41	8.28	0.7851	0.9825
Citron	236.46	6.67	1.0462	0.9826
similar to hCG1783452	27.22	6.10	0.8461	0.9827
WAP four-disulfide core domain protein 10A	8.94	8.41	1.0550	0.9827
Cullin 2	80.27	7.39	1.0171	0.9827
Proteasome subunit alpha type-2	25.88	7.43	0.8876	0.9828
14-3-3 protein eta	28.20	4.84	0.9591	0.9828
Isoform 2 of M-phase phosphoprotein 9	115.65	6.28	0.8371	0.9830
Ganglioside GM2 activator	20.82	5.31	0.9792	0.9830
Isoform C of Protein CutA	16.82	5.21	1.1537	0.9832
Galectin-1	14.71	5.50	0.9075	0.9833
Uncharacterized protein C8orf16	8.84	9.67	1.1350	0.9838
ATP-binding cassette, sub-family F (GCN20), member 1	27.20	5.68	1.0563	0.9838
Putative uncharacterized protein unknown	13.34	9.57	1.0102	0.9839
Olfactory receptor 4M2	35.36	7.52	0.8613	0.9841
Ras-related protein Rab-2B	24.20	7.83	1.1121	0.9842
Isoform 1 of Folliculin-interacting protein 2	122.04	6.62	0.8639	0.9843
40S ribosomal protein S16	16.44	10.21	1.0840	0.9846
30 kDa protein	30.01	6.34	1.0609	0.9846
Putative uncharacterized protein C20orf12	58.32	7.66	0.9494	0.9847
Putative uncharacterized protein SYNPO2L	62.82	7.27	1.2208	0.9847
Uncharacterized protein C12orf4	63.76	6.37	1.0668	0.9847
shroom family member 3 protein	216.72	7.80	0.9988	0.9848

Myristoylated alanine-rich C-kinase substrate	31.54	4.45	0.9540	0.9849
Isoform 1 of Short transient receptor potential channel 6	106.26	6.67	0.8813	0.9849
Hemoglobin subunit alpha	15.25	8.68	0.9498	0.9851
Copine-3	60.09	5.85	1.0657	0.9851
Protein FAM126B	58.61	7.90	0.8012	0.9851
UPF0587 protein C1orf123	18.04	5.01	0.9045	0.9852
cDNA FLJ53094, highly similar to Receptor expression-enhancing protein 5	17.75	8.65	0.9768	0.9853
60S ribosomal protein L6	32.71	10.58	1.0411	0.9855
cDNA FLJ25361 fis, clone TST01713	28.99	6.64	1.1984	0.9856
MRG-binding protein	22.40	5.83	1.0316	0.9856
Isoform 4 of Acyl-CoA dehydrogenase family member 10	32.18	9.09	0.9316	0.9857
Isoform PPEF-2(L) of Serine/threonine-protein phosphatase with EF-hands 2	86.38	7.05	1.2219	0.9857
Isoform 1 of Otogelin	314.59	5.91	0.9635	0.9857
hypothetical protein	29.69	8.31	0.9797	0.9859
Ribosomal protein L15	24.16	11.62	1.0788	0.9861
Actin-related protein 3	47.34	5.88	0.9820	0.9861
165 kDa protein	164.99	8.25	0.9334	0.9862
Adenosylhomocysteinase	47.69	6.34	0.9474	0.9862
Isoform 1 of Abhydrolase domain-containing protein FAM108C1	35.81	5.64	0.9185	0.9862
40S ribosomal protein S7	22.11	10.10	0.9779	0.9862
proteasome 26S ATPase subunit 6	45.77	7.78	1.1133	0.9863
60S ribosomal protein L7a	29.98	10.61	0.9330	0.9864
12 kDa protein	11.90	7.30	0.6290	0.9866
centrosome spindle pole associated protein 1 isoform a	145.43	6.80	0.9879	0.9866
Desmoglein-3	107.44	5.00	0.9502	0.9867
Nucleus accumbens-associated protein 1	57.22	5.74	1.3142	0.9869
10 kDa protein	10.24	8.10	0.9278	0.9870
Zinc finger protein 266	62.08	8.59	0.9998	0.9870
cDNA FLJ55482, highly similar to Annexin A11	65.55	8.27	1.1892	0.9871
Isoform Long of Inositol 1,4,5-trisphosphate receptor type 2	307.88	6.46	1.1654	0.9871
Putative uncharacterized protein SLAIN2 (Fragment)	32.05	10.87	0.8720	0.9872
similar to hCG1793014	42.64	9.03	0.5780	0.9872
Isoform 1 of Otoferlin	226.61	5.69	1.1467	0.9873
cDNA FLJ78679, highly similar to Homo sapiens DEAD (Asp-Glu-Ala-Asp) box polypeptide 46 (DDX46), mRNA	117.40	9.29	1.1020	0.9873
cDNA FLJ50573, highly similar to Homo sapiens NAD(P)H dehydrogenase, quinone 1 (NQO1), transcript variant 3, mRNA	22.78	8.50	0.8170	0.9873
Similar to Tubulin beta chain	25.26	4.82	1.0241	0.9873
14 kDa protein	14.47	9.07	1.0045	0.9873
Eukaryotic translation initiation factor 3 subunit K	25.04	4.93	1.0246	0.9874
Acidic leucine-rich nuclear phosphoprotein 32 family member A	28.57	4.09	0.8246	0.9876
11 kDa protein	10.50	9.69	0.7541	0.9876
Isoform 1 of Heat shock cognate 71 kDa protein	70.85	5.52	1.0259	0.9879
Eukaryotic translation initiation factor 3 subunit A	166.47	6.79	1.1010	0.9879
cDNA FLJ57599, moderately similar to Eukaryotic translation initiation factor 3 subunit 1	22.93	4.83	1.0538	0.9881
Isoform 2 of GC-rich sequence DNA-binding factor	84.79	6.20	1.0159	0.9881
Isoform 3 of 250 kDa substrate of Akt	206.31	6.28	0.8705	0.9881
Protein	25.46	6.86	1.0536	0.9882
Transgelin-2	22.38	8.25	1.0795	0.9883
Transcription elongation factor B (SIII), polypeptide 2 (18kDa, elongin B), isoform CRA_b	12.52	4.97	1.0425	0.9883
183 kDa protein	182.89	6.70	0.8952	0.9883
Isoform 1 of TNFAIP3-interacting protein 2	48.67	6.44	1.0005	0.9884
Isoform 2 of Polyadenylate-binding protein 4	69.53	9.52	1.0222	0.9885
cDNA FLJ14048 fis, clone HEMBA1006650, weakly similar to ARP2/3 COMPLEX 20 KD SUBUNIT	16.96	8.81	0.8604	0.9886
cDNA FLJ57766, moderately similar to Eukaryotic initiation factor 4A-I	15.89	4.88	1.3100	0.9886

1 kDa protein	1.20	4.50		0.9887
Isoform 3 of WD repeat-containing protein 16	60.92	6.83	0.9383	0.9889
DNA replication licensing factor MCM6	92.83	5.41	0.9841	0.9890
Eukaryotic translation initiation factor 6	11.82	6.27	0.9454	0.9891
Complement component 1 Q subcomponent-binding protein, mitochondrial	31.34	4.84	0.8046	0.9891
60S ribosomal protein L28	15.74	12.02	0.9106	0.9892
Isoform ASF-1 of Splicing factor, arginine/serine-rich 1	27.73	10.36	0.8456	0.9894
cDNA FLJ33169 fis, clone ADRGL2000384, highly similar to Serine/threonine-protein phosphatase 2A 65 kDa regulatory subunit A alpha isoform	45.57	5.36	0.9327	0.9894
Isoform 2 of Protein CEI	24.36	12.03	1.0348	0.9895
Isoform 2 of Ryanodine receptor 1	564.33	5.30	1.1811	0.9896
Isoform 4 of RNA-binding protein 10	94.31	6.73	1.0141	0.9897
Isoform 1 of Sodium channel protein type 8 subunit alpha	225.13	6.28	0.9047	0.9898
cDNA, FLJ79369, highly similar to JmjC domain-containing histone demethylation protein 1B	87.94	9.11	0.9951	0.9900
Calcium-activated chloride channel regulator 2	103.88	7.02	0.9152	0.9901
Isoform 4 of RING finger protein 17	179.39	5.41		0.9901
p180/ribosome receptor	165.62	8.95	1.0040	0.9902
Isoform 2 of Cytoplasmic dynein 2 heavy chain 1	493.08	6.54	0.9548	0.9905
Putative uncharacterized protein ENSP00000354291	52.35	8.12	1.0077	0.9908
Isoform 2 of Cytoskeleton-associated protein 4	58.16	5.29	1.1628	0.9909
Isoform 2 of Plexin-D1	195.52	7.27	1.3133	0.9909
Isoform 2 of Thyroglobulin	298.19	5.63	1.0522	0.9910
Isoform 2 of Olfactomedin-like protein 2B	27.54	6.29	1.1196	0.9910
Eukaryotic initiation factor 4A-I	46.12	5.48	1.0068	0.9911
Alpha-actinin-2	103.79	5.45	0.9034	0.9913
Isoform 1 of Fumarylacetoacetate hydrolase domain-containing protein 1	24.83	7.39	1.2441	0.9915
9 kDa protein	8.61	11.94	1.0972	0.9915
33 kDa protein	33.16	5.29	0.9266	0.9916
Ornithine aminotransferase, mitochondrial	48.50	7.03	0.9457	0.9916
RING1 and YY1-binding protein	24.81	9.63	0.8521	0.9917
Protocadherin 9	113.64	5.41	0.9264	0.9918
Retinal guanylyl cyclase 1	119.98	7.44	0.5108	0.9920
Coactosin-like protein	15.94	5.67	0.9541	0.9921
Putative uncharacterized protein LOC389592	87.81	5.24	0.9609	0.9923
Putative stereocilin-like protein	192.31	5.40	1.1714	0.9925
BTB/POZ domain-containing protein TNFAIP1	36.18	8.03	0.9542	0.9925
Isoform 1 of Uncharacterized protein KIAA1751	86.90	5.52	1.0234	0.9926
Bone morphogenetic protein receptor, type II (Serine/threonine kinase), isoform CRA_a	59.92	5.66	1.0097	0.9928
Aspartate aminotransferase, cytoplasmic	46.22	7.01	0.8579	0.9929
Isoform 2 of UPF0378 protein KIAA0100	250.05	7.11	1.0134	0.9930
trans-2-enoyl-CoA reductase, mitochondrial isoform b	32.21	7.21	0.9932	0.9931
transforming growth factor, beta receptor III	93.44	5.71	1.1444	0.9931
Hemochromatosis protein (Fragment)	40.00	9.31	0.9685	0.9932
Isoform 3 of Alpha-catulin	72.57	7.06	1.1760	0.9933
Isoform 2 of DNA-binding protein RFX7	137.78	7.69	1.0680	0.9935
Isoform 1 of SLIT and NTRK-like protein 6	95.05	6.52	0.9430	0.9935
26S protease regulatory subunit 7	48.60	5.95	1.0424	0.9936
FAST kinase domain-containing protein 5	86.52	8.13	0.8730	0.9936
Isoform 3 of Spectrin beta chain, erythrocyte	242.65	5.35	1.0743	0.9938
cDNA FLJ37673 fis, clone BRHIP2012141	15.81	8.88	1.0320	0.9938
Protein	32.16	9.82	0.8226	0.9939
Eukaryotic translation initiation factor 2 subunit 1	36.09	5.08	1.0458	0.9941
Isoform 2 of Filamin-B	275.53	5.81	1.0086	0.9942
Isoform Beta of Tripartite motif-containing protein 29	63.80	6.98	1.0129	0.9944
Fructose-bisphosphate aldolase A	39.40	8.09	0.9355	0.9945

Methionine aminopeptidase	48.32	6.87	1.1174	0.9946
cDNA FLJ51165, highly similar to DNA damage-binding protein 1	91.93	5.30	0.9075	0.9947
Cysteine and glycine-rich protein 1	20.55	8.57	1.3439	0.9947
79 kDa protein	78.58	6.61	1.0498	0.9947
Probable G-protein coupled receptor 149	80.93	6.98	1.1265	0.9948
Leucine-rich repeat-containing protein 8C	92.33	7.72	1.0627	0.9949
18 kDa protein	18.04	5.30	1.0389	0.9949
Putative uncharacterized protein ZNF713 (Fragment)	30.87	7.68	1.0749	0.9950
Elongation factor 1-beta	24.75	4.67	0.9495	0.9950
Actin-related protein 2/3 complex subunit 1A	41.54	8.18	0.9332	0.9950
Isoform 1 of Protein ALO17	575.50	6.49	0.7371	0.9951
Conserved hypothetical protein	11.91	9.99	0.9640	0.9951
Laminin subunit beta-2	195.85	6.52	0.8684	0.9952
18 kDa protein	17.67	5.40	0.9275	0.9954
cDNA FLJ56092, highly similar to Pentatricopeptide repeat protein 1	84.06	8.57	1.1009	0.9956
F-box and leucine-rich repeat protein 13, isoform CRA_b	94.32	8.56	0.8606	0.9957
Single-stranded DNA-binding protein, mitochondrial	17.25	9.60	0.8672	0.9957
FK506-binding protein 3	25.16	9.28	1.0019	0.9957
cDNA FLJ56420, highly similar to Aspartyl aminopeptidase	54.46	7.72	1.0610	0.9957
V-type proton ATPase subunit D	28.25	9.36	0.8993	0.9958
60S ribosomal protein L27	15.79	10.56	0.9749	0.9958
PKDREJ (Fragment)	255.51	9.11	1.0977	0.9958
cDNA FLJ52068, highly similar to Microtubule-associated protein RP/EB family member 1	26.57	5.29	1.0337	0.9959
Proliferating cell nuclear antigen	28.75	4.69	0.8664	0.9959
Cytochrome c oxidase subunit 4 isoform 1, mitochondrial	19.56	9.51	0.9589	0.9960
metallophosphoesterase domain containing 2 isoform 2	31.49	7.34	0.9471	0.9960
Isoform 2 of Probable E3 ubiquitin-protein ligase MYCBP2	509.38	7.03	1.1657	0.9961
High mobility group protein B2	24.02	7.81	0.8386	0.9962
Isoform 2 of Polyadenylate-binding protein 1	61.14	9.07	1.0528	0.9963
Isoform A of Probable cation-transporting ATPase 13A1	132.87	8.13	0.8076	0.9963
vacuolar protein sorting 13C protein isoform 1B	402.83	6.38	0.9192	0.9963
Transmembrane protein 52	10.63	10.08	0.9728	0.9964
functional smad suppressing element 18	117.34	6.76	0.9881	0.9965
cDNA FLJ55925, highly similar to Homo sapiens murine retrovirus integration site 1 homolog (MRVI1), transcript variant 1, mRNA	98.64	5.80	1.1053	0.9965
hypothetical protein, partial	19.16	13.03	0.9383	0.9965
Isoform Non-muscle of Myosin light polypeptide 6	16.92	4.65	1.1414	0.9966
Cyclin-T1	80.63	8.78	1.0628	0.9966
Isoform 2 of 40S ribosomal protein S24	15.06	10.89	0.8609	0.9969
ATP synthase subunit beta, mitochondrial	56.52	5.40	0.8540	0.9969
Isoform 2 of BolA-like protein 2	6.84	5.85	0.9767	0.9970
Putative uncharacterized protein CAPZA1 (Fragment)	31.75	6.19	0.8355	0.9971
cDNA FLJ56307, highly similar to Ubiquitin thioesterase protein OTUB1	35.16	5.59	1.1025	0.9971
24 kDa protein	23.61	9.51	1.2015	0.9972
ATP synthase subunit O, mitochondrial	23.26	9.96	0.9352	0.9972
similar to hCG2045270	9.00	7.21	1.1920	0.9972
Synaptonemal complex protein 2	175.53	8.85	1.0657	0.9973
cDNA FLJ56579, highly similar to Prohibitin-2	29.03	10.02	0.9873	0.9975
MCM10 minichromosome maintenance deficient 10 (S. cerevisiae), isoform CRA_b	96.06	8.73	1.0758	0.9975
Immunodeficiency virus type I enhancer binding protein 1	30.93	6.21	1.2419	0.9975
cDNA FLJ53068, highly similar to Adenylyl cyclase-associated protein 1	47.09	8.28	1.0886	0.9975
Translationally-controlled tumor protein	19.58	4.93	0.8844	0.9975
cDNA FLJ55454, moderately similar to Oxygen-regulated protein 1	102.54	7.80	1.0013	0.9976
RNA polymerase II elongation factor ELL2	72.31	9.00	0.9729	0.9976
phosphatidylinositol glycan anchor biosynthesis, class Z	63.43	8.34	0.7328	0.9976
HLA complex group 4 protein	35.79	10.71	0.9401	0.9977

Isoform 4 of Plasminogen activator inhibitor 1 RNA-binding protein	42.40	8.44	0.8715	0.9977
Talin-2	271.39	5.59	1.3027	0.9978
eukaryotic translation elongation factor 1 delta isoform 1	71.36	6.42	0.9295	0.9979
L-aminoadipate-semialdehyde dehydrogenase-phosphopantetheinyl transferase	35.75	6.80	0.9014	0.9979
Ras-related protein Rab-7a	23.47	6.70	1.0332	0.9980
Nuclear fragile X mental retardation-interacting protein 1	56.26	9.13	0.7160	0.9980
40S ribosomal protein S11	18.42	10.30	0.9616	0.9981
Isoform RTN1-A of Reticulon-1	83.57	4.69	1.0663	0.9983
Isoform 6 of Sialic acid-binding Ig-like lectin 10	60.30	9.33	0.8688	0.9983
Isoform 3 of Dedicator of cytokinesis protein 9	239.13	7.80	1.0708	0.9984
Alpha-actinin-4	104.79	5.44	1.0284	0.9984
Voltage-dependent anion-selective channel protein 1	30.75	8.54	0.9138	0.9986
Putative uncharacterized protein COL5A1	23.97	5.95	1.0114	0.9986
Putative uncharacterized protein ITIH3	75.03	5.86	1.0284	0.9986
29 kDa protein	29.28	8.91	0.9653	0.9986
Enhancer of rudimentary homolog	12.25	5.92	1.4829	0.9987
importin 5	125.46	4.92	0.9752	0.9988
cDNA FLJ60299, highly similar to Rab GDP dissociation inhibitor beta	51.12	8.18	0.9506	0.9988
cDNA FLJ58466, highly similar to Leucyl-tRNA synthetase, cytoplasmic	129.10	7.78	1.1133	0.9989
CDGSH iron sulfur domain-containing protein 2	15.27	9.61	1.0179	0.9991
Tripartite motif-containing protein 32	71.94	6.98	0.7793	0.9991
Isoform 2 of Vam6/Vps39-like protein	100.70	7.12	1.1076	0.9992
Proteasome 26S non-ATPase subunit 11 variant (Fragment)	47.51	6.48	1.0448	0.9992
Isoform 4 of Homer protein homolog 2	18.92	9.47	0.9525	0.9993
226 kDa protein	226.03	5.92	1.3979	0.9994
Nucleolin	76.57	4.70	0.8642	0.9996
Isoform 1 of Dynein heavy chain 7, axonemal	460.85	6.00	0.9033	0.9996
Isoform 2 of Bardet-Biedl syndrome 7 protein	75.40	6.11	1.3074	0.9997
cDNA FLJ57390	51.47	8.27	0.8858	0.9997
calpastatin isoform f	80.22	5.15	0.8780	0.9997
cDNA FLJ60201, highly similar to Matrix metalloproteinase-19	33.77	5.40	1.1901	1.0002
Isoform S of Kinesin light chain 1	62.79	5.94	1.0814	1.0003
cDNA FLJ60952, highly similar to HRAS-like suppressor 5	27.63	8.06	1.0266	1.0003
cDNA FLJ50714, moderately similar to Ras-related protein Rap-1b	15.35	8.13	0.9973	1.0003
Coiled-coil domain-containing protein KIAA1407	110.50	9.60	0.7901	1.0003
ATP-binding cassette sub-family A member 1	254.12	6.86	0.9351	1.0004
Stress-induced-phosphoprotein 1	62.60	6.80	0.9206	1.0004
Isoform B of Pituitary-specific positive transcription factor 1	32.89	8.12	0.8781	1.0005
Isoform 1 of RAS and EF-hand domain-containing protein	82.83	5.11	1.0812	1.0005
36 kDa protein	35.72	6.40	1.2401	1.0006
Ras-related protein Rab-22A	21.84	8.15	0.9723	1.0006
Tetratricopeptide repeat protein 28	231.80	6.70	0.8810	1.0006
Putative uncharacterized protein PRDX5 (Fragment)	16.87	7.24	0.9867	1.0006
Probable methyltransferase BCDIN3D	33.18	6.70	1.0042	1.0008
13 kDa protein	13.31	4.70	0.8296	1.0009
Reticulocalbin-1	38.87	5.00	0.9594	1.0009
Putative uncharacterized protein GPR179	257.29	5.71	0.9983	1.0009
Cytoplasmic dynein 1 heavy chain 1	532.07	6.40	1.0245	1.0009
Isoform Short of Ubiquitin carboxyl-terminal hydrolase 5	93.25	5.08	1.0324	1.0011
cDNA: FLJ22686 fis, clone HSI10987	55.04	8.02	1.1854	1.0011
Transcription elongation factor SPT4	13.18	8.06	1.0594	1.0012
Isoform 2 of Forkhead box protein M1	82.63	8.35	0.9888	1.0012
Putative uncharacterized protein DKFZp686G1675 (Fragment)	45.41	8.18	0.9372	1.0012
Prohibitin	29.79	5.76	0.9263	1.0015
Peptidyl-prolyl cis-trans isomerase	38.28	8.21	0.8247	1.0015
Mitochondrial fission 1 protein	16.93	8.79	0.9383	1.0016

Isoform 1 of Protein KIAA1881	134.35	8.73	0.8788	1.0016
Guanine nucleotide-binding protein G(I)/G(S)/G(O) subunit gamma-5	7.31	9.85	0.9963	1.0018
Dolichyl-diphosphooligosaccharide--protein glycosyltransferase subunit 1 precursor	72.73	6.28	1.0784	1.0019
Elongation factor 2	95.28	6.83	1.0047	1.0022
Isoform 1 of Pleckstrin homology domain-containing family A member 4	85.35	10.56	0.8932	1.0024
60S ribosomal protein L3	46.08	10.18	0.9551	1.0026
Isoform 2 of Mitochondrial import inner membrane translocase subunit TIM50	50.43	9.42	1.0697	1.0027
cDNA FLJ41858 fis, clone NT2RI3006796	13.00	10.04	1.1731	1.0027
WD repeat protein	62.30	5.66	0.9782	1.0027
ADP-ribosylation factor 4	20.50	7.14	0.9648	1.0028
Mitochondrial import inner membrane translocase subunit TIM44	51.32	8.32	0.8959	1.0029
Thioredoxin domain-containing protein 17	13.93	5.52	0.8966	1.0030
Tubulin beta chain	49.64	4.89	0.9392	1.0030
49 kDa protein	48.57	8.41	0.8718	1.0030
Isoform ARPP-16 of cAMP-regulated phosphoprotein 19	10.63	9.70	0.6724	1.0031
23 kDa protein	23.23	5.68	0.8430	1.0032
MutL protein homolog 1 variant (Fragment)	84.46	5.72	0.8041	1.0033
5 kDa protein	5.11	10.01	0.7735	1.0035
Isoform 2 of Hypermethylated in cancer 1 protein	74.43	6.99	0.9622	1.0036
Coiled-coil domain-containing protein 6	65.88	7.18	0.9726	1.0037
Isoform 4 of Nesprin-1	1004.57	5.52	0.9970	1.0038
40S ribosomal protein S20	13.36	9.94	1.0504	1.0038
40S ribosomal protein S12	14.51	7.21	0.9737	1.0039
Isoform 1 of Calcyclin-binding protein	26.19	8.25	1.1430	1.0039
tumor protein p53 binding protein 1 isoform 2	208.89	4.67	0.8810	1.0040
Trafficking kinesin-binding protein 2	101.36	5.24	0.9091	1.0040
Glucose-6-phosphate isomerase	63.11	8.32	1.0538	1.0042
Isoform 2 of Filamin-A	279.84	6.05	0.9528	1.0042
neurotrimin isoform 3	39.20	8.00	0.9661	1.0044
Novel protein similar to Pre-B cell enhancing factor	53.36	7.80	1.1523	1.0046
Isoform 1 of FK506-binding protein 8	44.53	4.84	1.0687	1.0047
cDNA FLJ51237, moderately similar to Carcinoembryonic antigen-related cell adhesion molecule 8	27.83	8.44	0.7750	1.0047
Thioredoxin domain containing 5 isoform 2	43.64	6.13	0.9077	1.0047
cDNA FLJ57640, highly similar to Serpin B5	32.44	6.54	1.1667	1.0047
Isoform 1 of HEAT repeat-containing protein 7A	181.16	6.89	0.8541	1.0048
18 kDa protein	17.58	10.18	0.8551	1.0050
B-cell receptor-associated protein 31	27.97	8.44	0.9930	1.0050
Transcription initiation factor TFIID 210 kDa subunit	207.17	5.40	0.9212	1.0050
OGT(O-Glc-NAc transferase)-interacting protein 106 KDa isoform 2	77.21	4.86	1.0535	1.0050
Actin-related protein 2/3 complex subunit 2	34.31	7.36	1.0360	1.0052
Vitronectin	54.27	5.80	0.5520	1.0054
FOXP4 protein (Fragment)	42.06	8.69	1.6021	1.0056
annexin VI isoform 2	75.23	5.67	1.1414	1.0056
Mitochondrial import inner membrane translocase subunit Tim9 B	11.58	7.43	0.9315	1.0057
Neutral amino acid transporter B(0)	56.56	5.48	1.0514	1.0057
Protein disulfide-isomerase A3	56.75	6.35	0.8751	1.0058
40S ribosomal protein S28	7.84	10.70	0.9485	1.0059
66 kDa protein	65.99	8.06	0.8978	1.0060
Protein	131.12	6.28	0.9327	1.0061
Gamma-aminobutyric acid type B receptor subunit 2	105.75	8.66	1.2574	1.0061
Isoform 2 of Neurobeachin-like protein 2	282.68	6.54	1.0034	1.0062
snRNA-activating protein complex subunit 4	159.34	8.28	1.0082	1.0062
19 kDa protein	19.40	11.30	0.9194	1.0063
Negative elongation factor B	65.66	6.13	1.2717	1.0064
Isoform 4 of Catenin delta-1	65.72	8.00	0.9119	1.0065
Putative uncharacterized protein HIBCH (Fragment)	15.97	9.44	1.0810	1.0065

Conserved hypothetical protein	12.35	9.45	0.9907	1.0066
Huntingtin	347.64	6.20	1.1654	1.0066
Ventral anterior homeobox 2	30.86	9.47	1.0249	1.0067
similar to mCG50504	19.94	9.60	0.9127	1.0068
cDNA FLJ52487, highly similar to LIM domain-binding protein 2	21.43	10.43	1.1402	1.0069
FLJ00404 protein (Fragment)	27.85	11.62	1.0337	1.0069
11 kDa protein	11.11	5.05	0.9706	1.0070
Isoform RMO1ab of Ras-associated and pleckstrin homology domains-containing protein 1	141.09	8.90	1.0164	1.0070
Thioredoxin, isoform CRA_b	9.45	6.04	0.7912	1.0071
Probable methyltransferase TARBP1	181.56	7.05	1.1006	1.0072
DNA polymerase theta	289.44	7.36	1.0950	1.0072
Similar to Glucagon-like peptide 1 receptor precursor	21.12	11.93	0.9039	1.0075
Adaptor-related protein complex 2, beta 1 subunit, isoform CRA_f	98.06	5.24	0.8593	1.0076
10 kDa protein	10.33	11.78	0.8525	1.0076
Protein	19.87	10.11	1.0603	1.0076
Isoform 3 of Cancer susceptibility candidate protein 1	79.32	5.59	0.9975	1.0078
Isoform 3 of Obscurin	721.10	5.74		1.0078
Breast cancer type 2 susceptibility protein	383.99	6.73	0.8982	1.0081
Germ cell-less protein-like 1	58.65	7.44	0.9526	1.0083
16 kDa protein	16.08	9.04	1.0059	1.0086
myosin IXB isoform 2	229.00	8.56	1.2039	1.0087
Similar to Zinc finger with KRAB and SCAN domains 2	62.42	7.85	1.0983	1.0088
Isoform 1 of Protein disulfide-isomerase A6	48.09	5.08	0.9302	1.0089
cDNA FLJ59405, highly similar to Eukaryotic translation initiation factor 4B	64.77	6.13	0.7484	1.0089
Isoform 6 of Interleukin enhancer-binding factor 3	76.45	7.91	1.0445	1.0089
ATP synthase subunit g, mitochondrial	11.42	9.64	0.8454	1.0090
Glutathione S-transferase omega 1	23.33	7.21	1.0022	1.0091
Zyxin	67.24	7.23	1.0732	1.0091
cDNA FLJ58480, highly similar to Protocadherin beta 14	60.82	4.86	1.0781	1.0091
Isoform 1 of Phosphoglucomutase-1	61.41	6.76	1.1146	1.0092
Putative uncharacterized protein ANKRD43	35.37	10.98	1.0531	1.0092
T-complex protein 1 subunit epsilon	59.63	5.66	0.9286	1.0092
Isoform 2 of Cytoskeleton-associated protein 5	218.39	8.06	1.1253	1.0093
cDNA FLJ58737, highly similar to Splicing factor 3A subunit 3	52.40	5.27	0.9574	1.0094
similar to DNA dependent protein kinase catalytic subunit	465.17	7.23	1.0625	1.0096
Isoform 2 of Probable bifunctional methylenetetrahydrofolate dehydrogenase/cyclohydrolase 2	7.34	9.36	0.8362	1.0096
Isoform 1 of Protocadherin gamma-A5	100.87	4.87	1.0416	1.0097
CTP synthase 1	66.65	6.46	1.1562	1.0098
cDNA FLJ55997, highly similar to Copine-6	67.57	5.91	1.0207	1.0100
Isoform 2 of Calcium-binding mitochondrial carrier protein SCaMC-1	51.32	5.88	0.9251	1.0102
similar to hCG2010820	125.85	8.40	0.8533	1.0103
Translocon-associated protein subunit delta precursor	20.20	5.81	0.9923	1.0103
cDNA FLJ55480, highly similar to Exportin-1	123.22	6.06	1.0006	1.0104
Hsc70-interacting protein	41.31	5.27	0.8635	1.0106
Heat shock 70 kDa protein 4	94.27	5.19	0.9796	1.0106
Isoform 2 of Sperm flagellar protein 1	18.42	9.14	0.9902	1.0107
Serine/threonine-protein phosphatase PP1-alpha catalytic subunit	37.49	6.33	1.0542	1.0107
Protein unc-13 homolog C	250.75	5.92	0.9334	1.0107
Histidine ammonia-lyase	72.65	6.95	1.0068	1.0108
Isoform 2 of Fatty acid desaturase 6	38.37	8.90	0.9865	1.0110
similar to barrier-to-autointegration factor	9.73	6.09	1.1593	1.0111
cDNA FLJ60097, highly similar to Tubulin alpha-ubiquitous chain	46.28	5.08	0.9300	1.0111
Protein disulfide-isomerase	57.08	4.87	0.8686	1.0111
Isoform 3 of Ninein	245.98	5.03	0.8212	1.0112
cDNA FLJ61500, highly similar to NNP-1 protein	46.12	6.80	0.9764	1.0113

Isoform Non-brain of Clathrin light chain B	23.17	4.69	1.0902	1.0115
cDNA FLJ57755, highly similar to Homo sapiens interleukin 17 receptor E (IL17RE), transcript variant 5, mRNA	21.22	7.81	1.2264	1.0115
Cofilin-1	18.49	8.09	0.9628	1.0115
Isoform 1 of NLR family member X1	107.55	7.37	0.9866	1.0116
Putative uncharacterized protein MYO7B	267.41	8.65	0.9740	1.0118
Isoform 2 of Protein KIAA1199	110.40	6.89	0.9536	1.0119
Isoform 1 of Netrin receptor UNC5A	92.90	6.79	1.2025	1.0120
T-complex protein 1 subunit beta	57.45	6.46	0.9726	1.0121
Protein	18.31	6.19	0.9077	1.0122
Ras GTPase-activating protein-binding protein 1	52.13	5.52	1.0787	1.0122
Putative uncharacterized protein PDXK	30.62	6.65	0.8605	1.0123
Isoform 4 of Uncharacterized protein C17orf97	46.40	8.50	1.0178	1.0124
Putative uncharacterized protein DKFZp686B16128	89.73	9.33	1.5888	1.0124
Isoform 2 of Cell division control protein 42 homolog	21.25	6.55	1.0017	1.0124
TNC protein	210.35	4.98	0.6334	1.0126
Putative uncharacterized protein C20orf66	39.47	4.94	0.9461	1.0126
Angiopoietin-related protein 6	51.66	8.53	1.3770	1.0127
Protein phosphatase 2A activator, regulatory subunit 4	7.40	8.47	0.9853	1.0130
LysM and putative peptidoglycan-binding domain-containing protein 4	32.05	6.44	1.0873	1.0130
Isoform 1 of Signal transducing adapter molecule 2	58.13	5.07	1.0188	1.0132
Myosin regulatory light chain MRLC3	19.78	4.81	0.9289	1.0132
cDNA FLJ54122, highly similar to Cytosol aminopeptidase	39.32	9.31	0.8580	1.0132
Putative uncharacterized protein ENSP00000374919	18.03	8.12	1.0757	1.0133
Protein salvador homolog 1	44.61	9.09	0.9866	1.0134
11 kDa protein	11.22	6.15	1.1958	1.0138
Isoform 1 of Chromodomain-helicase-DNA-binding protein 7	335.72	6.34	0.8412	1.0138
60S ribosomal protein L37	11.07	11.74	0.8076	1.0139
Protein-glutamine gamma-glutamyltransferase Z	79.89	7.01	1.0015	1.0139
similar to 40s ribosomal protein S27	9.53	8.72	0.8018	1.0139
Isoform HMG-I of High mobility group protein HMG-I/HMG-Y	11.67	10.32	0.8452	1.0140
cDNA FLJ50688, highly similar to Casein kinase II subunit beta	12.77	4.21	0.9221	1.0141
Isoform 4 of CCR4-NOT transcription complex subunit 1	173.68	7.34	1.1878	1.0143
Isoform 2 of Uncharacterized protein C17orf85	38.90	7.33	1.1523	1.0143
Elastase-1	27.78	8.29	1.0727	1.0143
Methyltransferase-like protein 7A	28.30	8.38	0.9361	1.0145
similar to hCG1645245	27.30	9.83	0.7917	1.0145
Mitochondrial ribosomal protein L49 variant (Fragment)	16.05	9.70	0.8830	1.0147
Isoform 2 of Adenylyl cyclase-associated protein 1	51.75	8.10	1.0759	1.0148
Tubulin-specific chaperone A	12.85	5.29	0.7247	1.0148
Isoform 2 of Eukaryotic translation initiation factor 2 subunit 3-like protein	50.63	7.66	1.0413	1.0149
HRB2 variant B	36.65	9.61	0.8070	1.0152
cDNA FLJ43276 fis, clone KIDNE2011532, moderately similar to Homo sapiens melanoma-associated chondroitin sulfate proteoglycan 4	20.98	5.03	0.9680	1.0153
alpha 3 type VI collagen isoform 4 precursor	321.99	6.90	0.9940	1.0154
proteasome activator subunit 2	27.38	5.73	1.1523	1.0154
Coiled-coil domain-containing protein 121	33.04	9.83	0.8574	1.0156
Isoform 1 of DENN domain-containing protein 4C	186.74	6.86	0.8937	1.0160
Protein	15.77	10.52	0.9924	1.0161
Aspartate aminotransferase, mitochondrial	47.45	9.01	0.9286	1.0161
Isoform Beta of ADAM 30	87.92	7.61	0.7673	1.0162
Isoform 2 of Angiopoietin-2	50.93	6.06	0.9431	1.0162
cDNA FLJ60077, highly similar to Sodium/potassium-transporting ATPase alpha-1 chain (Fragment)	112.37	5.35	0.9444	1.0163
T-complex protein 1 subunit eta	59.33	7.65	1.0014	1.0163
Matrin-3	94.56	6.25	1.1278	1.0163
Isoform 2 of Caprin-1	76.81	5.12	0.8525	1.0164

Isoform 3 of EH domain-binding protein 1	132.24	5.85	0.9587	1.0165
cDNA FLJ52569, highly similar to Collagen-binding protein 2	44.18	8.95	1.0265	1.0166
Calreticulin	48.11	4.44	0.7730	1.0169
Ubiquitin-like domain-containing CTD phosphatase 1	36.78	6.46	0.9814	1.0170
cDNA FLJ57136, highly similar to RNA U small nuclear RNA export adapter protein	28.15	9.20	0.9159	1.0171
Isoform Heart of ATP synthase subunit gamma, mitochondrial	32.86	9.31	1.0232	1.0172
Isoform 1 of Uncharacterized protein C10orf118	103.62	6.27	1.0482	1.0172
L-lactate dehydrogenase B chain	36.62	6.05	0.9798	1.0173
Isoform 2 of TIP41-like protein	20.20	5.87	1.0386	1.0173
ATP synthase-coupling factor 6, mitochondrial	12.58	9.52	0.9792	1.0174
4F2 cell-surface antigen heavy chain	57.91	5.35	0.8762	1.0175
Pre-mRNA-processing-splicing factor 8	273.43	8.84	0.9445	1.0175
Isoform 1 of Mitogen-activated protein kinase kinase kinase 15	147.34	5.63	0.8862	1.0175
Phosphoglycerate dehydrogenase	53.05	6.92	1.0094	1.0176
Neuroblastoma breakpoint family member 6-like protein	27.61	6.93	1.3083	1.0176
PDZ and LIM domain protein 5	63.96	8.21	1.0887	1.0176
40S ribosomal protein S3	26.67	9.66	1.0154	1.0177
Isoform Short of FAS-associated factor 1	56.90	5.06	0.9096	1.0177
Alpha-actinin-1	102.99	5.41	1.0606	1.0177
60S ribosomal protein L29	17.74	11.66	0.8487	1.0178
Isoform 1 of Cullin-associated NEDD8-dissociated protein 1	136.29	5.78	1.0190	1.0179
Isoform alpha-enolase of Alpha-enolase	47.14	7.39	1.0216	1.0179
60S ribosomal protein L13a	23.56	10.93	1.0250	1.0180
Isoform 3 of Exportin-2	107.71	5.82	1.0438	1.0181
Puromycin-sensitive aminopeptidase	103.21	5.72	1.0487	1.0182
zinc finger protein 783	70.04	8.84	0.7375	1.0182
Interleukin enhancer-binding factor 2	43.04	5.26	1.0886	1.0183
Methylmalonyl-CoA mutase, mitochondrial	83.07	6.93	1.0013	1.0183
Isoform 3 of Mediator of RNA polymerase II transcription subunit 15	75.88	9.29	1.1376	1.0184
casein kinase 1, gamma 3 isoform 3	47.88	9.22	1.1797	1.0184
ATP synthase subunit alpha, mitochondrial	59.71	9.13	0.9347	1.0185
Protein disulfide-isomerase A4	72.89	5.07	0.8307	1.0187
Thyroid receptor-interacting protein 11	227.50	5.27	1.1265	1.0188
Adenylate cyclase type 1	123.36	8.43	1.0093	1.0189
AMP-activated protein kinase, noncatalytic gamma-1 subunit isoform 2	28.27	7.02	1.0180	1.0192
ring finger protein 7 isoform 3	9.64	5.38	0.9537	1.0192
Isoform 1 of Nucleophosmin	32.55	4.78	0.8636	1.0195
Isoform 2 of Myosin-IXa	290.27	8.90	0.9996	1.0195
Prefoldin subunit 2	16.64	6.58	0.9974	1.0197
HLA class II histocompatibility antigen, DQ(6) alpha chain	28.01	4.88	0.7865	1.0197
PR domain containing 16	138.09	6.27	1.0063	1.0198
cDNA FLJ55792, highly similar to L-lactate dehydrogenase A chain	39.81	8.43	1.0178	1.0198
Small nuclear ribonucleoprotein Sm D1	13.27	11.56	1.3404	1.0199
Isoform 6 of Microtubule-associated protein 4	119.90	6.23	0.9983	1.0199
Rac GTPase-activating protein 1	70.98	8.88	1.0097	1.0200
similar to hCG2040259	13.54	10.07	0.9178	1.0201
Similar to Elongation factor 1-alpha 1	50.12	8.95	1.1118	1.0201
ubiquitin-conjugating enzyme E2D 2 isoform 2	13.63	8.29	0.9518	1.0203
v-kit Hardy-Zuckerman 4 feline sarcoma viral oncogene homolog isoform 2 precursor	109.38	6.89	1.0615	1.0204
Proteinase-activated receptor 4	41.11	8.92	0.8986	1.0204
similar to Protein zyg-11 homolog A	83.02	8.38	0.9927	1.0205
Guanine nucleotide-binding protein subunit beta-2-like 1	35.05	7.69	0.9186	1.0206
cell division cycle 2 isoform 1	34.06	8.40	1.1115	1.0208
Major histocompatibility complex class II DP beta 1	28.38	8.03	1.0187	1.0208
Isoform 2 of 60S ribosomal protein L11	20.11	9.60	0.9563	1.0209
Stathmin	17.29	5.97	0.8755	1.0209

Cardiomyopathy-associated protein 5	448.94	4.78	0.9833	1.0211
Destrin	18.49	7.85	0.9933	1.0211
snRNA-activating protein complex subunit 2	35.53	6.01	1.6923	1.0211
48 kDa protein	47.99	10.56	0.9145	1.0212
Isoform 1 of Centlein	161.52	8.15	1.2822	1.0212
Isoform 1 of tRNA-specific adenosine deaminase 1	55.36	9.01	0.9071	1.0214
similar to putative chemokine receptor	28.75	7.08	0.9990	1.0215
zinc finger protein 534 isoform 1	75.74	9.35	0.9699	1.0216
Putative uncharacterized protein CALR3	21.38	5.19	1.3979	1.0217
Isoform 4 of Heterogeneous nuclear ribonucleoprotein Q	58.70	7.56	0.9208	1.0217
32 kDa protein	32.34	7.36	0.6160	1.0219
Peptidyl-prolyl cis-trans isomerase	10.11	5.90	1.0859	1.0219
Coatomer subunit gamma-2	97.56	5.81	1.1550	1.0220
mRNA turnover protein 4 homolog	27.54	8.29	1.1080	1.0221
Isoform Short of Autism susceptibility gene 2 protein	136.33	9.35	1.1051	1.0222
FLJ00003 protein (Fragment)	25.37	9.80	1.0115	1.0226
Putative uncharacterized protein EML4	18.36	9.42	1.0696	1.0228
Tetratricopeptide repeat protein 37	175.37	7.53	0.9556	1.0230
Isoform 1 of Polymerase I and transcript release factor	43.45	5.60	0.9692	1.0230
Plasminogen activator inhibitor 2	46.57	5.63	1.0582	1.0231
Parathymosin	11.52	4.16	0.8586	1.0232
DOCK3 protein	219.41	6.90	0.9587	1.0232
Isoform 2 of WD repeat-containing protein 1	57.97	6.89	0.9800	1.0232
Isoform GTBP-N of DNA mismatch repair protein Msh6	152.69	6.90	0.9876	1.0232
Inositol polyphosphate-5-phosphatase, 75kDa	34.57	5.47	1.8467	1.0232
Heterogeneous nuclear ribonucleoprotein F	45.64	5.58	0.8159	1.0232
57 kDa protein	57.23	9.23	0.9952	1.0232
Conserved hypothetical protein	9.44	9.13	0.9620	1.0233
UDP-glucose 6-dehydrogenase	54.99	7.12	0.9150	1.0234
abhydrolase domain containing 11 7	19.31	11.06	1.2000	1.0237
Isoform 2 of Proteasome subunit alpha type-3	27.63	5.33	0.8897	1.0237
plastin 3	70.77	5.60	1.0384	1.0238
24 kDa protein	24.20	8.65	1.0607	1.0238
Copine I	53.01	5.82	0.9740	1.0240
10 kDa protein	10.43	4.70	0.8691	1.0240
tRNA (cytosine-5-)-methyltransferase NSUN2	86.42	6.77	0.9343	1.0240
Isocitrate dehydrogenase [NADP] cytoplasmic	46.63	7.01	1.1370	1.0240
Ubiquitin-like modifier-activating enzyme 1	117.77	5.76	1.0368	1.0241
Keratin, type I cytoskeletal 16	51.24	5.05	0.8912	1.0243
L-myc-2 protein	41.09	5.47	0.9215	1.0244
Isoform 1 of Dual specificity protein phosphatase CDC14A	66.53	9.10	0.8943	1.0245
Methionine aminopeptidase 1	43.19	7.17	1.0937	1.0247
Isoform PIAS2-alpha of E3 SUMO-protein ligase PIAS2	63.33	8.15	1.3511	1.0247
Conserved hypothetical protein	22.19	11.66	0.9767	1.0249
cDNA FLJ56419, weakly similar to Homo sapiens patched domain containing 1 (PTCHD1), mRNA	96.31	8.54	0.9481	1.0254
Putative uncharacterized protein TRAPPC3	13.33	8.87	0.9605	1.0255
similar to histone	24.39	11.21	0.8981	1.0256
Isoform 1 of BTB/POZ domain-containing protein KCTD18	46.71	9.10	1.2629	1.0256
hypothetical protein LOC146556 isoform 2	44.60	6.84	0.8723	1.0256
similar to p41-Arc	40.79	8.85	1.3063	1.0257
Isoform 3 of Protein DGCR8	82.70	5.87	0.9786	1.0258
Isoform 1 of Glycogen debranching enzyme	174.65	6.76	1.0644	1.0258
cDNA FLJ35251 fis, clone PROST2003635, weakly similar to MULTIFUNCTIONAL AMINOACYL-TRNA SYNTHETASE	81.75	6.62	1.1279	1.0259
HSPC027	42.64	6.07	0.8789	1.0260
HELZ protein	218.98	7.42	0.9893	1.0261

Isoform 2 of Tubulointerstitial nephritis antigen	39.01	8.00	1.0395	1.0262
Thioredoxin domain-containing protein 12	19.19	5.40	1.0186	1.0263
Isoform 3 of Probable ATP-dependent RNA helicase DDX17	72.51	8.68	0.8879	1.0266
Gamma-soluble NSF attachment protein	34.72	5.41	0.8723	1.0267
Isoform 1 of 14-3-3 protein sigma	27.76	4.74	1.4064	1.0268
Erythrocyte band 7 integral membrane protein	31.71	7.88	1.0007	1.0268
Uncharacterized protein C16orf61	9.45	7.87	1.0590	1.0269
AN1-type zinc finger protein 5	23.12	8.51	1.1166	1.0269
84 kDa protein	83.78	8.98	1.0106	1.0273
Isoform 2 of Protein FAM5B	76.47	6.99	0.9007	1.0274
Isoform 1 of Extended synaptotagmin-1	122.78	5.83	1.0613	1.0274
Isoform Long of Retina-specific copper amine oxidase	83.62	7.03	0.9240	1.0275
Zinc finger protein 99	120.02	9.31	0.8156	1.0276
Isoform 1 of Nucleobindin-2	50.16	5.12	1.1796	1.0277
Small ubiquitin-related modifier 1	11.55	5.52	0.8779	1.0278
18 kDa protein	17.91	7.72	1.0857	1.0282
CYP2F1 protein	36.39	7.97	1.2517	1.0282
cDNA FLJ56291, highly similar to Synphilin-1	57.13	9.13	0.8854	1.0283
RCAN2 protein	25.06	6.89	0.9465	1.0283
Isoform 1 of Proline-rich transmembrane protein 3	102.13	7.78	0.8498	1.0284
Isoform 1 of Tumor protein D54	22.22	5.36	0.9824	1.0285
14-3-3 protein gamma	28.28	4.89	0.8180	1.0285
Glutathione S-transferase P	23.34	5.64	0.8649	1.0285
Transcription factor E2F8	94.11	8.98	1.2117	1.0286
Annexin A3	36.35	5.92	0.8842	1.0287
B aggressive lymphoma gene variant	80.24	8.06	0.9775	1.0287
PDCD6IP protein	96.76	6.52	1.1085	1.0287
cDNA: FLJ22037 fis, clone HEP08868 (Fragment)	86.94	5.35	0.8356	1.0288
Uncharacterized protein C5orf34	72.84	7.93	1.1248	1.0290
Isoform 1 of PDZ domain-containing protein 6	105.58	5.90	1.0779	1.0290
Calbindin	30.01	4.83	1.0785	1.0292
High-mobility group nucleosome binding domain 1	10.68	9.60	1.0138	1.0293
Transaldolase	37.52	6.81	0.8421	1.0294
Inward rectifier potassium channel 4	49.47	6.21	0.9293	1.0295
Isoform 1 of Transmembrane channel-like protein 3	125.79	9.22	0.8628	1.0297
Isoform 1 of Myosin-9	226.39	5.60	0.9806	1.0297
Alpha-1,6-mannosylglycoprotein 6-beta-N-acetylglucosaminyltransferase A	84.49	8.12	1.0440	1.0299
similar to hCG1778601 isoform 2	73.76	9.04	0.8167	1.0300
Putative uncharacterized protein GARS	77.48	6.24	1.0088	1.0300
eukaryotic translation initiation factor 4 gamma, 1 isoform 4	154.84	5.16	0.9948	1.0301
Coiled-coil domain-containing protein 86	40.21	10.33	1.1725	1.0301
Isoform 2 of Apolipoprotein A-I-binding protein	20.42	7.30	1.1739	1.0302
similar to hCG1997574	32.61	9.11	0.8215	1.0305
Isoform 3 of Uncharacterized protein C18orf2	9.40	7.40	1.2764	1.0306
Ras GTPase-activating-like protein IQGAP1	189.13	6.48	1.1074	1.0306
40S ribosomal protein S2	31.30	10.24	1.0676	1.0308
Isoform 3 of Heterogeneous nuclear ribonucleoprotein D-like	27.17	8.65	1.0694	1.0309
26S protease regulatory subunit 6A	49.17	5.24	1.0863	1.0309
ATP-dependent RNA helicase A	140.87	6.84	0.8377	1.0310
SHC-transforming protein 2	61.88	6.77	0.9271	1.0313
Neurexin-1-beta	46.60	7.80	1.1539	1.0315
Isoform 2 of Uncharacterized protein C4orf29	37.24	9.29	0.9846	1.0315
Bisphosphoglycerate mutase	29.99	6.54	1.1624	1.0317
Isoform 5 of E3 ubiquitin-protein ligase SHPRH	122.42	8.12	0.9319	1.0319
Isoform 2 of Stonin-2	79.47	7.01	1.0844	1.0320
Phosphofructokinase, platelet	85.26	8.59	0.9525	1.0321

Programmed cell death protein 5	14.28	6.04	1.0235	1.0322
Protein FAM44A	330.27	5.08	1.0027	1.0322
Tankyrase-2	126.84	7.20	0.9153	1.0323
Putative uncharacterized protein KIAA1244	232.67	5.69	1.0540	1.0324
cDNA FLJ58049, highly similar to RNA-binding protein FUS	44.79	9.00	0.7986	1.0324
Mesoderm development candidate 2	26.06	7.78	0.8813	1.0326
Putative RUNDC2-like protein 2	29.56	4.93	0.9829	1.0327
Isoform 2 of COP9 signalosome complex subunit 7b	18.15	7.34	0.8842	1.0328
Isoform 3 of Anoctamin-9	21.08	7.52	0.9824	1.0329
DNA polymerase	111.61	7.68	0.6585	1.0329
Keratin, type I cytoskeletal 14	51.59	5.16	1.1211	1.0330
cDNA FLJ14335 fis, clone PLACE4000411, highly similar to Homo sapiens mRNA	24.01	8.88	1.0668	1.0331
Isoform 1 of Uncharacterized protein C19orf57	84.18	5.08	1.2060	1.0332
Isoform 2 of RUN domain-containing protein 1	67.46	6.39	1.0379	1.0334
SUMO-conjugating enzyme UBC9	18.00	8.66	0.9978	1.0336
Isoform 3 of Protein GPR107	33.36	8.34	0.8435	1.0338
Transmembrane emp24 domain-containing protein 10	24.96	7.44	0.9127	1.0339
Tubby-related protein 4	169.07	7.62	0.8277	1.0340
Annexin A2	38.58	7.75	1.0589	1.0344
Conserved hypothetical protein	25.66	10.70	0.9480	1.0344
similar to hCG30646	30.90	9.77	1.1899	1.0346
Isoform 2 of Zinc finger FYVE domain-containing protein 26	282.38	6.40	0.9314	1.0349
cDNA FLJ55523, highly similar to Transcription initiation factor TFIID subunit 6	76.95	8.92	0.9347	1.0350
Isoform 1 of Leucine-rich repeat-containing protein 36	83.77	7.15	1.0102	1.0352
Isoform 2 of SH3 domain-containing kinase-binding protein 1	68.51	7.50	0.9195	1.0352
Isoform 1 of Bromodomain adjacent to zinc finger domain protein 2A	208.23	6.67	0.9995	1.0353
cDNA FLJ14191 fis, clone NT2RP2006554	72.44	8.66	0.8492	1.0353
Isoform 1 of Syntaxin-binding protein 5-like	131.80	6.95	1.0541	1.0355
Tu translation elongation factor, mitochondrial precursor	49.84	7.61	0.9257	1.0355
Eukaryotic peptide chain release factor subunit 1	49.00	5.71	1.1925	1.0357
Nuclear receptor-binding protein	59.81	5.08	0.8324	1.0360
Isoform 1 of Dynein light chain roadblock-type 1	10.91	7.25	0.9011	1.0360
V_segment translation product	15.25	8.48	0.9607	1.0362
Leucine-rich repeat protein SHOC-2	64.85	8.46	0.8653	1.0362
Coagulation factor X	54.70	5.94	1.1917	1.0362
C18orf34 protein (Fragment)	34.45	7.24	0.9044	1.0362
Isoform 2 of Zinc finger protein 644	138.92	7.81	0.9221	1.0364
15 kDa protein	15.18	11.53	0.8025	1.0366
Isoform NKG2-B of NKG2-A/NKG2-B type II integral membrane protein	24.21	7.65	1.1185	1.0367
Conserved hypothetical protein	20.52	8.95	1.3472	1.0369
Endoplasmin	92.41	4.84	0.8767	1.0373
Spermidine synthase	33.80	5.49	0.9089	1.0375
cDNA FLJ50334	23.30	9.00	1.3075	1.0375
Osteoglycin	30.44	8.34	0.8439	1.0383
Protein O-linked-mannose beta-1,2-N-acetylglucosaminyltransferase 1	75.17	6.83	0.9906	1.0383
Heat shock 70 kDa protein 6	70.98	6.14	0.8859	1.0384
Actin, alpha cardiac muscle 1	41.99	5.39	0.9503	1.0384
cDNA FLJ58803, highly similar to Cytochrome P450 26B1	55.66	9.13	0.7777	1.0384
Ribosomal protein S8	21.87	10.36	0.9401	1.0384
Zinc fingers and homeoboxes protein 1	98.04	6.05	0.9694	1.0385
40S ribosomal protein S23	15.80	10.49	1.0277	1.0386
Isoform 1 of Protocadherin Fat 3	505.21	4.87	1.0121	1.0386
cDNA FLJ31621 fis, clone NT2RI2003205, highly similar to Transportin-2	78.81	5.11	0.8632	1.0388
DnaJ homolog subfamily C member 8	29.82	9.06	0.8134	1.0388
Methionine synthase	140.44	5.58	1.1643	1.0388
Pseudogene candidate	11.22	9.58	0.8659	1.0389

cDNA FLJ59001, highly similar to CpG-binding protein	24.28	7.24	0.9982	1.0390
cDNA FLJ59174, highly similar to Ubiquitin-conjugating enzyme E2 L3	14.11	8.92	0.9790	1.0393
Isoform 1 of Polypyrimidine tract-binding protein 1	57.19	9.17	1.0072	1.0394
Isoform Beta of Signal transducer and activator of transcription 1-alpha/beta	82.99	6.42	0.9180	1.0396
Actin/actin-like family protein	24.56	7.78	0.9535	1.0396
Asparaginyl-tRNA synthetase, cytoplasmic	62.90	6.25	0.8663	1.0399
Cytochrome b5 type B	16.32	4.97	1.0272	1.0399
FYVE finger-containing phosphoinositide kinase	236.96	6.71	0.9992	1.0403
Isoform 3 of Aldehyde dehydrogenase family 8 member A1	28.68	6.87	0.9079	1.0405
cDNA FLJ54475, moderately similar to Mus musculus syncoilin (Sync), mRNA	54.78	4.63	0.9355	1.0407
cDNA FLJ58289, highly similar to Serine hydroxymethyltransferase, mitochondrial	56.34	9.42	0.9512	1.0407
59 kDa protein	59.44	5.78	0.9393	1.0408
cDNA FLJ38640 fis, clone HHDPC2003472, highly similar to CHLORIDE INTRACELLULAR CHANNEL PROTEIN 4	26.68	5.58	1.0866	1.0408
Vacuolar protein sorting-associated protein 33B	70.57	6.71	1.0810	1.0408
10 kDa protein	9.83	9.17	0.9444	1.0411
Ubiquitin carboxyl-terminal hydrolase 24	294.18	6.14	0.9643	1.0411
40S ribosomal protein S6	28.66	10.84	0.9522	1.0413
26S protease regulatory subunit 4	49.15	6.21	0.9636	1.0414
cDNA FLJ52264, highly similar to Ubiquitin-conjugating enzyme E2 E1	18.76	8.75	1.0316	1.0414
Putative uncharacterized protein SFRS5	30.49	11.66	1.0741	1.0415
Importin-7	119.44	4.82	1.2224	1.0416
cDNA FLJ58643, highly similar to Homo sapiens sel-1 suppressor of lin-12-like (SEL1L), mRNA	65.44	9.51	0.9768	1.0417
14 kDa protein	13.57	8.41	0.9668	1.0420
Isoform 2 of UTP--glucose-1-phosphate uridylyltransferase	55.64	7.88	1.2542	1.0420
Isoform 3 of Cell cycle checkpoint protein RAD1	7.57	6.51	0.9410	1.0420
round spermatid basic protein 1	90.02	8.60	0.8038	1.0421
Valyl-tRNA synthetase	140.39	7.59	1.1335	1.0422
Lysyl-tRNA synthetase	68.00	6.35	1.0350	1.0423
Leucine-rich repeat and fibronectin type-III domain-containing protein 6	89.63	7.62	1.0016	1.0424
Thyrotropin-releasing hormone-degrading ectoenzyme	116.93	6.99	1.1522	1.0425
ATP-dependent RNA helicase DDX1	82.38	7.23	0.9406	1.0426
zinc finger protein 292	304.62	7.39	1.1353	1.0428
Leucine-rich repeat-containing protein 8B	92.33	6.86	1.0678	1.0428
Isoform 1 of Lipid phosphate phosphohydrolase 1	32.14	7.97	0.9236	1.0429
Isoform 2 of Glycosyltransferase-like protein LARGE1	81.79	7.74	1.1938	1.0431
Isoform 2 of ADAM 9	72.31	6.73	0.9348	1.0431
Isoform Beta of Heat shock protein 105 kDa	92.06	5.55	0.9500	1.0434
Conserved hypothetical protein	4.42	8.25	1.1507	1.0434
Isoform 4 of Long-chain-fatty-acid--CoA ligase ACSBG2	51.86	8.40	1.0444	1.0434
Cytosolic non-specific dipeptidase	52.84	5.97	0.9675	1.0434
Zinc finger and BTB domain-containing protein 45	53.97	6.92	0.9778	1.0435
Endothelial PAS domain-containing protein 1	96.40	6.28	0.9842	1.0435
Isoform 3 of Uncharacterized protein KIAA1109	479.06	6.67	0.7532	1.0436
Isoform 1 of ATP synthase subunit d, mitochondrial	18.48	5.30	1.0220	1.0437
ADP/ATP translocase 3	32.85	9.74	1.0496	1.0438
ADNP homeobox protein 2	122.75	9.16	1.0584	1.0439
ATP-binding cassette sub-family D member 4	68.55	6.55		1.0439
cDNA FLJ90405 fis, clone NT2RP2006099	26.11	11.75	1.0602	1.0440
cDNA FLJ55894, highly similar to Discs large homolog 7	88.87	8.53	1.0671	1.0441
Isoform 1 of Heat shock protein HSP 90-alpha	84.61	5.02	1.0202	1.0442
Isoform 3 of Zinc finger CCCH-type antiviral protein 1	67.55	8.72	1.0494	1.0442
Isoform 1 of Protein C2orf37	58.74	7.01	0.9726	1.0443
Pleckstrin homology domain-containing family G member 4B	139.35	6.67	0.9887	1.0443
39S ribosomal protein L37, mitochondrial	48.09	8.59	0.9040	1.0443
Fatty acid synthase	273.23	6.43	0.9711	1.0444

Prohibitin-2	33.28	9.83	1.0207	1.0444
Keratin, type II cuticular Hb4	64.85	7.74	1.1484	1.0447
Isoform 2 of Voltage-dependent anion-selective channel protein 2	30.39	7.20	0.8650	1.0447
UPF0526 protein	84.35	6.90	1.4934	1.0448
60S ribosomal protein L17	21.38	10.17	1.0514	1.0451
Isoform 2 of Vacuolar protein sorting-associated protein 13D	488.75	6.55	0.7591	1.0451
Neuronal acetylcholine receptor subunit alpha-5	53.02	6.98	0.9598	1.0452
Isoform 2 of Spatacsin	236.28	5.67	0.9988	1.0454
Charged multivesicular body protein 4b	24.93	4.82	0.9086	1.0457
Zinc finger protein 654	65.80	5.58	0.9652	1.0457
28 kDa protein	28.13	6.54	1.2130	1.0458
Uncharacterized protein LOC150297	27.67	5.14	1.1601	1.0459
Isoform p66Shc of SHC-transforming protein 1	62.78	6.44	0.9037	1.0459
cDNA FLJ59357, highly similar to Probable ATP-dependent RNA helicase DDX5	61.48	8.85	0.9957	1.0460
Interferon-induced 17 kDa protein	17.88	7.44	0.8894	1.0461
Isoform 1 of Tau-tubulin kinase 1	142.65	5.60	0.8559	1.0461
Fascin	54.50	7.24	0.9811	1.0462
Isoform 2 of HORMA domain-containing protein 1	44.39	5.78	1.0557	1.0462
cDNA FLJ61762	15.27	8.82	1.1057	1.0463
cDNA FLJ16663 fis, clone THYMU2005759, highly similar to Guanine nucleotide-binding proteinG(I)/G(S)/G(T) subunit beta 1	36.02	5.99	0.9504	1.0467
hypothetical protein LOC57481	186.90	8.51	1.0283	1.0467
Isoform 4 of Arsenite-resistance protein 2	100.09	6.01	0.9495	1.0467
chaperonin containing TCP1, subunit 3 isoform b	60.42	6.49	0.9562	1.0467
Isoform 2 of Probable phospholipid-transporting ATPase IK	146.66	7.90	1.0305	1.0468
transmembrane protein 2 isoform b	147.35	8.38	1.0218	1.0468
Tubulin beta-2C chain	49.80	4.89	0.8493	1.0469
Coiled-coil domain-containing protein 154	75.97	8.38	1.0456	1.0470
60S ribosomal protein L8	28.01	11.03	1.0396	1.0471
12 kDa protein	12.01	7.81	1.0693	1.0471
FERM and PDZ domain-containing protein 1	173.33	5.25	0.8894	1.0472
kalirin, RhoGEF kinase isoform 1	340.05	6.07	1.3739	1.0472
60S ribosomal protein L23a	17.68	10.45	1.0280	1.0473
Isoform 2 of Eukaryotic translation initiation factor 2-alpha kinase 4	183.53	6.46	1.1908	1.0473
Pericentrin	377.85	5.54	0.8810	1.0474
Deubiquitinating protein VCIP135	134.24	7.20	0.9727	1.0475
Pleckstrin homology domain containing, family A (Phosphoinositide binding specific) member 1	43.64	9.22	0.9962	1.0476
similar to nebulin	47.28	9.01	1.3549	1.0478
38 kDa protein	37.97	10.67	0.8985	1.0480
Isoform 1 of Putative sulfotransferase 1C3	35.87	6.92	0.9763	1.0481
Transportin-1	101.24	4.96	0.9741	1.0482
Coiled-coil domain-containing protein 38	65.27	8.75	0.9569	1.0483
27 kDa protein	27.07	6.79	1.0125	1.0483
Alanyl-tRNA synthetase, cytoplasmic	106.74	5.53	0.9331	1.0484
Atrial natriuretic peptide-converting enzyme	116.49	5.02	0.9129	1.0486
cDNA FLJ56442, highly similar to ATP-citrate synthase	125.06	8.18	0.9931	1.0487
Hepatoma-derived growth factor	26.77	4.73	0.7407	1.0488
Putative uncharacterized protein RNPEP	68.13	6.01	1.0245	1.0489
Isoform 1 of RuvB-like 1	50.20	6.42	1.0520	1.0491
elongation factor Tu GTP binding domain containing 2 isoform b	105.32	5.22	0.9630	1.0492
cDNA FLJ44920 fis, clone BRAMY3011501, highly similar to Heterogeneous nuclear ribonucleoprotein U	83.03	8.79	0.9937	1.0492
12 kDa protein	11.90	9.48	0.9330	1.0494
Isoform KGA of Glutaminase kidney isoform, mitochondrial	73.41	7.77	0.8023	1.0494
14 kDa protein	13.51	7.30	0.8150	1.0496
HECT, UBA and WWE domain containing 1	374.07	5.10	0.9629	1.0496

Bile salt export pump	146.30	6.57	0.8871	1.0496
Lupus brain antigen 1 homolog	336.01	6.76	0.9584	1.0496
CD59 glycoprotein	14.17	6.48	1.1241	1.0498
Calumenin, isoform CRA_c	38.03	4.63	0.9356	1.0499
11 kDa protein	11.45	7.94		1.0499
Isoform 17 of Fibroblast growth factor receptor 2	86.57	6.55	0.9816	1.0499
60S ribosomal protein L5	34.34	9.72	0.9484	1.0501
Transmembrane protein TTMA	63.89	10.08	1.2282	1.0503
Isoform 2 of Leucine zipper protein 1	114.52	8.88	1.0958	1.0503
203 kDa protein	202.78	5.88	0.8586	1.0504
Putative uncharacterized protein ITGAE	104.92	6.15	0.8925	1.0504
Putative RNA-binding protein 3	17.16	8.91	0.8796	1.0505
Isoform 1 of Myosin-7B	221.25	5.96	0.9256	1.0506
Amphoterin-induced protein 3	55.21	7.87	0.8923	1.0506
Guanine nucleotide-binding protein G(I)/G(S)/G(O) subunit gamma-12	8.00	8.97		1.0507
Uncharacterized protein C9orf119	26.72	10.24	1.1733	1.0508
27 kDa protein	27.13	8.76	0.9780	1.0510
Conserved hypothetical protein	23.35	11.43	0.8762	1.0511
60S ribosomal protein L4	47.67	11.06	1.0093	1.0512
Putative uncharacterized protein ENSP00000323645	13.49	9.58	0.9277	1.0513
Isoform 1 of Tripartite motif-containing protein 42	82.72	7.93	0.9846	1.0516
Probable G-protein coupled receptor 33	38.21	9.51	1.0304	1.0516
Motor neuron and pancreas homeobox protein 1	40.73	7.68	0.9841	1.0517
Zinc finger protein 592	137.44	7.84	1.0259	1.0517
similar to ATPase, Ca++ transporting, plasma membrane 2	81.06	6.34	1.2554	1.0519
Conserved hypothetical protein	12.08	9.55	1.1161	1.0519
Isoform 2 of Netrin receptor UNC5B	102.37	5.90	1.0371	1.0522
cDNA FLJ38664 fis, clone HLUNG2002334, highly similar to Homo sapiens KH domain containing, RNA binding, signal transduction associated 2 (KHDRBS2), mRNA	35.32	8.72	1.1893	1.0524
similar to hCG2026352	20.01	9.91	0.8918	1.0525
Putative LRRC3-like protein ENSP00000367157	29.30	6.62	0.9583	1.0526
deleted in liver cancer 1 isoform 1	170.49	6.40	1.0200	1.0527
General transcription factor 3C polypeptide 4	91.92	6.65	1.4511	1.0527
THO complex subunit 4	27.54	11.05	0.8726	1.0528
6-phosphogluconate dehydrogenase, decarboxylating	53.11	7.23	0.8533	1.0532
zinc finger homeodomain 4	393.85	6.37	1.3300	1.0533
Isoform 1 of Histone-lysine N-methyltransferase MLL3	541.03	6.49	0.9105	1.0535
human immunodeficiency virus type I enhancer binding protein 1	296.68	7.84	0.6493	1.0537
47 kDa protein	47.09	7.46	0.9246	1.0537
Isoform Non-brain of Clathrin light chain A	23.65	4.53	1.0063	1.0538
Isoform 3 of PX domain-containing protein kinase-like protein	38.85	9.48	1.0401	1.0538
Eukaryotic translation initiation factor 4E type 1B	27.58	7.17	0.9257	1.0539
cDNA FLJ59767, highly similar to T-cell surface antigen CD2	30.53	9.39	1.1123	1.0540
Isoform 2 of Ataxin-2	105.98	9.67	0.8440	1.0540
Citrate synthase, mitochondrial	51.68	8.32	0.9762	1.0540
Mitotic spindle assembly checkpoint protein MAD1	83.02	5.92	1.1542	1.0541
amyotrophic lateral sclerosis 2 (juvenile) chromosome region, candidate 12 isoform b	49.44	6.42	1.0544	1.0542
isoleucyl-tRNA synthetase	144.41	6.15	1.0855	1.0542
chaperonin containing TCP1, subunit 6A isoform b	53.26	7.27	0.9256	1.0545
Uncharacterized protein FLJ46558	19.92	7.46	1.0809	1.0552
Isoform 3 of FK506-binding protein 15	50.30	6.80	0.9339	1.0552
60S ribosomal protein L27a	16.55	11.00	1.0621	1.0553
Perilipin	55.92	6.42	1.3727	1.0553
Ras GTPase-activating-like protein IQGAP3	184.43	7.49	1.0820	1.0555
Isoform 5 of Sperm antigen with calponin homology and coiled-coil domains 1	78.97	5.34	1.3628	1.0555

Adenylosuccinate synthetase isozyme 2	50.07	6.55	1.1835	1.0558
Isoform 3 of Coiled-coil domain-containing protein 142	72.43	6.95	1.1783	1.0558
cDNA FLJ40076 fis, clone TESTI2000874, highly similar to Superoxide dismutase	19.72	8.03	0.8707	1.0558
HECT domain containing 1	289.20	5.35	1.0541	1.0559
1-aminocyclopropane-1-carboxylate synthase-like protein 2	65.29	6.58	1.1086	1.0561
Isoform 1 of Protocadherin gamma-B6	100.98	5.26	0.8759	1.0561
Isoform 2 of RING finger protein 166	14.40	7.05	0.9537	1.0562
11 kDa protein	10.75	5.06	1.2594	1.0563
Isoform 2 of Clathrin heavy chain 1	187.77	5.69	1.0454	1.0565
cDNA FLJ50661, highly similar to Dihydrolipoyl dehydrogenase, mitochondrial	49.25	7.68	0.8910	1.0566
Golgin subfamily A member 1	88.15	5.27	0.8064	1.0567
6 kDa protein	6.41	8.03	1.2875	1.0568
SCY1-like protein 2	103.64	8.22	0.9976	1.0569
Spectrin, alpha, erythrocytic 1	279.50	5.05	0.9486	1.0572
Isoform 1 of C2 domain-containing protein 2-like	76.13	7.69	0.9563	1.0572
X-prolyl aminopeptidase (Aminopeptidase P) 1, soluble	20.62	6.21	1.2108	1.0574
Heterogeneous nuclear ribonucleoprotein H	49.20	6.30	0.9350	1.0575
17 kDa protein	16.86	9.33	0.9716	1.0580
Heparin cofactor 2	57.03	6.90	1.1030	1.0580
Putative zinc finger protein	134.98	8.00	0.9234	1.0581
15 kDa protein	15.24	4.96	0.8887	1.0583
Isoform 2 of NEDD4-binding protein 2	196.70	5.22	1.1465	1.0583
LETM1 and EF-hand domain-containing protein 1, mitochondrial	83.30	6.70	1.0784	1.0584
Conserved hypothetical protein	9.96	8.91	1.0034	1.0585
Uncharacterized protein C18orf21 (Fragment)	25.22	10.26	1.1119	1.0592
Putative uncharacterized protein	41.52	8.81	1.0190	1.0593
Cathepsin D	44.52	6.54	1.0632	1.0594
Isoform 1 of Serine/threonine-protein kinase DCLK1	81.05	8.66	0.9841	1.0597
Isoform 1 of IQ motif and SEC7 domain-containing protein 2	161.64	8.56	1.0766	1.0598
14 kDa protein	13.89	4.89	1.0264	1.0601
Tyrosine-protein phosphatase non-receptor type 14	135.18	8.31	1.0940	1.0601
Semaphorin-3G	86.65	7.78	1.0170	1.0604
electron transfer flavoprotein, alpha polypeptide isoform b	30.01	8.57	0.9433	1.0605
Homeodomain interacting protein kinase 2	88.59	8.47	0.9117	1.0608
Isoform 3 of Mediator of RNA polymerase II transcription subunit 23	156.09	7.40	1.0002	1.0608
Coatomer subunit gamma	97.66	5.47	1.1396	1.0610
Keratin, type II cytoskeletal 5	62.34	7.74	1.1149	1.0610
Heat shock protein 90Bf	41.43	4.89	0.9107	1.0612
10 kDa protein	10.28	10.08	0.9578	1.0612
similar to hCG2041190	28.83	5.60	1.1549	1.0614
Olfactory receptor 52I2	38.33	8.10	1.4095	1.0616
Isoform 2 of 2-amino-3-carboxymuconate-6-semialdehyde decarboxylase	31.19	6.42	0.9423	1.0616
Probable E3 ubiquitin-protein ligase HERC2	527.14	6.27	1.2518	1.0616
C-1-tetrahydrofolate synthase, cytoplasmic	101.50	7.30	0.9808	1.0619
hypothetical protein LOC729830	119.69	4.92	1.0791	1.0619
cDNA FLJ56447, highly similar to Dynamin-2	80.49	8.13	0.9116	1.0620
Heat shock 70 kDa protein 1	70.01	5.66	0.9356	1.0620
Isoform 3 of Probable E3 ubiquitin-protein ligase HECTD3	23.95	4.92	0.9492	1.0622
Sedoheptulokinase	51.47	6.83	0.9039	1.0623
60S ribosomal protein L7	29.21	10.65	0.8599	1.0623
Isoform 2 of Adenylosuccinate lyase	48.30	7.78	0.9424	1.0624
Isoform 1 of Dynein heavy chain 10, axonemal	514.48	5.88	1.2176	1.0625
Isoform Short of Glucose-6-phosphate 1-dehydrogenase	59.22	6.84	0.9204	1.0627
Isoform 1 of Uncharacterized protein C20orf117	159.66	6.46	0.8033	1.0627
Kelch-like protein 6	69.11	6.21	0.9856	1.0630
Sodium-independent sulfate anion transporter	65.26	7.31	0.9766	1.0631

cDNA FLJ58118, highly similar to Leucine-rich repeat-containing G-protein coupled receptor 5	85.65	6.99	0.9846	1.0632
Poly(rC)-binding protein 1	37.47	7.09	1.0955	1.0632
cDNA FLJ59779, highly similar to Zinc finger protein 398	71.99	6.77	0.9078	1.0633
Ribosomal protein L14 variant	23.77	10.93	1.0052	1.0635
DNA replication licensing factor MCM2	101.83	5.52	1.4923	1.0635
24 kDa protein	23.66	9.80	0.9675	1.0636
cDNA FLJ56638, highly similar to Homo sapiens paternally expressed 10 (PEG10), transcript variant 1, mRNA	45.05	7.93	0.6743	1.0637
Isoform 2 of RING finger protein 180	47.26	9.07	1.0132	1.0640
Coronin-1B	28.93	7.88	0.9716	1.0640
NMDA receptor subunit 3B	97.23	9.10	1.1512	1.0641
26S proteasome non-ATPase regulatory subunit 3	60.94	8.44	1.0188	1.0642
Armadillo repeat-containing X-linked protein 3	42.47	8.37	0.9810	1.0643
cDNA FLJ54408, highly similar to Heat shock 70 kDa protein 1	63.88	5.55	1.0110	1.0643
ATP-binding cassette sub-family E member 1	67.27	8.34	1.0762	1.0645
cDNA FLJ52292, highly similar to Ubiquinol-cytochrome-c reductase complex coreprotein I, mitochondrial	24.09	5.87	0.9545	1.0646
Isoform 1 of THO complex subunit 2	169.47	8.68	1.0571	1.0649
cDNA FLJ58027	47.35	6.84	1.0472	1.0650
calponin 2 isoform b	29.48	6.99	1.0997	1.0650
Isoform 2 of Acyl-CoA synthetase family member 3, mitochondrial	57.78	8.40	1.1799	1.0651
22 kDa protein	21.94	9.67	0.6353	1.0651
60S ribosomal protein L18	21.62	11.72	0.9994	1.0653
7 kDa protein	7.04	10.54	0.8159	1.0654
Isoform 1 of Putative death domain-containing protein FLJ16686	101.49	7.09	1.1296	1.0655
50 kDa protein	49.98	6.67	1.0923	1.0656
Protein ERGIC-53	57.51	6.77	1.0225	1.0657
Isoform 2 of Transcription intermediary factor 1-beta	79.42	5.99	1.3053	1.0657
Isoform 1 of Titin	3813.81	6.34	1.1853	1.0657
HLA class II histocompatibility antigen, DRB3-1 beta chain	29.94	8.56	1.0253	1.0660
Tropomodulin-3	39.57	5.19	1.0695	1.0662
Conserved hypothetical protein	17.54	11.81	0.9298	1.0663
60S ribosomal protein L23	14.86	10.51	0.9984	1.0663
similar to hCG1642996	214.42	5.99	0.8846	1.0663
Niban-like protein 1	82.63	6.15	1.0580	1.0663
Heat shock protein HSP 90-beta	83.21	5.03	0.9400	1.0663
ARFGAP with coiled-coil, ANK repeat and PH domain-containing protein 2	87.97	6.80	1.0609	1.0665
lung type-I cell membrane-associated glycoprotein isoform d	12.16	4.81	0.9162	1.0665
Putative uncharacterized protein ZNF827 (Fragment)	118.90	7.08	1.0575	1.0666
Isoform 3 of PHD finger protein 19	14.38	8.54	1.0385	1.0669
Ephrin-B1	37.98	8.94	0.9686	1.0669
18 kDa protein	17.49	10.98	0.9691	1.0670
18 kDa protein	17.98	4.86	1.0034	1.0671
coiled-coil domain containing 149 isoform 2	59.25	5.91	1.1386	1.0672
AP-4 complex subunit epsilon-1	127.21	5.99	0.9849	1.0672
Large neutral amino acids transporter small subunit 1	54.97	7.72	0.8305	1.0672
cDNA FLJ53015, highly similar to Homo sapiens GTP binding protein 3 (GTPBP3), transcript variant IV, mRNA	15.50	11.37	1.0050	1.0673
20 kDa protein	19.56	7.28	2.5006	1.0674
Isoform 2 of 26S proteasome non-ATPase regulatory subunit 1	102.19	5.27	1.0417	1.0676
9 kDa protein	8.61	7.12	0.9948	1.0679
cDNA PSEC0120 fis, clone PLACE1002379, highly similar to Human alpha-1,3-mannosyl-glycoprotein beta-1, 2-N-acetylglucosaminyltransferase (MGAT) gene	34.95	8.90	1.5280	1.0679
Calicin	66.54	8.18	1.0344	1.0681
Putative uncharacterized protein	12.10	9.79	1.1739	1.0685
similar to hCG2040272	18.29	9.03	0.9231	1.0686
similar to hCG2036631	31.01	5.14	1.0588	1.0688

Similar to Anti-FactorVIII scFv	13.23	8.88	0.9330	1.0691
hypothetical protein	15.59	9.10	0.8995	1.0692
Tetratricopeptide repeat protein 17	129.48	6.58	0.9952	1.0693
Calcium-binding and coiled-coil domain-containing protein 2	52.22	5.02	0.5870	1.0695
F-box only protein 40	79.73	7.11	0.9947	1.0698
9 kDa protein	8.94	5.76	0.8991	1.0700
Isoform 7 of Protein kinase C-binding protein 1	136.95	7.08	0.8100	1.0702
40S ribosomal protein S4, X isoform	29.58	10.15	0.9555	1.0703
Isoform 1 of Dynein heavy chain 6, axonemal	475.68	6.00	0.9279	1.0705
cDNA FLJ16773 fis, clone BRAWH3046240, highly similar to Probable G protein-coupled receptor GPR35	43.28	8.88	0.9512	1.0710
cDNA FLJ55002, highly similar to Alpha-centractin	38.25	6.96	0.9726	1.0712
proteasome beta 5 subunit isoform 2	17.77	7.24	1.0880	1.0714
Glycogen phosphorylase, liver form	97.09	7.17	0.9751	1.0714
Isoform 3 of HD domain-containing protein 2	8.16	11.24	0.9886	1.0715
UPF0553 protein C9orf64	39.00	5.88	0.9840	1.0715
Isoform 2 of Homeobox protein BarH-like 1	24.02	9.64	1.2564	1.0717
cDNA FLJ57743, highly similar to Mannose-P-dolichol utilization defect 1 protein	27.52	8.65	1.0781	1.0719
Isoform 3 of Testis-expressed protein 14	162.57	5.25		1.0720
G-protein coupled receptor	101.42	8.50	0.9969	1.0721
Isoform 2 of NACHT, LRR and PYD domains-containing protein 4	107.28	5.91	0.9276	1.0721
PHD finger-like domain-containing protein 5A	12.40	8.41	0.9560	1.0722
Isoform 2 of Palmitoyltransferase ZDHHC3	34.15	8.18	0.9439	1.0722
19 kDa protein	18.85	9.80	1.1250	1.0725
Isoform 1 of Uncharacterized protein KIAA1529	190.98	6.04	0.9781	1.0726
histone deacetylase 2	65.50	6.99	0.9006	1.0728
mTERF domain-containing protein 3, mitochondrial	44.39	8.97		1.0728
Uncharacterized protein DKFZp566E164	22.82	6.16		1.0728
Serine/threonine-protein kinase PLK4	108.90	8.62	0.7374	1.0729
Ubiquitin specific peptidase 49	67.44	9.64	1.2351	1.0733
Uncharacterized protein C7orf38	66.23	6.21	1.0082	1.0734
cDNA FLJ61344, highly similar to Maternal embryonic leucine zipper kinase	70.09	9.04	1.5613	1.0735
Isoform 1 of Sacsin	520.79	7.05	1.0898	1.0735
Isoform 3 of Formin-binding protein 1	68.84	5.81	1.1310	1.0735
Protein S100-A11	11.73	7.12	1.0039	1.0736
cDNA FLJ59139, highly similar to Vesicle-associated membrane protein-associated protein B/C	13.35	8.12	0.8819	1.0738
Long-chain-fatty-acid--CoA ligase ACSBG1	81.21	6.02	0.7690	1.0739
epsilon subunit of coatomer protein complex isoform c	28.75	5.29	1.0845	1.0739
cDNA FLJ59173	19.07	7.58	1.2442	1.0744
cDNA FLJ57197, highly similar to T-box transcription factor TBX5	47.78	8.82	1.0142	1.0744
Isoform 4 of Coiled-coil domain-containing protein 88B	151.03	5.02	0.8765	1.0745
zinc finger protein 529 isoform a	69.14	8.24	1.0756	1.0746
Isoform 2 of Cysteine-rich with EGF-like domain protein 1	45.91	5.81	0.8828	1.0748
Putative uncharacterized protein PRO2015	10.76	11.59	1.0188	1.0749
cDNA FLJ54917, highly similar to Lymphocyte-specific adapter protein Lnk	41.39	6.96	1.2514	1.0750
Isoform 1 of LIM and SH3 domain protein 1	29.70	7.05	0.8937	1.0750
ArfGAP with SH3 domain, ankyrin repeat and PH domain 3 isoform b	97.79	6.40	0.8434	1.0752
23 kDa protein	23.09	8.56	0.9619	1.0753
37 kDa protein	37.41	8.19	0.8720	1.0754
Ras-related protein Rab-6C	28.34	7.71	0.8394	1.0754
anoctamin 5 isoform b	106.99	6.80	0.9561	1.0756
cDNA FLJ11861 fis, clone HEMBA1006885, highly similar to Proline synthetase co-transcribed bacterial homolog protein	25.92	6.43	1.0280	1.0759
Isoform 2 of RNA-binding protein 8A	19.75	5.92	0.9371	1.0759
T-complex protein 1 subunit alpha	60.31	6.11	0.9600	1.0765
Putative uncharacterized protein XRCC5	81.32	5.68	0.9606	1.0766

Importin subunit beta-1	97.11	4.78	1.0338	1.0766
Isoform 1 of Engulfment and cell motility protein 1	83.78	6.28	0.8847	1.0768
Superoxide dismutase [Cu-Zn]	15.93	6.13	1.0132	1.0768
8 kDa protein	7.97	10.86	1.4537	1.0770
Isoform 2 of Retinoblastoma-like protein 1	114.61	7.50	1.0460	1.0770
hypothetical protein	22.02	12.10	1.0160	1.0771
SLIT and NTRK-like protein 4	94.27	7.80	1.1671	1.0772
Isoform 3 of Probable Xaa-Pro aminopeptidase 3	31.51	9.57	1.1391	1.0773
Pre-mRNA branch site protein p14	14.58	9.38	0.9180	1.0779
SEC16 homolog A	191.95	5.94	0.8397	1.0779
Putative uncharacterized protein MDH1	23.02	7.40	0.9709	1.0781
Vigilin	141.37	6.87	1.0080	1.0783
Isoform 1 of Oxysterol-binding protein 1	89.36	7.30	0.8164	1.0788
Isoform 1 of NUAK family SNF1-like kinase 1	74.26	8.82	1.0387	1.0789
Isoform 1 of ERC protein 2	110.49	6.99	0.8793	1.0790
Isoform 1 of Centrosomal protein of 290 kDa	290.37	5.95	1.0233	1.0791
Apolipoprotein B-100	515.24	7.06	0.9583	1.0792
cDNA FLJ55366, highly similar to Mitogen-activated protein kinase kinase kinase kinase 3	50.55	8.32	0.9085	1.0797
similar to LOC339742 protein	22.13	11.18	0.9638	1.0797
Beta-2-microglobulin	13.71	6.52	1.1092	1.0802
Isoform 2 of Sperm flagellar protein 2	209.18	5.60	0.9047	1.0803
transmembrane channel-like 4 isoform 2	78.61	8.98	0.8467	1.0803
similar to heterogeneous nuclear ribonucleoprotein A1	32.38	9.01	0.9403	1.0804
similar to QM protein isoform 1	24.61	10.08	0.9490	1.0811
Isoform 4 of Plectin-1	515.88	5.80	1.0874	1.0811
Nuclease sensitive element binding protein-1	34.65	7.53	0.9757	1.0811
Isoform 3 of Fanconi anemia group D2 protein	140.65	6.09	1.0257	1.0816
cDNA FLJ51212, moderately similar to Brain acid soluble protein 1	17.65	4.77	1.0779	1.0816
cDNA FLJ53443, highly similar to Leiomodin-1	61.50	9.16	0.9213	1.0816
Coiled-coil domain-containing protein 60	63.05	9.64	0.9706	1.0817
Isoform Long of Antigen KI-67	358.47	9.45	0.9585	1.0821
Isoform 4 of Activating molecule in BECN1-regulated autophagy protein 1	132.76	6.99	1.0481	1.0822
LOC401367	20.40	11.94	1.0948	1.0828
14 kDa protein	13.89	9.85	0.9521	1.0829
30 kDa protein	30.11	10.20	1.0205	1.0831
cortactin isoform b	57.43	5.33	0.8068	1.0834
Ankyrin repeat and SOCS box protein 17	34.26	8.79	0.7824	1.0835
Gastrotropin	14.36	6.80	1.0090	1.0835
Aldose reductase	35.83	6.98	0.7452	1.0835
deoxyuridine triphosphatase isoform 3	15.39	6.57	0.8096	1.0836
60S ribosomal protein L36	12.25	11.59	0.9285	1.0843
Coronin-1C_i3 protein	58.91	7.75	0.8939	1.0844
cDNA FLJ55750, highly similar to Eukaryotic translation initiation factor 3 subunit 8	84.91	6.81	0.9602	1.0844
epiplakin 1	555.28	5.60	1.3709	1.0845
Isoform 3 of Calcium-dependent secretion activator 2	143.41	6.19	1.1864	1.0847
Tektin-4	50.62	6.44	1.0836	1.0847
Isoform 1 of MLX-interacting protein	101.12	8.35	1.0258	1.0849
similar to hCG1774990	30.79	11.33	1.0936	1.0852
hepatitis A virus cellular receptor 1	39.22	6.92	1.1313	1.0852
Zinc finger protein 233	72.60	8.47	0.9846	1.0853
Collagen alpha-1(XIX) chain	115.15	8.32	0.5065	1.0853
Isoform Long of Trifunctional purine biosynthetic protein adenosine-3	107.70	6.70	1.0557	1.0854
Transmembrane protein 69	27.53	9.96		1.0854
Isoform 1 of Spliceosome RNA helicase BAT1	48.96	5.67	1.0139	1.0855
Alpha-fetoprotein	68.63	5.68	0.8934	1.0858

Laminin subunit beta-3	129.49	7.21	0.9036	1.0858
Conserved hypothetical protein	15.43	10.81	1.2981	1.0859
18 kDa protein	17.75	8.35	0.7937	1.0860
C10orf54 protein (Fragment)	33.60	8.78	0.9975	1.0860
Isoform 2 of Phosphatase and actin regulator 1	54.57	6.73	1.2025	1.0862
Eukaryotic initiation factor 4A-III	46.84	6.73	1.0836	1.0864
17 kDa protein	17.07	5.55	0.9344	1.0865
Isoform 3 of NADPH oxidase activator 1	45.22	5.44	1.1234	1.0865
Carboxypeptidase A1	47.11	5.76	1.0391	1.0866
Trinucleotide repeat containing 6B	81.36	8.94	0.9064	1.0866
Melanocortin-2 receptor accessory protein 2	23.53	5.01	0.9626	1.0869
cDNA FLJ52127, highly similar to Multisynthetase complex auxiliary component p43	29.73	9.07	1.0509	1.0869
Isoform 3 of Protein transport protein Sec61 subunit alpha isoform 1	38.82	7.33	1.3345	1.0870
Splicing factor, arginine/serine-rich 11	48.47	10.54	1.0139	1.0874
Putative uncharacterized protein HNRNPA2B1	34.18	9.00	0.9980	1.0875
protein kinase C, iota	68.22	5.85	0.9286	1.0876
cDNA FLJ35580 fis, clone SPLEN2006389, highly similar to fibromodulin	32.10	9.19	0.8962	1.0876
calpain 3 isoform h	11.74	5.39	0.9164	1.0877
Conserved hypothetical protein	9.15	11.65	1.0648	1.0879
65 kDa protein	65.05	9.69	1.0383	1.0884
Isoform 1 of Coiled-coil domain-containing protein 37	71.07	7.11	0.9766	1.0886
R3H domain-containing protein C19orf22	30.33	8.56	1.2594	1.0887
Interferon-gamma receptor alpha chain	54.37	4.91	0.9796	1.0889
Similar to Vegetative cell wall protein gp1 precursor	21.04	9.61	1.1560	1.0891
Isoform 2 of NUAK family SNF1-like kinase 1	23.81	9.82	1.0315	1.0892
similar to metallopanstimulin	9.44	9.44	0.9248	1.0893
cDNA FLJ56531, highly similar to UV excision repair protein RAD23 homolog B	42.28	5.43	0.9002	1.0894
Isoform 2 of Uncharacterized protein KIAA0895	58.27	9.64	1.0036	1.0897
Isoform 2 of Transmembrane protein 41B	21.65	9.04	0.9924	1.0901
Protein	27.82	6.23	1.0594	1.0902
poly(rC) binding protein 2 isoform e	38.13	6.79	1.1119	1.0905
ADP/ATP translocase 2	32.87	9.74	1.0067	1.0906
Isoform 1 of Prickle-like protein 4	37.53	5.67	0.9021	1.0906
Isoform 1 of Long-chain fatty acid transport protein 3	87.27	8.40	0.8318	1.0908
cDNA FLJ54046	46.82	8.25	1.1455	1.0909
LOC649305 protein (Fragment)	19.89	11.02	1.2629	1.0913
Histone-lysine N-methyltransferase SETD1B	208.60	4.96	0.7321	1.0916
Frizzled-9	64.42	8.25	0.9148	1.0917
Isoform 2 of Poly [ADP-ribose] polymerase 2	64.76	8.68	1.0932	1.0920
Protein of unknown function DUF1725 domain containing protein	23.88	9.52	1.1763	1.0921
Histone H1.4	21.85	11.03	0.8713	1.0923
meckelin isoform 2	103.52	6.70	0.8464	1.0927
Eukaryotic translation initiation factor 3 subunit E	52.19	6.04	1.0932	1.0930
Fibrillin-3	300.16	5.07	0.7205	1.0930
Isoform 2 of Ubiquinone biosynthesis methyltransferase COQ5, mitochondrial	27.90	9.14	1.0923	1.0931
Coatomer subunit beta'	102.42	5.27	1.1545	1.0932
cDNA FLJ55812, highly similar to Mus musculus Rho GTPase activating protein 27 (Arhgap27), transcript variant 1, mRNA	100.99	6.25	1.1067	1.0935
cDNA FLJ46488 fis, clone THYMU3026869	19.99	10.86	1.1415	1.0938
Isoform 1 of Rootletin	228.39	5.50	0.8286	1.0938
Hypothetical short protein	7.48	9.00	1.1523	1.0942
11 kDa protein	11.12	11.81	1.0547	1.0943
26S proteasome non-ATPase regulatory subunit 2	100.14	5.20	1.0107	1.0946
Isoform 3 of Cytosolic 5'-nucleotidase 1B	59.12	8.79	1.0612	1.0947
Protein capicua homolog	163.72	8.56	1.2496	1.0947
31 kDa protein	30.83	9.55	0.9339	1.0951

cDNA FLJ36188 fis, clone TESTI2027179, highly similar to Transmembrane 9 superfamily protein member 2	65.39	7.28	1.0674	1.0954
Isoform 3 of Multiple PDZ domain protein	208.28	5.11	0.9209	1.0958
Four and a half LIM domains protein 2	32.17	7.55	1.0704	1.0959
Isoform 1 of Acyl-coenzyme A thioesterase 9, mitochondrial	49.87	8.60	0.9366	1.0961
Uncharacterized protein KIAA1462	148.26	7.09	0.9622	1.0961
Isoform 3 of Heterogeneous nuclear ribonucleoprotein A/B	30.57	7.91	0.9499	1.0962
Isoform 1 of Synaptonemal complex protein 2-like	93.54	5.80	1.1147	1.0962
Isoform 1 of Tropomyosin alpha-4 chain	28.50	4.69	0.6691	1.0966
Isoform 1 of Ankyrin repeat domain-containing protein 26	196.20	5.72	0.9319	1.0966
Putative uncharacterized protein ENSP00000342566	88.46	6.38	0.7900	1.0970
similar to hCG1805148	43.49	4.72	1.2850	1.0973
Isoform Short of Atrial natriuretic peptide receptor B	111.14	6.77	1.1346	1.0974
Isoform 1 of Core histone macro-H2A.1	39.16	9.83	1.0526	1.0978
Bifunctional purine biosynthesis protein PURH	64.58	6.71	1.1128	1.0980
Isoform 1 of Signal-induced proliferation-associated 1-like protein 2	190.32	6.77	0.9831	1.0982
similar to Predicted gene, OTTMUSG00000017677	217.61	10.90	0.9232	1.0988
Isoform 2 of Endosialin	46.21	5.76	0.9110	1.0989
Teneurin-4	307.76	6.55	0.9849	1.0992
RNA binding motif protein 44	118.04	5.72	0.7912	1.0992
Cathepsin O	35.93	7.42	0.7094	1.0993
Putative uncharacterized protein CRYZ	25.97	8.88	1.0022	1.0993
cDNA FLJ53010, highly similar to Soluble calcium-activated nucleotidase 1	38.92	5.33	0.9128	1.0994
12 kDa protein	11.55	9.04	0.7062	1.0994
FP2860	10.95	12.25	1.1050	1.0994
similar to apolipoprotein B editing enzyme catalytic polypeptide-like 3C	15.21	6.77	0.8150	1.0996
Isoform 2 of Spectrin beta chain, brain 2	268.11	6.11	0.7441	1.0996
Tripartite motif-containing protein 43-like protein 2	52.28	7.96	1.1182	1.0997
Ewing's tumor-associated antigen 1	103.38	7.62	1.3972	1.0998
ATP-dependent DNA helicase Q4	132.99	8.09	0.9837	1.1000
Putative glycosyltransferase ALG1-like	21.12	5.00	1.1227	1.1002
Corticosteroid-binding globulin	45.11	6.04	0.8133	1.1007
cDNA FLJ30173 fis, clone BRACE2000969, highly similar to 6-phosphofructokinase, liver type	83.07	7.21	1.0591	1.1008
RuvB-like 2	51.12	5.64	1.1367	1.1009
Zinc finger CCHC domain-containing protein 2	125.86	7.01	0.8368	1.1009
Proteasome activator complex subunit 4 (Proteasome activator PA200). Isoform 2	198.23	6.86	1.6478	1.1011
Von Hippel-Lindau binding protein 1	26.52	9.01	1.0964	1.1012
fMet-Leu-Phe receptor	38.38	9.16	1.1258	1.1012
Conserved hypothetical protein	11.94	10.56	0.9605	1.1020
Gamma-tubulin complex component 2	102.47	6.84	0.8758	1.1022
Surfactant protein D	20.99	9.33	1.0338	1.1022
60S ribosomal protein L10a	24.82	9.94	1.0183	1.1027
Talin-1	269.60	6.07	1.0723	1.1028
cDNA FLJ50935, highly similar to Integral membrane protein 2A	24.98	5.36	1.0545	1.1028
Putative uncharacterized protein	96.08	8.62	1.1805	1.1029
Threonyl-tRNA synthetase, cytoplasmic	83.38	6.67	0.9520	1.1035
Matrilin-3 alternative transcript	48.29	6.76	1.2253	1.1037
Lebercilin	80.51	7.68	0.8497	1.1038
Isoform 6 of Polypyrimidine tract-binding protein 2	38.31	6.57	0.9411	1.1039
Isoform Delta of Apoptosis regulator BAX	15.76	6.70	0.9261	1.1039
cDNA FLJ53910, highly similar to Keratin, type II cytoskeletal 6A	57.80	8.00	0.9890	1.1042
Protein	40.83	7.53	1.4930	1.1043
cDNA FLJ53718, highly similar to Synaptotagmin-15	30.06	8.95	1.1570	1.1043
Isoform 2 of Shugoshin-like 2	144.09	7.68	0.9494	1.1045
Glutamate receptor, ionotrophic, AMPA 3	18.09	8.87	1.1311	1.1047

Endoplasmic reticulum metallopeptidase 1	100.17	7.52	0.9363	1.1049
Isoform 1 of Spermatogenesis-associated serine-rich protein 1	33.68	7.46	1.1017	1.1050
Nuclear transport factor 2	14.47	5.38	1.0920	1.1054
Isoform 5 of CCR4-NOT transcription complex subunit 4	48.45	7.55	1.0121	1.1058
Imunoglobulin heavy chain (Fragment)	10.52	7.94	0.8992	1.1068
53 kDa protein	52.49	7.75	1.2946	1.1070
similar to nucleophosmin	20.37	4.51	1.0571	1.1071
Putative uncharacterized protein ARHGEF11 (Fragment)	172.00	5.50	1.0571	1.1073
Protein	18.47	8.73	1.1242	1.1084
Isoform 1 of Arf-GAP, GTPase, ANK repeat and PH domain-containing protein 2	124.60	9.89	0.8391	1.1084
similar to hCG2021878	17.92	8.92	1.0127	1.1086
Ubiquitin-fold modifier 1	9.11	9.31	1.0879	1.1087
Protein FAN	104.31	6.23	1.5115	1.1089
Putative uncharacterized protein FUCA1	51.24	6.84	1.0267	1.1093
V-type proton ATPase catalytic subunit A	68.26	5.52	0.8186	1.1093
Similar to Alpha (1, 2) fucosyltransferase	10.13	11.25	0.9806	1.1103
Isoform 2 of Laminin subunit alpha-4	201.65	6.33	0.9821	1.1104
Isoform 6 of Calcium/calmodulin-dependent protein kinase kinase 2	54.08	7.20	1.1500	1.1104
Putative male-specific lethal-3 protein-like 2	51.04	8.37	1.2250	1.1105
Nuclear receptor-interacting protein 1	126.86	8.18	1.2260	1.1111
cDNA FLJ52153, highly similar to Transmembrane emp24 domain-containing protein 2	19.25	5.86	0.9287	1.1114
Isoform 2 of SWI/SNF-related matrix-associated actin-dependent regulator of chromatin subfamily E member 1	41.75	6.01	0.7524	1.1114
Kinesin-1 heavy chain	109.62	6.51	1.0719	1.1117
19 kDa protein	19.05	10.43	0.9297	1.1117
cDNA FLJ59713, highly similar to Homo sapiens SHQ1 homolog (SHQ1), mRNA	61.51	4.82	1.0787	1.1119
Putative histone H2B type 2-D	18.01	10.58	1.1527	1.1122
Mitochondrial import receptor subunit TOM22 homolog	15.51	4.34	0.9648	1.1126
Isoform 2 of Splicing factor, arginine/serine-rich 2B	31.41	11.68	0.9443	1.1126
Isoform 1 of Ectonucleotide pyrophosphatase/phosphodiesterase family member 2	98.94	7.37	1.0521	1.1135
13 kDa protein	13.22	7.11	0.9485	1.1137
PEX14 protein (Fragment)	2.09	9.99	0.6662	1.1137
Isoform 2 of Uncharacterized protein C17orf68	114.04	7.28	0.9538	1.1139
sodium channel associated protein 2 isoform b	109.60	6.30	1.2151	1.1140
RUN and FYVE domain-containing 2 isoform a	74.03	6.00	0.8618	1.1141
Estradiol 17-beta-dehydrogenase 12	34.30	9.32	1.0385	1.1143
Isoform 2 of Pre-mRNA-processing factor 40 homolog B	97.79	6.92	1.0484	1.1150
Putative uncharacterized protein CHIT1	28.11	7.59	1.0233	1.1152
Retinoblastoma binding protein 7	46.36	5.07		1.1155
Isoform 1 of Inactive N-acetylated-alpha-linked acidic dipeptidase-like protein 2	88.65	6.23	0.9977	1.1160
87 kDa protein	86.84	9.54	1.2491	1.1166
24 kDa protein	23.53	10.35	0.8529	1.1168
Leucine-rich repeat-containing protein 37A2	188.28	5.50	1.0688	1.1172
Putative uncharacterized protein	41.85	8.69	0.9849	1.1173
18 kDa protein	18.48	7.36	0.9060	1.1173
tripartite motif-containing 66	137.57	6.70	0.9774	1.1177
Dynein heavy chain 5, axonemal	528.68	6.10	1.0410	1.1180
Isoform 2 of Mucosal addressin cell adhesion molecule 1	28.69	5.80	0.9735	1.1181
Isoform 1 of Glutamine-rich protein 2	180.71	6.73	0.9767	1.1181
Putative uncharacterized protein DKFZp564C0482	27.09	5.53	0.8756	1.1182
Trifunctional enzyme subunit alpha, mitochondrial	82.95	9.04	1.0375	1.1182
Isoform 1 of Spermine synthase	41.24	5.02	1.0837	1.1188
Interferon gamma	19.34	9.47	0.8902	1.1195
Leucine-rich repeat and guanylate kinase domain-containing protein	93.56	6.43	0.9417	1.1204
Isoform 3 of Probable G-protein coupled receptor 124	19.52	10.24	1.0040	1.1207

5 kDa protein	5.27	8.19	1.6618	1.1210
PDZ and LIM domain protein 1	36.05	7.02	1.0054	1.1212
Isoform 3 of Neutral alpha-glucosidase AB	96.16	5.72	0.9935	1.1215
80 kDa MCM3-associated protein	218.27	6.39	1.4085	1.1219
Fatty acid-binding protein, epidermal	15.15	7.01	0.9845	1.1222
Mitochondrial import receptor subunit TOM34	34.54	8.98	1.4060	1.1224
Hypoxia up-regulated protein 1	111.27	5.22	1.1059	1.1226
similar to mCG7602	6.62	9.69	0.7865	1.1227
N-acetylglucosamine-6-sulfatase	62.04	8.31	0.9798	1.1227
60S ribosomal protein L19	23.45	11.47	0.8339	1.1227
TRIP12 protein	191.95	6.49	0.9423	1.1231
Potassium channel, subfamily K, member 1	28.68	6.93	0.9864	1.1233
similar to hCG2044193	109.73	8.79	0.9558	1.1236
S100 calcium binding protein A2	10.98	4.78	1.1741	1.1236
zinc finger, CCHC domain containing 11 isoform c	184.59	7.97	0.9304	1.1245
similar to template acyivating factor-I alpha	34.31	4.58	1.0659	1.1249
Putative DYT3 protein (Fragment)	11.37	11.47	0.9094	1.1261
60S acidic ribosomal protein P0	34.25	5.97	0.9979	1.1264
Protein	8.70	9.80	1.0219	1.1265
Stabilin-2	276.81	6.40	0.9964	1.1266
Full-length cDNA clone CS0DI075YC18 of Placenta of Homo sapiens (Fragment)	17.51	11.80	1.0466	1.1270
Isoform 6 of Ataxin-2-like protein	102.83	8.63	0.7815	1.1274
Inactive serine protease 35	47.07	9.69	1.1700	1.1276
Isoform 2 of Mitochondrial import receptor subunit TOM40 homolog	34.42	7.24	1.1451	1.1280
Conserved hypothetical protein	9.40	7.58	1.2574	1.1285
HERV-K_5q13.3 provirus ancestral Pol protein	54.74	9.22	1.0387	1.1290
Isoform Alpha of Nucleolar phosphoprotein p130	73.56	9.47	0.9308	1.1290
156 kDa protein	155.54	8.10	0.7959	1.1293
Isoform 2 of Myosin-VIIa	250.33	8.65	1.0794	1.1296
Isoform 1 of Zinc finger CCCH-type antiviral protein 1-like	32.92	8.13	0.9277	1.1300
SLC30A10 protein	6.51	8.94	0.9101	1.1301
14 kDa protein	13.92	4.70	1.0043	1.1301
Bifunctional aminoacyl-tRNA synthetase	170.54	7.33	1.0346	1.1303
Serine/threonine-protein kinase 10	112.07	6.95	1.3891	1.1305
laminin alpha 3 subunit isoform 4	183.94	8.15	0.8456	1.1305
Isoform 2 of Guanine nucleotide-binding protein-like 3	60.51	8.72	0.8898	1.1308
Isoform 2 of Uncharacterized protein KIAA1586	84.50	8.87	1.1317	1.1308
Isoform 1 of Nuclear ubiquitous casein and cyclin-dependent kinases substrate	27.28	5.08	0.6052	1.1310
Coiled-coil domain containing 44, isoform CRA_a	19.62	10.05	0.9595	1.1311
Protein BTG2	17.40	8.10	1.2024	1.1312
Isoform Long of Non-syndromic hearing impairment protein 5	54.52	5.17	0.9339	1.1312
Isoform 3 of Disks large-associated protein 2	108.84	7.50	0.9881	1.1313
septin 9 isoform a	65.36	8.97	1.0526	1.1316
Scaffold attachment factor B2	107.41	6.16	0.9312	1.1320
cDNA FLJ57962, highly similar to Homo sapiens eukaryotic translation initiation factor (eIF) 2A (eIF2A), mRNA	57.96	9.09	0.9716	1.1337
Proteasome (Prosome, macropain) inhibitor subunit 1	20.58	6.27	1.0247	1.1338
UPF0454 protein C12orf49	23.58	8.10	0.9107	1.1355
cDNA FLJ16102 fis, clone TESTI2015335, highly similar to Serine/threonine-protein kinase 31	95.74	5.15	1.0057	1.1357
18 kDa protein	17.91	8.76	0.9382	1.1357
Ku86 autoantigen related protein 1	18.63	12.32	0.7842	1.1367
Isoform Beta of Tripartite motif-containing protein 31	30.81	7.40	0.9144	1.1368
Isoform 2 of Uncharacterized protein C4orf21	93.58	5.19	1.0103	1.1370
Ribosomal protein S26	8.32	10.73	1.0163	1.1370
cDNA FLJ58832, highly similar to Heterogeneous nuclear ribonucleoprotein A3	34.17	8.10	0.9411	1.1370
Isoform 2 of Serine/threonine-protein phosphatase 4 regulatory subunit 1	105.13	4.81	0.9379	1.1371

Protein	15.49	10.62	1.0590	1.1372
Seizure related 6 homolog (Mouse)-like	79.59	4.65	1.1072	1.1374
Isoform 1 of IQ motif and SEC7 domain-containing protein 3	127.54	6.51	1.0350	1.1376
hypothetical protein	15.00	9.92	0.8203	1.1377
Polycystic kidney disease 2 related protein (Fragment)	76.09	5.59	1.2047	1.1380
cDNA FLJ33480 fis, clone BRAMY2002799, highly similar to Homo sapiens HECT, C2 and WW domain containing E3 ubiquitin protein ligase 1 (HECW1), mRNA	38.84	9.58	1.1154	1.1381
40 kDa protein	39.97	4.70	1.3010	1.1385
cDNA FLJ61494, highly similar to Kinectin	152.89	5.72	0.9580	1.1390
Junction plakoglobin	81.69	6.14	0.8134	1.1391
cDNA FLJ60315	37.51	8.07	0.9630	1.1391
Isoform 3 of Nucleolar protein 8	117.43	6.92	1.5797	1.1392
Putative uncharacterized protein ENSP00000374901	17.48	7.77	1.1494	1.1397
Zinc finger and SCAN domain-containing protein 22	82.29	7.71	1.1246	1.1397
Isoform 2 of PHD finger protein 21A	70.17	9.51	1.1050	1.1398
Isoform 4 of Dynamin-1-like protein	78.05	6.81	1.2609	1.1398
Isoform 2 of Mitochondrial inner membrane protein	82.57	6.57	1.0803	1.1398
Isoform 2 of Prickle-like protein 4	37.52	6.89	0.6892	1.1398
Histone H1.5	22.57	10.92	0.9286	1.1401
cDNA FLJ55515, highly similar to Breast cancer anti-estrogen resistanceprotein 1	95.41	5.68	1.3175	1.1405
ATP-dependent RNA helicase DDX18	75.36	9.50	1.1886	1.1407
similar to putative DUX4 protein	42.12	8.06	1.0752	1.1410
28 kDa protein	28.12	10.04	1.0372	1.1415
Tyrosyl-tRNA synthetase, cytoplasmic	59.11	7.05	0.7880	1.1417
Tissue factor	33.05	7.03	0.7010	1.1418
Nuclear factor I/B	18.34	9.01	0.8919	1.1418
cDNA FLJ55918, highly similar to Echinoderm microtubule-associated protein-like 2	87.18	7.30	0.9059	1.1419
Guanylate-binding protein 4	73.12	6.02	1.1618	1.1419
Isoform 5 of FAD synthetase	36.84	5.35	0.9641	1.1420
Activator of 90 kDa heat shock protein ATPase homolog 1	38.25	5.53	1.1993	1.1421
Isoform 6 of Adenylate kinase 2, mitochondrial	21.75	7.97	1.2950	1.1423
Isoform 1 of Protein FAM57A	25.86	9.52	0.9149	1.1424
SUMO-activating enzyme subunit 2	71.18	5.29	0.9326	1.1425
EF-hand calcium binding domain 8	145.77	8.90	1.1125	1.1427
Sperm-associated antigen 17	251.58	6.24	1.2239	1.1431
cDNA FLJ56566, highly similar to Small glutamine-rich tetratricopeptiderepeat-containing protein A	31.41	5.36	0.9461	1.1436
Hypothetical MGC50722	103.02	9.83	0.9924	1.1439
Transferrin receptor protein 1	84.82	6.61	1.0539	1.1446
Putative uncharacterized protein RAGE	3.31	5.62	0.8855	1.1451
Isoform 1 of Ankyrin repeat domain-containing protein 53	59.49	9.52	0.8716	1.1454
Isoform 1 of Macoilin	76.13	9.07	0.4898	1.1458
LOC100049716 protein (Fragment)	30.61	11.36	0.9435	1.1462
Isoform 2 of Acyl-coenzyme A thioesterase 2, mitochondrial	32.96	7.87	1.2154	1.1462
AFG3-like protein 2	88.53	8.66	0.9856	1.1462
Isoform 1 of Ribose-phosphate pyrophosphokinase 2	34.75	6.61		1.1463
ATP-dependent DNA helicase 2 subunit 1	69.80	6.64	0.9198	1.1466
Zinc finger CCCH domain-containing protein 4	140.17	6.27	1.0011	1.1470
LAG1 longevity assurance homolog 4	46.37	8.95	1.0490	1.1472
Prostaglandin G/H synthase 2	68.95	7.39	1.1764	1.1483
Isoform 2 of Zinc finger and BTB domain-containing protein 41	72.90	7.85	0.9679	1.1485
ATP-binding cassette, subfamily B, member 4 isoform C	135.17	8.38	1.5181	1.1493
Insulin-like growth factor 2 mRNA-binding protein 1	63.42	9.20	0.9117	1.1493
Isoform 1 of Uncharacterized protein KIAA2030	146.00	9.19	1.0093	1.1495
Isoform 2 of Fanconi anemia group I protein	142.35	6.81	1.1763	1.1498
Selectin P	90.63	6.60	1.0091	1.1501

mesenchyme homeobox 1 isoform 3	15.62	9.23	1.1165	1.1505
similar to hCG2014367	65.72	7.44	1.1023	1.1509
GMP synthase [glutamine-hydrolyzing]	76.67	6.87	1.0832	1.1513
Isoform 1 of Uncharacterized protein C1orf141	46.11	9.69	1.3112	1.1514
Isoform B2 of Smoothelin	99.32	8.87	0.9844	1.1521
Fibrocystin-L	465.90	6.14	1.0438	1.1527
Isoform 1 of Uncharacterized protein KIAA0562	104.38	7.62	1.0097	1.1530
v-crk sarcoma virus CT10 oncogene homolog isoform b	22.89	5.48	1.0414	1.1531
5 kDa protein	5.29	9.32	0.9100	1.1534
SH3 domain binding glutamic acid-rich protein like 3	9.37	9.36	1.0013	1.1537
18 kDa protein	18.00	5.86	1.0524	1.1538
Proepiregulin	19.03	7.53	1.2514	1.1547
cDNA FLJ53309, highly similar to Methionyl-tRNA synthetase	71.76	7.14	1.0206	1.1548
Ankyrin repeat domain-containing protein 35	109.90	6.02	1.2039	1.1551
Zinc finger and BTB domain-containing protein 3	61.79	5.62	1.4078	1.1557
Bombesin receptor subtype-3	44.38	8.72	2.1987	1.1559
Isoform 2 of Endothelial differentiation-related factor 1	15.47	9.77	1.0236	1.1560
RNA recognition motif, RNP-1 domain containing protein	18.75	5.03	1.0072	1.1574
cDNA FLJ59565, highly similar to Beta-catenin	80.78	6.09		1.1580
Chromatin assembly factor 1 subunit B	61.45	7.50	0.8572	1.1590
Uncharacterized protein C22orf27	18.19	10.13	1.0442	1.1592
Beta-1,3-galactosyl-O-glycosyl-glycoprotein beta-1,6-N-acetylglucosaminyltransferase 4	53.02	8.25	0.9293	1.1596
Histone H1.3	22.34	11.02	0.9930	1.1600
similar to hCG2025867	14.65	7.40	1.2778	1.1600
16 kDa protein	15.98	9.95	0.9944	1.1606
PRO0433	11.02	9.73	0.9896	1.1610
Isoform 2 of Kinesin-like protein KIF27	152.22	6.83	0.7461	1.1613
Isoform 2 of Ubiquitin-associated protein 2-like	104.91	7.11	0.9711	1.1613
Isoform 1 of Chromodomain-helicase-DNA-binding protein 4	217.85	5.86	0.9361	1.1622
Isoform 2 of Protein TANC1	191.28	8.62	1.1099	1.1622
27 kDa protein	26.53	6.25	0.0000	1.1626
PERQ amino acid-rich with GYF domain-containing protein 1	114.53	5.39	0.9925	1.1627
Isoform 1 of NFX1-type zinc finger-containing protein 1	220.08	7.30	1.4030	1.1629
cDNA FLJ54914, highly similar to Homo sapiens MTERF domain containing 2 (MTERFD2), mRNA	28.65	8.68	0.9866	1.1633
49 kDa protein	49.26	8.19	0.7787	1.1636
Histone H2A.x	15.14	10.74	1.0890	1.1654
cDNA FLJ16039 fis, clone ADRGL2001554, weakly similar to SERINE/THREONINE-PROTEIN KINASE PLK	79.91	7.83	1.3012	1.1654
cDNA FLJ60917	44.95	7.20	1.1170	1.1657
LYR motif-containing protein 4	10.75	10.73	0.9912	1.1668
Isoform 2 of Heterogeneous nuclear ribonucleoprotein M	73.57	8.82	1.0108	1.1674
Isoform 2 of Protein Dok-7	37.14	6.30	0.4394	1.1683
Heterogeneous nuclear ribonucleoprotein A0	30.82	9.29	1.1121	1.1684
Keratin, type I cuticular Ha3-I	45.91	4.82	1.1244	1.1684
Isoform 3 of Zinc finger protein 407	183.96	6.98	0.7871	1.1688
DNA polymerase epsilon subunit 2	59.50	6.35	0.8302	1.1693
Retinal-specific ATP-binding cassette transporter	255.78	6.29	1.5031	1.1701
Amiloride-sensitive cation channel 5	57.43	8.19	1.0445	1.1704
Isoform ADelta10 of Lamin-A/C	70.62	8.40	1.0717	1.1707
Isoform 3 of Protein phosphatase Slingshot homolog 1	105.06	6.19	0.8391	1.1709
Isoform 2 of 5'-3' exoribonuclease 1	192.72	7.30	0.9502	1.1712
Conserved hypothetical protein	13.80	10.20	0.9826	1.1723
8 kDa protein	7.60	11.77	1.1214	1.1728
Isoform 1 of E3 ubiquitin-protein ligase RNF123	148.42	6.74	0.4911	1.1732
3-ketoacyl-CoA thiolase, peroxisomal	44.26	8.44	0.7042	1.1744

Protein FAM38B	62.63	7.74	1.4658	1.1745
Putative uncharacterized protein C10orf114	14.84	11.17	1.3583	1.1746
Protein-tyrosine phosphatase-like member B	28.62	9.54	0.9639	1.1756
174 kDa protein	174.30	7.03	0.9846	1.1758
Zinc finger protein 165	55.74	7.17	0.9166	1.1762
Coiled-coil domain-containing protein 129	98.88	5.01	0.7760	1.1767
similar to high mobility group nucleosomal binding domain 2 isoform 1	9.46	10.04	0.6699	1.1770
Isoform Monomeric of Arginyl-tRNA synthetase, cytoplasmic	67.10	6.55	0.9871	1.1772
RWD domain-containing protein 2A	33.87	6.47	1.0961	1.1775
similar to hCG1641491 isoform 2	22.89	10.56	1.0929	1.1785
ASCL3	20.91	8.54	1.2457	1.1786
Periplakin	204.53	5.57	0.8963	1.1786
DENN domain-containing protein 5A	147.00	6.70	0.8188	1.1789
rRNA 2'-O-methyltransferase fibrillarin	33.76	10.18	1.2390	1.1797
Isoform 2 of PAS domain-containing protein 1	72.69	5.38	1.0342	1.1820
Isoform 1 of Coiled-coil domain-containing protein FLJ25770	127.06	6.46	1.0536	1.1826
Immunity-related GTPase family Q protein	62.68	4.88	1.2634	1.1836
EF-hand domain-containing protein D2	26.68	5.20	1.1855	1.1843
Isoform 4 of Regulator of telomere elongation helicase 1	113.11	7.94	0.9467	1.1845
Isoform 2 of Cysteine-rich protein 2-binding protein	74.24	8.72	0.8864	1.1862
Isoform 2 of Vacuolar protein sorting-associated protein 13B	445.72	6.46	1.0687	1.1864
Isoform 1 of GAS2-like protein 1	72.67	10.05	1.1679	1.1867
Phenylalanyl-tRNA synthetase beta chain	66.09	6.84	1.1296	1.1875
Isoform 1 of Centrosomal protein of 164 kDa	164.21	5.36	1.4141	1.1878
Isoform 2 of Chromodomain Y-like protein	60.57	9.16	0.8572	1.1895
Isoform 1 of Abnormal spindle-like microcephaly-associated protein	409.54	10.45	1.0561	1.1900
Thyrotropin-releasing hormone receptor	45.06	8.28	0.8038	1.1907
similar to Vesicle-fusing ATPase	61.38	8.46		1.1909
34 kDa protein	34.41	9.91	0.8707	1.1912
Isoform 2 of VWFA and cache domain-containing protein 1	109.07	6.27	1.4992	1.1912
Probable phospholipid-transporting ATPase IC	143.63	7.15	1.0909	1.1920
Putative uncharacterized protein FAM83G	30.68	5.35	0.9818	1.1925
Putative protein FAM86B-like 1	32.77	6.32	1.3498	1.1929
Translational activator GCN1	292.56	7.43	0.8838	1.1934
Nuclear pore complex protein Nup107	106.31	5.43	0.9811	1.1943
Putative uncharacterized protein STX5	35.45	9.35	1.2637	1.1957
Toll-like receptor 5	97.77	6.68	0.8734	1.1966
Isoform 2 of PH and SEC7 domain-containing protein 3	115.86	5.99	2.0094	1.1971
Isoform C of Proline/serine-rich coiled-coil protein 1	38.77	11.21	1.2209	1.1975
Histone H2A type 2-A	14.09	10.90		1.1979
Isoform 1 of Nuclear mitotic apparatus protein 1	238.12	5.78	1.0174	1.1980
B-lymphocyte antigen CD19	61.09	4.98	0.7293	1.1990
24 kDa protein	23.60	9.09	1.0474	1.1994
Isoform 2 of Coiled-coil domain-containing protein 146	78.98	9.11	1.1175	1.1996
Nuclear receptor coactivator 5	65.50	9.60	0.8608	1.1998
PRO2492	10.82	8.47	2.2235	1.2000
Heterogeneous nuclear ribonucleoprotein G	42.31	10.05	1.0521	1.2007
Protein FAM175B	46.87	6.21	1.1176	1.2010
Putative uncharacterized protein	19.48	8.16	0.9586	1.2016
Keratin, type I cytoskeletal 9	62.09	5.30	0.5660	1.2029
Crk-like protein	33.76	6.74	1.0552	1.2043
23 kDa protein	23.18	6.38	0.8871	1.2060
PASK protein	122.97	5.01	0.6571	1.2067
cDNA FLJ58512, highly similar to Homo sapiens Mdm4, transformed 3T3 cell double minute 1, p53 binding protein, transcript variant 1, mRNA	76.78	8.75	0.9522	1.2071
27 kDa protein	27.02	10.17	1.1421	1.2075
Zinc finger and BTB domain-containing protein 25	48.96	6.55	1.0234	1.2081

Splicing factor 3 subunit 1	88.83	5.22	0.7952	1.2087
Isoform 2 of Poly [ADP-ribose] polymerase 8	91.10	8.37	1.5175	1.2096
GATS-like protein 2	36.03	5.16		1.2111
hypothetical LOC728701	31.11	10.73	0.9376	1.2112
cDNA FLJ53636, highly similar to Septin-7	48.61	8.63	0.8473	1.2112
Isoform 2 of Dedicator of cytokinesis protein 8	235.04	6.79		1.2123
Isoform 4 of Nebulin-related-anchoring protein	192.88	9.20	1.0041	1.2125
C-X-C motif chemokine 5	11.96	8.88	0.9421	1.2130
Isocitrate dehydrogenase 3, beta subunit isoform a precursor	42.85	8.46	0.8348	1.2132
Putative uncharacterized protein PRKCDBP (Fragment)	13.64	11.74	1.1040	1.2132
13 kDa protein	12.70	10.64	0.8786	1.2136
Putative dimethylaniline monooxygenase [N-oxide-forming] 6	61.25	6.39	1.1456	1.2140
8 kDa protein	8.47	4.59	0.9033	1.2146
PRED57 protein	12.61	9.52	0.7878	1.2164
Isoform 1 of RAF proto-oncogene serine/threonine-protein kinase	73.00	9.20	1.0642	1.2164
Acyl-CoA:lysophosphatidylglycerol acyltransferase 1	43.06	8.92	0.8155	1.2167
cDNA FLJ59851, highly similar to DNA methyltransferase 1-associated protein 1	25.22	10.01	1.1034	1.2194
Isoform 3 of Zinc finger protein 189	62.02	9.09	0.4255	1.2201
Isoform 2 of Serine/threonine-protein kinase WNK4	73.32	6.81	1.3341	1.2210
Pyroglutamyl-peptidase 1-like protein	21.66	8.09		1.2211
111 kDa protein	111.17	4.94	0.9937	1.2223
cDNA FLJ57537	27.86	10.87	0.8168	1.2231
Chromosome 9 open reading frame 58	10.49	6.09	0.4738	1.2234
Semaphorin-7A	74.78	7.64	1.0419	1.2248
82 kDa protein	81.98	8.56	1.1445	1.2253
Mediator complex subunit MED24 variant MED24_i11 (Fragment)	26.41	6.90	1.0708	1.2255
30 kDa protein	29.54	8.19	1.0173	1.2258
Isoform 2 of Calcyphosin-2	44.02	7.83	1.0164	1.2262
Contactin-3	112.77	6.30	1.2102	1.2277
cDNA FLJ55571, highly similar to Sad1/unc-84 protein-like 1	66.14	6.54	1.2434	1.2278
Histone H1.2	21.35	10.93	0.9451	1.2287
SPRY domain-containing SOCS box protein 2	28.61	8.18	1.0929	1.2291
Isoform 2 of Lysozyme-like protein 2	21.57	8.12	0.8352	1.2295
10 kDa protein	10.19	8.28	0.9341	1.2303
heterogeneous nuclear ribonucleoprotein R isoform 4	59.64	9.16	1.0893	1.2320
diacylglycerol lipase, beta isoform 2	59.80	8.07	1.2915	1.2321
Uncharacterized protein C8orf76	43.25	5.43	0.9180	1.2386
NFX.1	119.40	8.09	0.9300	1.2411
cDNA FLJ55723	101.05	7.80	0.8256	1.2421
H/ACA ribonucleoprotein complex non-core subunit NAF1	53.67	4.87	1.1942	1.2435
Isoform 2 of Synembryn-A	58.81	6.61	1.0040	1.2442
nuclear factor of kappa light polypeptide gene enhancer in B-cells 2 isoform b	96.62	6.25	0.9391	1.2445
Isoform Beta-4D of Integrin beta-4	194.33	6.13	0.9826	1.2445
Isoform 1 of Ankyrin-2	429.99	5.12	1.2679	1.2457
Pre-mRNA-processing factor 19	55.15	6.61	1.0996	1.2469
Putative uncharacterized protein C17orf87	13.70	9.88	0.9318	1.2475
Isoform 4 of Sushi, nidogen and EGF-like domain-containing protein 1	141.98	6.64	0.8554	1.2480
T-cell receptor alpha chain V region HPB-MLT	13.26	4.77	1.2144	1.2523
Uncharacterized protein C2orf78	96.94	9.00	1.1063	1.2552
Isoform 1 of Eukaryotic translation initiation factor 3 subunit B	92.42	5.00	0.9125	1.2595
122 kDa protein	122.16	5.58	1.1095	1.2602
65 kDa protein	65.27	5.19	1.6395	1.2605
cDNA FLJ54883, highly similar to Unc-13 homolog D	106.44	6.87	0.9667	1.2612
cDNA FLJ51643, moderately similar to Homo sapiens 1-aminocyclopropane-1-carboxylate synthase (PHACS), mRNA	14.54	7.74	1.0487	1.2618
Protein LTV1 homolog	54.82	4.91	1.1551	1.2633
Isoform 1 of Far upstream element-binding protein 3	61.60	8.38	1.2970	1.2642

NBPF8 isoform 5	90.22	4.63	0.8805	1.2644
Histone H3.2	15.38	11.27	1.0465	1.2649
G1 to S phase transition 1 isoform 2	68.53	5.33	0.8776	1.2651
Matrix metalloproteinase-14	65.84	7.77	1.0300	1.2653
Isoform 2 of Protein FAM119B	16.14	5.11	1.1702	1.2670
8 kDa protein	8.30	10.23	1.1822	1.2677
49 kDa protein	48.58	7.90	1.1121	1.2684
Histone-lysine N-methyltransferase SETD1A	185.92	5.14	1.0978	1.2690
Zinc finger protein 106 homolog	208.75	7.15	0.9759	1.2692
cDNA FLJ51770, highly similar to Microsomal triglyceride transfer protein large subunit	102.53	8.29	1.1371	1.2709
Isoform 4 of Serine/threonine-protein kinase WNK2	238.13	5.85	1.1135	1.2711
Isoform 4 of Mediator of RNA polymerase II transcription subunit 12-like protein	239.88	7.77	1.0037	1.2713
Isoform 4 of Protein SFI1 homolog	121.75	11.14	0.8712	1.2773
Ribosomal protein L15	24.48	11.78	1.3873	1.2783
Isoform 3 of Protein AHNAK2	605.27	5.36	1.0442	1.2789
Putative DMBT1-like protein	19.93	5.77	0.9483	1.2796
kinesin family member C3 isoform 3	77.04	6.65	0.6862	1.2800
Cytochrome c oxidase subunit VIb isoform 1	10.19	7.05	1.0851	1.2813
Isoform 3 of Immunoglobulin superfamily member 11	43.74	7.08	0.6865	1.2895
Isoform 5 of X-linked retinitis pigmentosa GTPase regulator-interacting protein 1	143.44	5.68	0.7944	1.2896
Isoform 1 of DEP domain-containing protein 7	58.27	7.77	1.2252	1.2899
Myosin-3	223.90	5.83	1.2331	1.2911
Isoform 1 of Diacylglycerol kinase zeta	124.05	9.04	0.9034	1.2917
intersectin 2 isoform 3	190.38	8.24	0.9478	1.2938
unc-13 homolog A	202.00	5.50	1.0335	1.2964
Uncharacterized protein C9orf127	76.33	8.75	0.7351	1.2966
dual specificity phosphatase 11	43.68	9.29	0.7278	1.2971
Isoform 1 of Serine/arginine repetitive matrix protein 2	299.44	12.06	1.6822	1.2988
EPS15 protein	83.60	4.70	1.1915	1.3068
Isoform 2 of DNA replication licensing factor MCM8	88.70	7.49	1.0933	1.3070
Microtubule-associated protein 1B	270.45	4.81	1.0921	1.3095
Isoform 2 of Protein Hook homolog 2	82.93	5.43	1.0556	1.3128
Isoform 2 of Zinc finger C3H1 domain-containing protein	217.35	8.03		1.3136
Isoform 1 of Glutaredoxin-2, mitochondrial	18.04	9.11	0.7897	1.3142
Isoform 2 of Kelch-like protein 4	80.78	6.81	1.0599	1.3179
Isoform 2 of Bromodomain adjacent to zinc finger domain protein 1B	170.34	8.48	0.9095	1.3204
Keratin, type II cytoskeletal 1	65.98	8.12	0.6044	1.3300
Fragile X mental retardation 1 neighbor protein	29.22	8.94	0.9020	1.3304
Dihydropyrimidinase-related protein 5	61.38	7.20	1.0347	1.3322
21 kDa protein	20.95	9.09	1.0735	1.3359
Isoform 2 of TBC1 domain family member 9	133.14	5.54	0.9988	1.3364
TAOK2 protein (Fragment)	64.32	9.76	0.9404	1.3399
13 kDa protein	12.52	10.14	1.2394	1.3437
Putative BCR-like protein 2	14.03	7.90	0.8887	1.3439
Syntaxin-19	34.30	7.39	1.3651	1.3458
cDNA FLJ38613 fis, clone HEART2006334, highly similar to Homo sapiens DiGeorge syndrome critical region gene 13 (DGCR13), mRNA	36.97	4.77	1.1358	1.3475
interferon regulatory factor 2 binding protein 2 isoform B	59.44	8.60	1.0321	1.3507
hypothetical protein LOC392145	18.58	4.75	0.9599	1.3531
Isoform 2 of FERM domain-containing protein 8	45.11	6.80	0.8649	1.3540
cDNA FLJ53542, highly similar to Heterogeneous nuclear ribonucleoproteins C	31.95	8.72	0.9038	1.3549
PRO2259	7.44	9.98	1.1928	1.3653
similar to hCG2040657	79.51	8.63	0.8775	1.3719
Similar to Gastric triacylglycerol lipase precursor	5.46	7.42	1.2869	1.3720
100 kDa protein	99.64	5.58	1.0148	1.3769
Isoform 2 of Lambda-crystallin homolog	33.34	6.00		1.3809

14 kDa protein	13.75	9.70	0.7996	1.3830
Isoform 2 of Double-stranded RNA-binding protein Staufen homolog 2	58.97	9.55		1.3843
Tumor necrosis factor receptor superfamily member 21	71.80	7.83	1.0033	1.3868
Histone H4	11.36	11.36	1.0590	1.3917
cDNA FLJ43187 fis, clone FCBBF3023443	13.69	11.46	1.7125	1.3933
ash1 (absent, small, or homeotic)-like	332.00	9.39	1.0014	1.3956
Isoform 4 of Spectrin beta chain, brain 3	243.27	5.67	0.8927	1.4068
Isoform 1 of Regulator of nonsense transcripts 2	147.72	5.69	1.0401	1.4095
Probable phospholipid-transporting ATPase IM	135.78	6.99	1.1071	1.4123
cDNA FLJ58633, highly similar to Leucine-rich repeat-containing protein 27	42.26	9.95	0.8298	1.4132
Protein FAM127A	13.16	5.07		1.4141
rhomboid domain containing 2 isoform b	23.67	9.77	1.1433	1.4203
Tubulin-specific chaperone E	59.31	6.76	0.8584	1.4244
Putative uncharacterized protein SLC27A4	26.15	5.87	0.7346	1.4262
Condensin-2 complex subunit D3	168.78	7.50	1.4627	1.4343
Isoform p56 of 59 kDa 2'-5'-oligoadenylate synthetase-like protein	59.19	7.87		1.4347
Keratin, type II cytoskeletal 8	53.67	5.59	0.9549	1.4366
LMNB1 protein	44.62	5.05	1.1972	1.4443
Isoform 1 of Integrin alpha-10	127.52	6.68	1.6590	1.4453
17 kDa protein	16.59	8.28	1.1767	1.4482
hypothetical protein LOC51306 isoform 3	90.52	4.92	0.7904	1.4524
cDNA FLJ60618, weakly similar to Zinc finger protein 3	53.34	8.84	0.9449	1.4659
similar to hCG2043419	15.96	10.39	0.8186	1.4686
Isoform 3 of Heterogeneous nuclear ribonucleoprotein H3	31.51	7.31	1.0708	1.4704
Putative uncharacterized protein FAM3A	21.15	8.16	1.1181	1.4710
Isoform 1 of Mitogen-activated protein kinase kinase kinase 7-interacting protein 2	76.45	8.54	0.5520	1.4747
cDNA FLJ60647, highly similar to Keratin, type II cytoskeletal 6B	55.70	8.43	0.9635	1.4798
Isoform 2 of Gamma-aminobutyric acid receptor subunit beta-3	54.34	9.10	1.2255	1.4845
Kinesin-like protein KIF3B	85.07	7.69	1.0150	1.4881
Isoform 4 of Protein inscuteable homolog	53.74	5.21	0.9018	1.4975
5 kDa protein	4.74	10.83	0.8888	1.5115
Protein	17.90	8.37		1.5232
FAM152A protein (Fragment)	12.21	10.39	1.4558	1.5279
keratin 77	61.86	5.99	0.6073	1.5281
48 kDa protein	47.98	7.77	1.1724	1.5286
Coagulation factor V	251.51	6.05	1.2398	1.5295
Olfactomedin-like protein 1	45.89	8.24	1.0150	1.5335
Histone H2A.V	13.50	10.58	1.0733	1.5370
cDNA FLJ52859, highly similar to Sperm-specific antigen 2	28.40	5.50	0.9110	1.5570
Keratin, type I cytoskeletal 10	59.47	5.21	0.6845	1.5718
Isoform 3 of Methyl-CpG-binding domain protein 4	60.91	8.95	1.1778	1.5851
RNA binding protein, autoantigenic	20.17	10.23	0.8262	1.5990
Isoform 1 of Cyclin N-terminal domain-containing protein 1	36.90	6.93	0.7003	1.6039
Putative uncharacterized protein CCDC125	36.14	7.37	1.3618	1.6222
Synaptopodin-2	117.44	8.57	1.2458	1.6357
Isoform 4 of Serine/threonine-protein kinase WNK1	206.48	6.15	1.0899	1.6611
Isoform 1 of Putative Polycomb group protein ASXL2	153.72	8.81	0.9601	1.6874
HLA-B associated transcript 2-like	242.82	8.34	1.0430	1.7075
Discoidin, CUB and LCCL domain containing 1	55.37	7.36	1.1784	1.7281
Putative uncharacterized protein FLJ45684	13.82	11.82	1.1085	1.7374
Isoform p59-HCK of Tyrosine-protein kinase HCK	57.28	6.86	0.7479	1.7450
Conserved hypothetical protein	19.63	11.39	1.0059	1.7471
Isoform 2 of ATP-binding cassette sub-family A member 10	173.12	6.65	0.8251	1.7501
Coiled-coil domain-containing protein 19	61.58	9.79	1.1193	1.7783
Progestin and adipoQ receptor family member 9	42.66	8.59	0.8468	1.7980
Keratin, type II cytoskeletal 2 epidermal	65.83	8.00	0.6020	1.8184

Isoform 2 of Phospholipase B1, membrane-associated	54.31	7.27	1.5613	1.8546
Isoform 1 of Hepatocyte growth factor receptor	155.43	7.33	0.9566	1.9026
Melanoma-associated antigen C3	71.86	4.87	1.2564	1.9147
Vimentin	53.62	5.12	1.0393	2.0310
Protocadherin beta-5	86.37	5.00	1.1736	2.0688
21 kDa protein	21.21	9.04	1.2826	2.2318
11 kDa protein	10.52	7.34	1.1159	2.2824
ubiquitin-conjugating enzyme E2C isoform 5	5.11	10.83	1.4188	2.2850
cDNA FLJ75256	22.06	7.96	1.0941	2.5028
Profilaggrin (Fragment)	133.53	9.64	1.2560	3.6792
Putative uncharacterized protein SDK1	239.85	6.34	0.9153	
Isoform B of Annexin A13	39.77	5.50	0.6534	
Receptor-type tyrosine-protein phosphatase eta precursor	151.76	6.18	0.4384	
cDNA, FLJ79367, highly similar to Homo sapiens MAX dimerization protein 3 (MXD3), mRNA	42.79	10.27	0.9998	
Putative uncharacterized protein ENSP00000340488 (Fragment)	30.93	4.59	0.8720	
hypothetical protein	15.31	8.02	1.2787	
Olfactory receptor 6C3	35.51	8.44	1.1711	
30 kDa protein	29.88	8.91	0.7042	
Hypothetical protein	18.09	9.16	0.3394	
